# Harnessing Maxwell’s demon to establish a macroscale concentration gradient

**DOI:** 10.1038/s41557-024-01549-2

**Published:** 2024-06-10

**Authors:** Jiratheep Pruchyathamkorn, Bao-Nguyen T. Nguyen, Angela B. Grommet, Miroslava Novoveska, Tanya K. Ronson, John D. Thoburn, Jonathan R. Nitschke

**Affiliations:** 1 Yusuf Hamied Department of Chemistry, University of Cambridge, Cambridge, UK; 2 Department of Chemistry, Randolph-Macon College, Ashland, VA USA

**Keywords:** Molecular capsules, Coordination chemistry

## Abstract

Maxwell’s demon describes a thought experiment in which a ‘demon’ regulates the flow of particles between two adjoining spaces, establishing a potential gradient without appearing to do work. This seeming paradox led to the understanding that sorting entails thermodynamic work, a foundational concept of information theory. In the past centuries, many systems analogous to Maxwell’s demon have been introduced in the form of molecular information, molecular pumps and ratchets. Here we report a functional example of a Maxwell’s demon that pumps material over centimetres, whereas previous examples operated on a molecular scale. In our system, this demon drives directional transport of *o*-fluoroazobenzene between the arms of a U-tube apparatus upon light irradiation, transiting through an aqueous membrane containing a coordination cage. The concentration gradient thus obtained is further harnessed to drive naphthalene transport in the opposite direction.

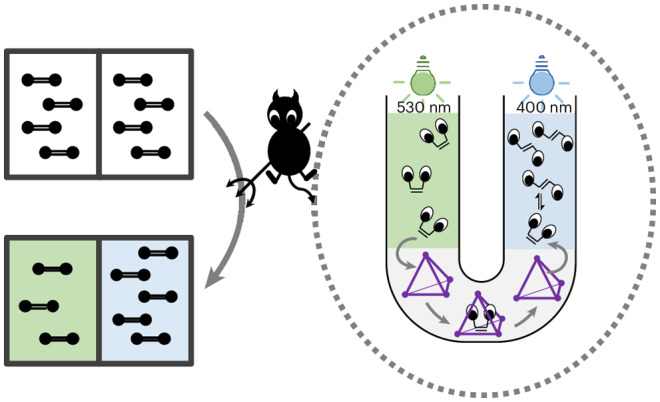

## Abstract (web summary)

In the original Maxwell’s demon thought experiment, a potential gradient of particles between two neighbouring compartments is created without the apparent use of work. Now a functional example of this experiment where material is pumped over centimetres has been reported—*o-*fluoroazobenzene is transported unidirectionally under light stimulation between two arms of a U-tube across an aqueous layer containing coordination cages.

## Main

In 1867, James Clerk Maxwell described a thought experiment that probed the limits of the second law of thermodynamics: a ‘demon’ gates the passage of particles between two neighbouring compartments, creating a potential gradient without appearing to do work. In the original thought experiment, two isolated compartments contain gas molecules at equal temperatures (or pressure) and are connected by a molecule-sized gate^1–5^. An active agent—the demon—selectively opens and closes the gate to partition hot and cold (or high velocity and low velocity) gas molecules into separate compartments. The demon thereby decreases the overall entropy of the system, creating a gradient that represents a source of potential energy. If the gate is frictionless, the demon appears to perform no work during this process—therein lies the paradox. A century later, Szilard developed a variation of Maxwell’s demon whereby a single gas molecule is hypothetically confined within a box and information about the molecule’s location is harnessed to produce work^6^. This formulation created a connection between Maxwell’s demon and information theory, allowing information to be considered as a physical property. The physicality of storage media thus implies that information must also obey the laws of thermodynamics as it is stored, transmitted and processed^7–9^. A demon must pay a thermodynamic cost to obtain information about individual molecules, thereby offsetting the reduction in entropy when creating a temperature gradient across the system. Furthermore, a physical demon’s capacity for remembering information about individual molecules must necessarily be finite; in forgetting this information to sort a new collection of molecules, heat must be dissipated.

Experimental analogues of Maxwell’s demon and molecular pumps have been developed^10–33^. A rotaxane-based molecular information ratchet, realized by David Leigh’s team, was arguably the first physical manifestation of a Maxwell’s demon. In Leigh’s system, a photoresponsive gate is positioned asymmetrically on the axle of a rotaxane, creating two neighbouring ‘compartments’ on either end^29^. Upon light irradiation, information regarding the proximity of the macrocycle to the gate drives unidirectional movement of the macrocycle across the axle. Whereas Leigh’s demon operates on a molecular scale, Raizen et al. demonstrated a similar principle within a system composed of many atoms, which reside in a potential well created by a magnet^34^, where a one-way gate composed of two optical beams plays the role of the demon, driving unidirectional movement of particles over an optical barrier and into a higher-energy compartment.

**Fig. 1:**
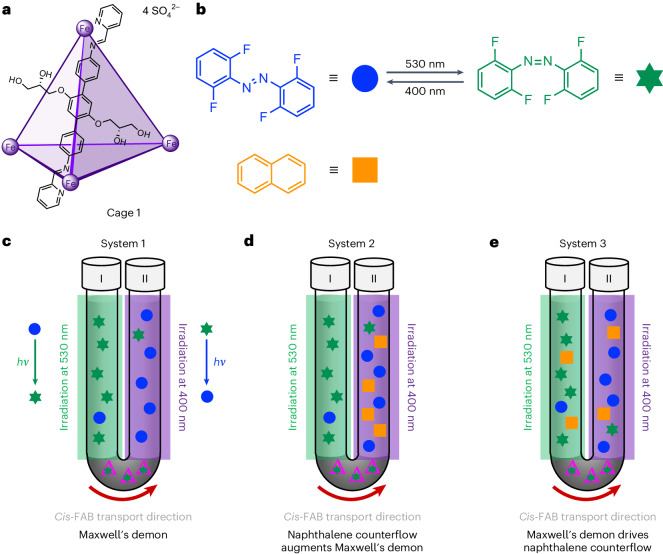
Illustration of experimental setup of the three directional transport systems. **a**, The structure of cage **1**, showing one of the six ligands that form the edges of the tetrahedron. **b**, Structures and corresponding symbols for *trans*-FAB, *cis*-FAB and naphthalene. *Trans*-FAB isomerizes to *cis*-FAB upon irradiation at 530 nm, while the reverse process occurs at 400 nm. **c**–**e**, Illustrations of the experimental U-tube configurations discussed later. System 1 relies on differential transport rates between the two FAB isomers to push the system away from equilibrium (**c**). System 2 couples an additional potential energy gradient, the presence of naphthalene in arm II, to drive the system further away from equilibrium (**d**). System 3 couples the establishment of a gradient of FAB across the membrane to the counterflow of naphthalene, driving the distribution of naphthalene out of equilibrium (**e**). Further experimental details can be found in Methods. Blue circles, *trans*-FAB; green asterisks, *cis*-FAB; orange squares, naphthalene; green-covered region indicates the tube area exposed to light at 530 nm; purple-covered region indicates the tube area exposed to light at 400 nm.

In this Article, we report a sorting system that drives the formation of an *o-*fluoroazobenzene (FAB) concentration gradient on the macroscale, across centimetres. As in Maxwell’s thought experiment, our system is composed of two neighbouring compartments, consisting of two layers of dodecane solvent in two arms of a U-tube apparatus (Fig. 1). Coordination cage **1** (Fig. 1a) functions as a molecule-sized, demon-attended gate. We also explore the addition of the competing guest naphthalene to push the system further out of equilibrium (Fig. 1b) and the use of our demon as a pump to create a naphthalene concentration gradient (Fig. 1c), as discussed below.

## Results and discussion

### Maxwell’s demon creates a concentration gradient

**Extended Data Fig. 1:**
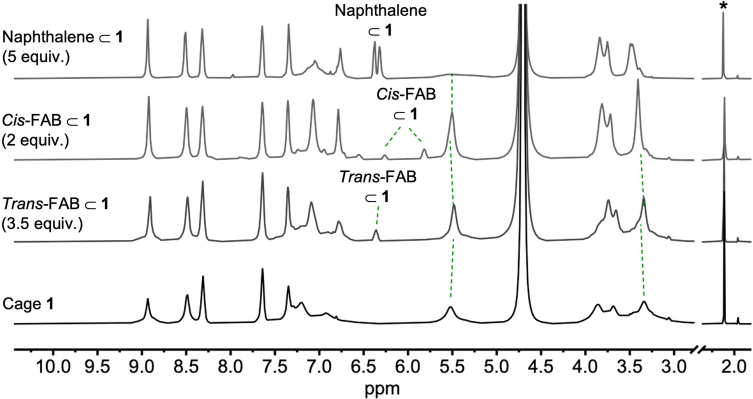
Naphthalene, *trans*- and *cis*-FAB hierarchy of encapsulation by **1** in water. ^1^H NMR (500 MHz, D_2_O) monitoring of the relative binding strength of *trans*- and *cis*-FAB and naphthalene to cage **1** in water. *Trans*-FAB was first introduced to the cage solution and guest encapsulation was subsequently observed. Upon *cis*-FAB addition, the *trans*-FAB ⊂ **1** signals disappeared while the *cis*-FAB ⊂ **1** signals were observed. Addition of naphthalene displaced the encapsulated *cis*-FAB from cage **1**. (* = CH_3_CN reference signal).

The two compartments within our system are separated by a bulk aqueous membrane containing Fe^II^_4_L_6_ coordination cage **1**, which transports a molecular cargo, the photoresponsive molecule FAB, between the two arms^35^. Crucially, *cis*-FAB has a higher affinity for cage **1** than *trans*-FAB, as indicated by the displacement of *trans*-FAB by *cis*-FAB from the cage cavity (Extended Data Fig. 1). In the system’s initial state, FAB is distributed equally between the dodecane solutions in the two arms. Upon irradiation of arm I at 530 nm, *trans*-FAB isomerizes to *cis*-FAB, which is preferentially extracted from arm I by cage **1**, transported through the aqueous layer and released into arm II. Subsequent re-isomerization of *cis*-FAB to *trans*-FAB—promoted by irradiation of the second compartment at 400 nm—impedes the molecule from returning to arm I. As the FAB molecules are directionally transported between the two arms, the system is driven away from equilibrium.

Our system was prepared by adding an aqueous solution of cage **1** (4 mM, 2.5 ml, 25 mol% relative to the total FAB in both arms) into the bottom of a glass U-tube (internal diameter 1.2 cm), and aliquots of a dodecane solution containing FAB (10 mM, 2 ml, 90% *trans*) into each arm. To monitor the concentration of FAB isomers in each arm, samples of each dodecane solution (0.3 ml) were withdrawn periodically for ^1^H nuclear magnetic resonance (NMR) analysis. The aqueous solution of cage **1** was stirred at room temperature for the duration of the experiment, while the dodecane layers were not. Following irradiation of arm I with light at 530 nm, a photostationary state containing 94% of *cis*-FAB was obtained. Arm II was simultaneously irradiated with 400 nm light, resulting in a photostationary state containing 92% *trans*-FAB (Supplementary Section 2).

**Fig. 2:**
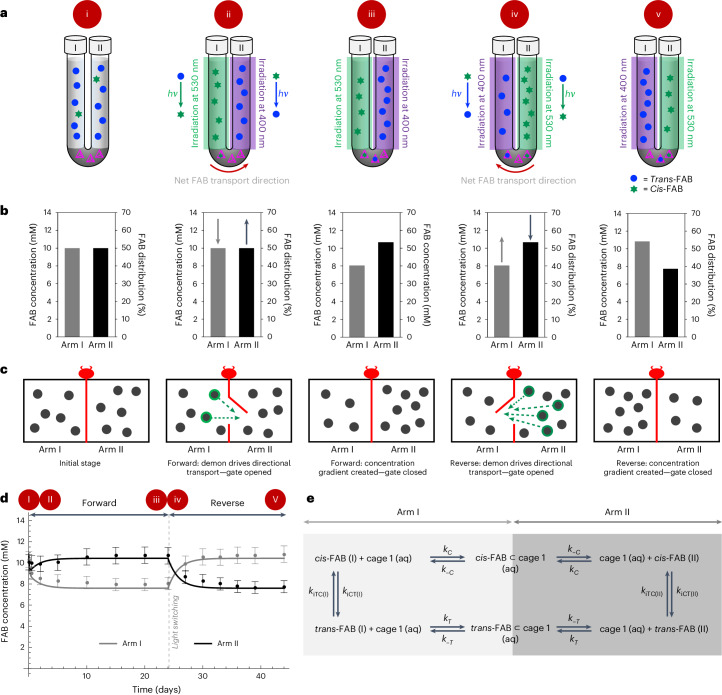
Statewise illustration and summary of results from the directional transport of FAB in system 1. **a**, U-tube configuration illustrating the distribution of *cis*-FAB (green asterisks) and *trans*-FAB (blue dots) between the two arms in system 1. **b**, Distribution plots of FAB concentration in arms I and II, illustrating the shifts in concentration away from the initial equilibrium state. **c**, Cartoon representation of the Maxwell’s demon system, showing how the demon gauges which FAB molecules to allow across the gate, resulting in the establishment of a FAB concentration gradient. **d**, Sum of *trans*- and *cis*-FAB concentrations in arm I (grey) and arm II (black) during initial forward transport and its subsequent reversal at day 24, with dots surrounded by error bars representing concentrations measured by ^1^H NMR, and solid lines showing the predictions of our model (Supplementary Section 10). The distinct stages studied are labelled i–v atop **a**; these stages are also shown in **b** and **c** stacked below the cartoons shown in **a** and in the time course shown in **d.** i, the initial equilibrium state of the experiment; ii, shortly after starting forward transport; iii, at the steady state of forward transport (day 24); iv, shortly after reversing the transport direction by changing which arm was illuminated by which wavelength of light and v, at the steady state of reverse transport (day 44). **e**, Our kinetic model of the system, with rate constants *k*_C_ and *k*_T_ for the uptake of *cis*-FAB and *trans*-FAB, respectively, by aqueous cage **1** from an organic phase; *k*_−C_ and *k*_−T_, the corresponding release rate constants for *cis*-FAB and *trans*-FAB, respectively, from **1**; *k*_*i*__CT(I)_ and *k*_*i*__TC(I)_ for the isomerization in arm I from *cis*- to *trans*-FAB, and *trans*- to *cis*-FAB, respectively; and *k*_*i*__CT(II)_ and *k*_*i*__TC(II)_ for the isomerization in arm II from *cis*- to *trans*-FAB, and *trans*- to *cis*-FAB, respectively. Data in **d** are presented as mean values ± measurement errors, derived from error propagation of the standard deviation and the signal-to-noise ratio (*n* = 22) of coronene (Supplementary Section 5). aq, aqueous.

At the start of the experiment, FAB was encapsulated by cage **1**, resulting in lower FAB concentration in both arms (Supplementary Section 7). After 10 days of continuous irradiation, a decrease in the total concentration of FAB was observed in arm I, together with an increase in arm II. No further changes in the sum of FAB isomer concentration between arms I (8 mM, 40%) and II (11 mM, 54%) were observed after 20 days, thus establishing a steady-state concentration gradient on the macroscale (Fig. 2). This process was also followed by ultraviolet–visible spectroscopy (UV–vis), confirming conclusions drawn based on NMR data (Supplementary Fig. 15). To confirm the net transport of FAB from arm I to arm II, a control experiment (Supplementary Section 14) was conducted. Two vertical tubes were set up, each analogous to the initial setup of arm I (Tube 1) and arm II (Tube 2), without the presence of the dodecane solution from the other arm. After irradiation, the decrease in the total concentration of FAB in tube 1 (1 mM, Supplementary Fig. 36), solely due to the effect of FAB sequestered within cage **1** in the aqueous layer, was less than that of arm I in system 1 (2 mM). This suggests that the increase in FAB concentration in arm II of the system was the result of the mass transport of FAB from arm I.

In the initial state of the system, each FAB molecule has an equal probability of residing in either dodecane compartment, corresponding to a state with high entropy (Fig. 2a–d, stage i). Light energy provided into the system promotes FAB isomerization from *trans* to *cis* in arm I, and *cis* to *trans* in arm II, thus driving preferential FAB transport through the gate in one direction (Fig. 2a–d, stage ii). As *cis*-FAB is transported more rapidly than the *trans* isomer (Supplementary Fig. 13), the overall FAB concentration decreases in arm I and increases in arm II. After crossing through the gate from arm I to arm II, *cis*-FAB transforms back into *trans*-FAB, a process corresponding to the demon ‘forgetting’ the position of the molecule as heat is released back into the system (Fig. 2a–d, stage iii). Light energy thus drives information processing to establish a concentration gradient between the two dodecane compartments, with no violation of the second law of thermodynamics.

Reversal of the stimuli applied to the two arms resulted in a reversal of the direction of FAB transport. After 24 days, arm I was irradiated at 400 nm to promote the relaxation of *cis-* to *trans-*FAB, and arm II was irradiated at 530 nm to isomerize *trans-* to *cis-*FAB (Fig. 2a–d, stage iv). This reverse transport of FAB was again observed to reach a steady state after 12 days, where the distribution of FAB between arm I (11 mM, 54%) and arm II (8 mM, 40%) mirrored its distribution following forward transport (Fig. 2a–d, stage v).

### Kinetic study and modelling

To investigate the kinetics of FAB transport, we developed a kinetic model (Fig. 2e) that considers the uptake and release of *cis*- and *trans*-FAB at the interfaces between the dodecane and aqueous layers, alongside the isomerization reactions between the two FAB isomers in each arm (Supplementary Section 10). The experimental data were least-squares fitted to our model (Fig. 2d), as described in Supplementary Section 10. The *cis*–*trans* and *trans*–*cis* isomerization rate constants in arms I and II (*k*_iCT(I)_ = 112.7 day^−^^1^, *k*_iTC(I)_ = 1,750 day^−^^1^, *k*_iCT(II)_ = 5,028 day^−^^1^ and *k*_iTC(II)_ = 437.2 day^−^^1^ during forward transport) were determined in separate experiments using NMR measurements to track the changes in FAB concentrations under irradiation at both 400 and 530 nm (Supplementary Section 2). The model gave the uptake and release rate constants for *cis*-FAB of *k*_C_ = 0.42 mM^−^^1^ day^−^^1^ and *k*_−__C_ = 1.69 day^−^^1^, and for *trans*-FAB, *k*_T_ = 0.29 mM^−^^1^ day^−^^1^ and *k*_−__T_ = 1.23 day^−^^1^, respectively.

**Extended Data Fig. 2:**
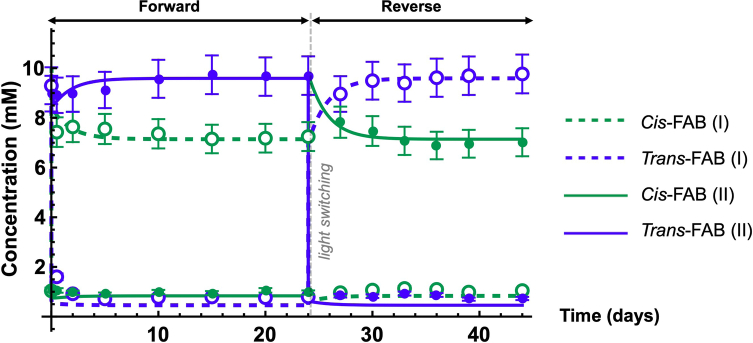
Model optimization for System **1**. Concentrations of *cis*-FAB (green) and *trans*-FAB (blue) in arm I (hollow dots and dashed line) and arm II (solid dots and solid line) during the forward and reverse transport in System 1, showing experimental results measured by ^1^H NMR (dots), error bars and model predictions (lines) for each arm. In the forward transport, *cis*-FAB was observed to flow from arm I to arm II where it was isomerized to *trans*-FAB. Upon switching the light stimuli in the reverse transport, the flow of *cis*-FAB was reversed (from arm II to arm I) and the subsequent isomerization to *trans*-FAB was happening in arm I. These processes caused the shift in the total FAB concentrations. The grey dashed line indicates the point which the LED light strips were swapped, switching from forward to reverse transport.

These rate constants indicate that the rate of *cis-*FAB uptake by cage **1** is higher than for *trans-*FAB. The uptake of *cis-*FAB from arm I is thus faster than that of *trans-*FAB from arm II at the beginning of the experiment (Extended Data Fig. 2). Cage **1** thus transports *cis-*FAB from arm I and releases it to arm II. Since the isomerization rate constant of *cis-* to *trans-*FAB is also three orders of magnitude greater than the *cis-*FAB uptake rate constant, *cis-*FAB relaxes to *trans-*FAB quickly due to irradiation at 400 nm after it is released into arm II during the forward transport, resulting in the accumulation of *trans-*FAB in arm II, as observed experimentally.

Furthermore, we hypothesized that the release of a guest bound within **1** would be facilitated by competitive displacement. When *cis*-FAB is transported from arm I to arm II in isolation, the driving force for cargo egress would thus be smaller than in the presence of *trans*-FAB in arm II (Supplementary Section 13), where egress of *cis*-FAB may be facilitated by competitive displacement by *trans*-FAB, and vice versa. This rationale implies a limit to the degree to which this system may be driven out of equilibrium, as the transport of *cis*-FAB from arm I to II is partly offset by reverse transport of *trans*-FAB from arm II to I.

### The presence of naphthalene

As in Maxwell’s original thought experiment, our initial system is driven out of equilibrium solely upon inputting light energy. If the egress of *cis*-FAB into arm II is facilitated by competitive displacement, however, the limits of this experimental setup could be overcome by adding a competing guest to arm II, thereby creating an even larger FAB concentration gradient. We selected naphthalene as this competing guest, which binds more strongly to cage **1** than either *trans*- or *cis*-FAB (Extended Data Fig. 1)^36^.

**Fig. 3:**
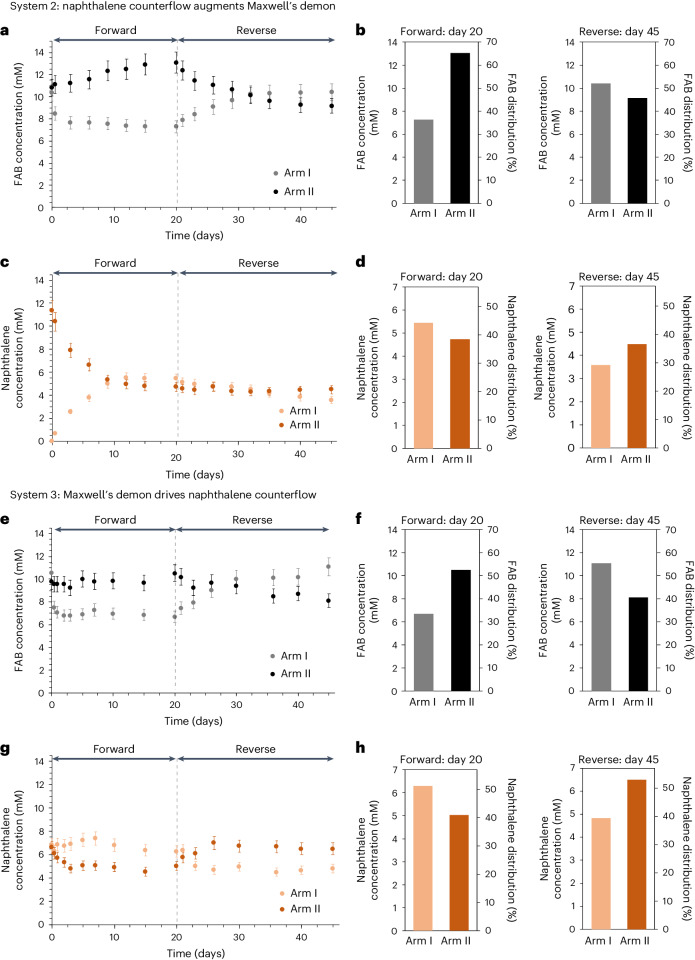
Naphthalene and FAB distributions in two arms of the U-tubes in systems 2 and 3. **a**,**e**, Concentration of FAB measured by ^1^H NMR in arm I (grey dots) and arm II (black dots) in system 2 (**a**) and system 3 (**e**) during initial forward transport and its reversal at day 21. **b**,**f**, Distribution charts of FAB concentration in arm I (grey bars) and arm II (black bars) in system 2 (**b**) and system 3 (**f**) at the end of the forward (day 20) and reverse (day 45) processes, illustrating the shifts in concentration away from the initial equilibria in systems 2 and 3. **c**,**g**, Concentration of naphthalene measured by ^1^H NMR in arm I (light orange dots) and arm II (dark orange dots) in system 2 (**c**) and system 3 (**g**) during forward and reverse transport for systems 2 and 3. **d**,**h**, Distribution charts of naphthalene concentration in arm I (light orange bars) and arm II (dark orange bars) in system 2 (**d**) and system 3 (**h**) at the end of the forward (day 20) and reverse (day 45) process, illustrating the shifts in concentration away from the initial equilibrium in systems 2 and 3. Data are presented as mean values ± measurement errors, derived from error propagation of the standard deviation and the signal-to-noise ratio (*n* = 22) of coronene (Supplementary Section 5).

Using a U-tube apparatus analogous to those described previously, system 2 (Fig. 1d) was set up, in which a solution of *trans*-FAB (10 mM) in dodecane was loaded into arm I, and naphthalene (11 mM) was loaded together with *trans*-FAB (10 mM) into arm II. Arms I and II were continuously irradiated with light at 530 nm and 400 nm, respectively. As we hypothesized, the rate of *cis*-FAB egress into arm II increased in the presence of naphthalene (Supplementary Section 8). Furthermore, the net transport of FAB to arm II was accompanied by a net transport of naphthalene to arm I. The redistribution of both species plateaued after 20 days (Fig. 3a–d). The final overall concentrations of FAB in arms I and II were 7.3 mM (36%) and 13 mM (64%), respectively, and the final concentrations of naphthalene in arms I and II were 5.4 mM (48%) and 4.7 mM (42%), respectively.

Notably, in naphthalene-containing system 2, the difference in the overall concentration of FAB between arms I and II was 6 mM, greater than the difference (3 mM) in system 1, where naphthalene was absent. Furthermore, the sum of the concentrations of FAB in arms I and II is higher in the presence (20 mM) than in the absence (19 mM) of naphthalene (Supplementary Fig. 16), leading us to infer that the introduction of naphthalene in arm II not only drove redistribution of FAB but also reduced the amount of FAB stored within the aqueous membrane. In this system, the driving force supplied by information collected by the demon is supplemented by an additional source of energy: the potential energy associated with applying a concentration gradient of naphthalene across the two arms. The thermodynamic cost associated with pushing the FAB concentration gradient further from equilibrium is thus paid using a corresponding increase in the entropy of the competing cargo, naphthalene.

As with system 1, we sought to reverse the direction of FAB transport by reversing the light stimuli applied to arms I and II, monitoring the process using ^1^H NMR (Fig. 3a) and UV–vis (Supplementary Fig. 18). The final difference in FAB concentration between arms I and II (1 mM), was less than for the forward process, an effect that we attribute to the varying distribution of naphthalene over the course of the experiment. While the forward process began with naphthalene present solely in arm II, the reverse process began with naphthalene distributed almost equally between the arms (arms I and II contain 5.4 mM and 4.7 mM of naphthalene, respectively). Unlike the forward process, wherein the potential energy associated with the naphthalene concentration gradient drove the system further out of the initial equilibrium, the reverse process lacks that initial source of potential energy and relies primarily on the *cis*-FAB concentration gradient supplied by the demon.

Using our kinetic model to predict the behaviour of system 2, we initially assumed that naphthalene impacts the system by reducing the number of available cages for FAB transport, reducing the available concentration of cage **1** by a factor of *a*. Thus, only *a*·[cage **1**]_initial_ of cage **1** was considered to be active, where 0 ≤ *a* ≤ 1. The resulting model predicted a larger FAB concentration difference between the two arms for lower *a* at the steady state (Supplementary Section 11), which agrees with our findings for forward transport (Fig. 3a). A reduced rate of FAB transport in the reverse process was observed as more naphthalene was encapsulated in the cage. Experimental configurations with the same total amounts of naphthalene should thus have the same *a* values, thus leading to the same FAB concentration differences at a steady state. The model was further refined by considering naphthalene transport over time and the competitive displacement mechanism (Supplementary Figs. 29–32), which provides insight into naphthalene transport and the difference in naphthalene concentration at the end of the forward and the reverse transport of system 2 (Fig. 3d).

On the basis of this prediction, we then investigated system 3 (Fig. 1e), where naphthalene was introduced into both arms of the U-tube, at a concentration (6 mM) such that the total amount of naphthalene was equivalent to the amount used in system 2. The FAB concentration was kept at 10 mM in each arm. We then irradiated arm I with light at 530 nm and arm II with light at 400 nm. In agreement with the prediction of our model, the final distribution of FAB in arms I and II of system 3 was further from equivalence than in the absence of naphthalene in system 1, with the forward and reverse processes plateauing at 33% (6.7 mM) and 55% (11 mM) in arm I, and 53% (11 mM) and 40% (8.1 mM) in arm II, respectively (Fig. 3e,f).

Remarkably, we also observed unequal transport behaviour for naphthalene in system 3 (Fig. 1e). The transport of FAB from arm I to II was accompanied by a disproportionally greater net transport of naphthalene from arm II to arm I. A maximum difference was observed in naphthalene concentration of 52% (6.3 mM) in arm I, and 42% (5.0 mM) in arm II (Fig. 3g,h). A similar, but opposite, concentration difference was observed in the reverse transport of system 3, following inversion of the illumination.

Expanding the analogy between our system and Maxwell’s original thought experiment, this experiment offers an example whereby two different species, A and B, are distributed equally throughout two compartments. Despite the demon being blind to the location of species B, the act of collecting information about species A allows B to be driven out of equilibrium. The coincidence of this transient concentration gradient with the induction period of the system suggests that the molecular gate in our system, that is, cage **1**, plays an active role in regulating passage of FAB and naphthalene across the aqueous membrane.

As the experiment began, FAB and naphthalene competed to bind within empty cage **1**. The different stimuli applied to arms I and II, however, make the competitive processes at each interface also different. At the water–dodecane interfaces in arms I and II, naphthalene primarily competes with *cis*-FAB and *trans*-FAB, respectively. Given that *trans*-FAB is the least competitive binder among these three species, we expected to observe a greater degree of naphthalene ingress at interface II than at interface I. Likewise, we would expect to observe more naphthalene egress at interface I than at interface II, as naphthalene is competitively displaced by *cis*-FAB (Supplementary Section 11 and Supplementary Figs. 30–33). Taken together, these two effects explain the rapid increase in the concentration of naphthalene in arm I during the induction period.

## Conclusions

Our demon can thus be considered as a simple machine, whereby passage through a gate is regulated by changing the state of a molecule^21,24,37–48^. This type of demon has been described in terms of an energy ratchet^49–51^ or, in analogy with a system filled with flying umbrellas, divided into two compartments using a barrier containing evenly spaced bars^52^. Initially, all of the umbrellas are closed, they are narrow enough to pass between the bars, and thus they distribute themselves equally between the compartments. Upon applying a stimulus to one compartment, its umbrellas pop open, become too large to pass between the bars and begin to accumulate in that compartment, thereby establishing a gradient of umbrellas. Instead of umbrellas, one could also imagine two geometrically dissimilar isomers, whereby one isomer could pass through a molecular gate, while the other would be sterically hindered. Our system relies on differences in binding thermodynamics and kinetics experienced by *trans*-FAB or *cis*-FAB and cage **1**.

Having established a concentration gradient using our demon, we also introduced a strategy to drive the system further from equilibrium by adding a competitive species, naphthalene, initially into one arm only (Fig. 1, System 2). Significantly, by starting with a system containing naphthalene in both arms, we were able to harness the demon to pump naphthalene selectively from one side to the other. Such light-driven selective pumping of chemical species across a membrane may prove useful in the context of chemical separations, the development of new separations methods having been identified as a key challenge to the decarbonization of the world economy^43^.

## Methods

### Cage 1 synthesis

Cage **1** was synthesised using the previously reported protocol^53^. The scheme for the synthesis of the diamino terphenylene subcomponent is provided in the Supplementary Section 1.

FeSO_4_ • 7 H_2_O (22.2 mg, 0.0800 mmol, 4 equiv.), the diamino terphenylene subcomponent (52.9 mg, 0.120 mmol, 6 equiv.) and 2-formylpyridine (22.9 µl, 0.240 mmol, 12 equiv.) were placed in a 20 ml vial in a glovebox. A total of 5.0 ml degassed D_2_O and 5.0 ml dry CH_3_CN were added, and the mixture was stirred for 12 h at room temperature. The solvent was then removed at low pressure at 25 °C. The concentrated solution was washed with diethyl ether (5 ml × 3) and dried to yield cage **1** (0.0200 mmol, ca. 100%) as a purple solid. D_2_O was added quickly to prepare 4.0 mM solution of cage **1** for the experiment. ^1^H NMR (500 MHz, D_2_O, 298 K): *δ* = 8.83 (broad s, 12H, H_8_), 8.42 (unresolved d, 12H, H_10_), 8.25 (unresolved dd, 12H, H_12_), 7.58 (unresolved dd, 12H, H_11_), 7.25 (unresolved d, 12H, H_13_), 7.06 (unresolved s, 24H, H_5_), 6.78 (unresolved s, 12H, H_2_), 5.39 (broad s, 24H, H_6_), 3.76 (unresolved s, 24H, H_14_), 3.60 (unresolved s, 12H, H_15_) and 3.26 (unresolved s, 24H, H_16_). See Supplementary Fig. 2 for NMR spectrum and proton assignments.

### *o*-TetraFAB synthesis

FAB was synthesised using an optimized version of a reported protocol^54^.

The 2,6-difluoroaniline (275 μl, 350 mg, 2.73 mmol, 1 equiv.) and lead (IV) acetate (3.05 g, 6.83 mmol, 2.5 equiv.) were dissolved in CHCl_3_ (25 ml) and refluxed at 100 °C for 1.5 h followed by overnight stirring at room temperature. The reaction mixture was subsequently filtered through celite. The solvent was then evaporated under dynamic vacuum. The crude product was purified through an SiO_2_ column (dichloromethane: cyclohexane, solvent ratio 1:3) yielding a bright orange solid (64 mg, 20%), which is a mixture of *trans*- and *cis*-tetraFAB (ratio 9:1). ^1^H NMR (400 MHz, CDCl_3_, 298 K) *δ*_H_ (ppm) *trans*-FAB was 7.38 (*t*, 2H, H_1−__*trans*_) and 7.07 (*t*, 4H, H_2−__*trans*_), and *cis*-FAB was 7.19 (*m*, 2H, H_1−__*cis*_) and 6.85 (*m*, 4H, H_2−__*cis*_). ^13^C{^1^H} NMR (126 MHz, CDCl_3_, 298 K) *δ*_C_ (ppm) was 156.55 (C_4−__*trans*_), 154.41 (C_3−__*trans*_) and 131.29 (C_2−__*trans*_), 112.56 (C_1-*cis*_). ^19^F{^1^H} NMR (471 MHz, CDCl_3_, 298 K) *δ*_F_ (ppm) was −119.64 (F_*cis*_) and −121.48 (F_*trans*_). For NMR spectra and assignments, see Supplementary Fig. 3.

### Light-gated FAB transport experimental setup and measurements

Each experiment was prepared by adding an aqueous solution of cage **1** (4 mM, 2.5 ml and 25 mol% relative to the total FAB in both arms) into the bottom of the U-tube (internal diameter 1.2 cm). Both arm I and arm II contained FAB (*trans*/*cis* mixture ratio 9:1) solutions at an equal concentration of 10 mM in dodecane (2 ml). The solutions contained coronene (0.25 mM) as an internal standard. In addition, arm II contained triisopropylbenzene (10 mM) as an indicator. The role of this indicator is to ensure that no physical mixing of the dodecane solutions occurs between arms I and II, and that the transport phenomenon observed in this study is thus the result of transportation through the cage layer. Triisopropylbenzene was chosen as an indicator for two reasons. First, as triisopropylbenzene was not transported by the cage^37^, this compound would thus remain in arm II and would not interfere with the guest transport process. Second, triisopropylbenzene solution in dodecane shows an absorption peak in the region 200–250 nm, which does not overlap with the absorption region of *trans*- and *cis*-FAB. The cage layer was stirred at 250 rpm at room temperature with a cylindrical magnetic stir bar (3 × 6 mm).

*Trans-*to-*cis*-FAB isomerization was promoted using light-emitting diode light strips with a wavelength of 530 nm and with luminous flux of 250 lumen m^−1^ and power of 2.4 W. The reverse reaction, *cis* to *trans*, was promoted when irradiated using light-emitting diode light strips at the wavelength of 400 nm and with luminous flux of 200 lumen m^−1^ and power of 7.2 W. The light strips were wrapped around the U-tube arms.

Light irradiation in both arms was carried out simultaneously. Arm I was irradiated at 530 nm, expecting *trans-*to-*cis* isomerization. Arm II was irradiated at 400 nm promoting *cis-*to-*trans* relaxation. The two arms were isolated by a black partition and covered to avoid exposure to external sources of light. The system was flushed continuously with nitrogen gas to maintain the experiment at room temperature. Photos of the setup are provided in Supplementary Section 15 and Supplementary Fig. 37.

During the experiments, NMR and UV–vis measurements were taken regularly; 0.3 ml of solution from each of the dodecane phases (arms I and II) was taken for measurements. Each solution was put into an NMR tube, covered in aluminium foil to avoid external light exposure. NMR measurements were then taken before transferring the solutions into cuvettes for UV–vis measurements. The solutions were then put back into the arm of the U-tube from which they had been taken out. Care was taken to avoid external light during all transfers and measurements, by covering the samples with aluminium foil. The process of removing, measuring and returning the solutions to the U-tubes was carried out in less than 30 min in all cases, to minimize thermal isomerization.

Further details on the NMR and UV–vis spectrometers can be found in Supplementary Information. This general procedure was used for all experiments (systems 1–3) in this study.

## Online content

Any methods, additional references, Nature Portfolio reporting summaries, source data, extended data, supplementary information, acknowledgements, peer review information; details of author contributions and competing interests; and statements of data and code availability are available at 10.1038/s41557-024-01549-2.

## Supplementary information

Supplementary Information Supplementary Text 1–15, Supplementary Figs. 1–37 and Tables 1–8.

Supplementary Data 1 Raw data of the NMR and UV measurements for systems 1, 2 and 3.

## Data Availability

All data is available in the main text or the supplementary materials.

## Code availability

Mathematica code is available in Supplementary Information.
