# Supplementary material for: Harnessing Maxwell’s demon to establish a macroscale concentration gradient
Source: Nat Chem. 2024 Jun 10;16(9):1558–64. doi: 10.1038/s41557-024-01549-2 (PMC11374679; doi:10.1038/s41557-024-01549-2)
Supplement: Supplementary file 1 — Supplementary Text 1–15, Supplementary Figs. 1–37 and Tables 1–8. [file 41557_2024_1549_MOESM1_ESM.pdf]

nature chemistry

Supplementary information https://doi.org/10.1038/s41557-024-01549-2

Harnessing Maxwell's demon to establish a macroscale concentration gradient

In the format provided by the authors and unedited

Table of Contents

Materials and Methods 2

Supplementary Text 3

1. Cage 1 and *O*-tetrafluoroazobenzene (FAB) 3

2. Isomerization experiments of FAB 5

3. *Trans*- and *cis*-FAB encapsulation by cage 1 in water 8

4. Naphthalene, *trans*- and *cis*-FAB hierarchy of encapsulation by 1 in water 10

5. Quantitative analysis by ^{1}H NMR and uncertainty calculation 11

6. FAB UV-Vis absorption: calibration and peak fitting 16

7. Maxwell's Demon experiment (System 1): experimental results 18

8. Naphthalene Counterflow Augments Maxwell's Demon experiment (System 2): experimental results 23

9. Maxwell's Demon Drives Naphthalene Counterflow experiment (System 3): experimental results 29

10. Model prediction for Maxwell's Demon experiment (System 1) 35

11. Model prediction for Naphthalene Counterflow Augments Maxwell's Demon experiment (System 2), and Maxwell's Demon Drives Naphthalene Counterflow experiment (System 3) ... 42

12. *Trans*- and *cis*-FAB transport without cage 1 50

13. Independent transport of *trans*- and *cis*-FAB 51

14. Control experiment to confirm FAB transport in the presence of light 52

15. Photos of the experimental setup 55

References 57

Materials and Methods

Materials:

Unless stated otherwise, all the reagents were purchased from commercial sources and used without further purification. *O*-tetrafluoroazobenzene, FAB, was synthesized as reported in the literature[1].

Methods:

^{1}H and ^{13}C NMR spectra were recorded using a Bruker 500 MHz AVIII HD Smart Probe spectrometer. ^{19}F NMR was recorded using a Bruker 400 MHz Avance III HD Smart Probe NMR spectrometer. Chemical shifts were reported in ppm. All the spectra were measured at 298 K, unless stated otherwise. ^{1}H and ^{13}C signals were referenced to the residual solvent peak. ^{19}F NMR spectra were referenced to octafluoro-9,10-bis[4-(trifluoromethyl)phenyl]anthracene at −64 ppm. The ^{1}H NMR spectra of the material transported through the cage membranes were referenced to an internal standard of coronene in D_2O at 9.1 ppm. The experiments were performed on a Bruker 500MHz AVIII HD spectrometer, equipped with a broadband (31P-109Ag) 'BBFO' probe, running Topspin 3.2. A nominal 30 ° pulse, an irradiation frequency corresponding to 15 ppm were used, digitizing a 10,000 Hz frequency range with a 64 K resolution over 3.28 seconds. A relaxation delay of 1 seconds was used to give a total pulse recycle time of 4.28 seconds. Every NMR experiment was recorded with a constant 32 scans. Receiver gain is set at 12.7. The results were processed using the MNova (versions 14.1.1-24571 and 11.0.4-18998) software package and were Fourier transformed at the observed resolution of 64 K, using an exponential 'line broadening' function of 1 Hz. Manual phase correction was used, along with an 'ablative' baseline correction algorithm to allow accurate integration with the software's auto-linear function. The accuracy of the baseline correction and integration can further be improved by only transforming a limited set of frequencies from 9.8 to 6.6 ppm.

UV-Vis measurements were performed on an Agilent Technologies Cary Series UV-Vis-NIR and Agilent Technologies Cary 60 UV-Vis spectrophotometers. Unless stated otherwise, quartz cuvettes of 1 mm path length were employed, and the spectra were recorded at room temperature. Spectra were recorded in dual beam mode, using only the front analyte beam to record spectra and leaving the rear beam open to air. Background measurements containing only solvent used were conducted. These backgrounds were subtracted from the sample data.

Supplementary Text

1. Cage 1 and *O*-tetrafluoroazobenzene (FAB)

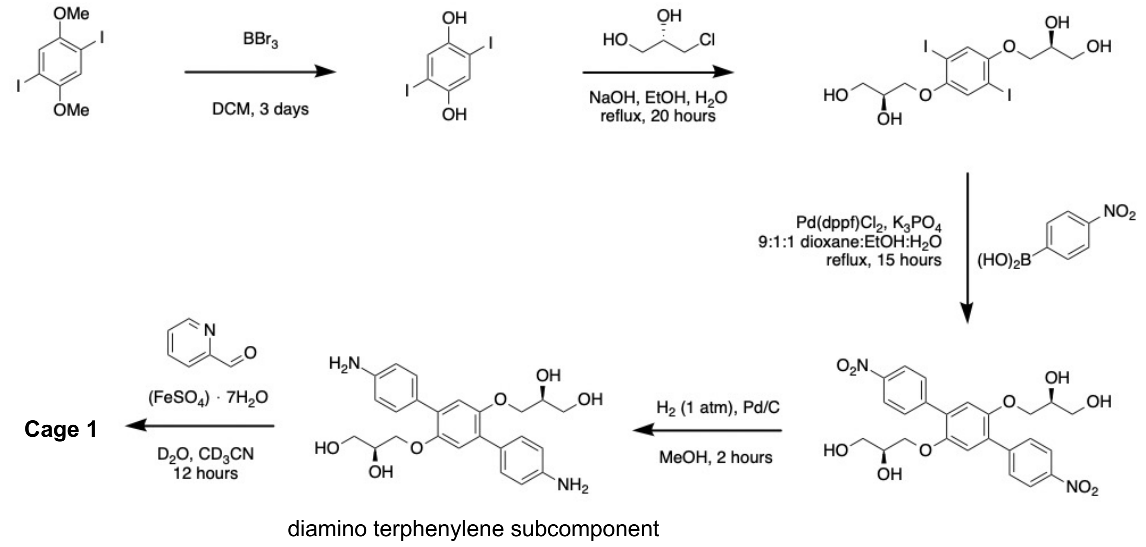

Supplementary Fig. 1. Synthetic scheme for cage **1** from diamino terphenylene subcomponent, which can be prepared from 1,4-diiodo-2,5-dimethoxybenzene following the literature procedure[1].

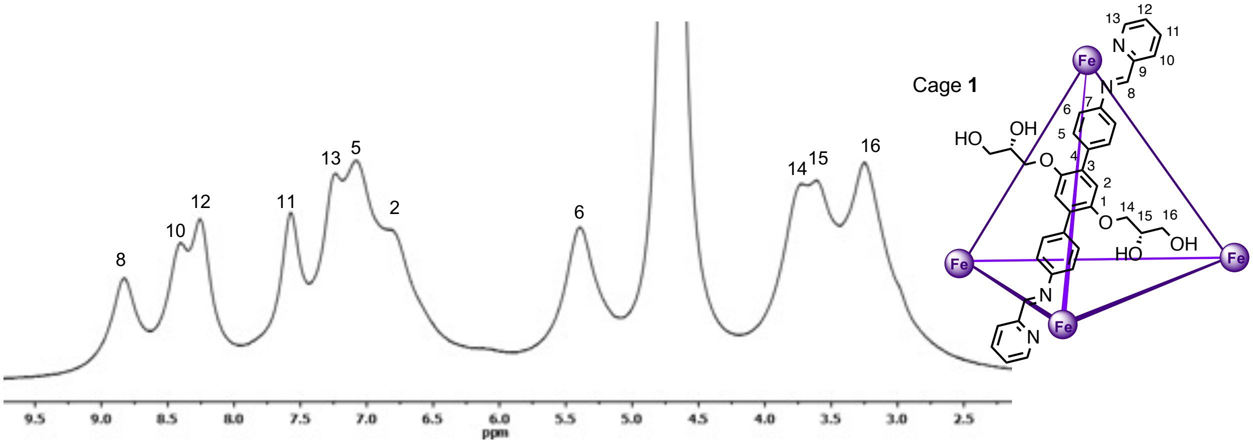

Supplementary Fig. 2. ^{1}H NMR (500 MHz, D$_2$O, 298 K) of cage **1**.

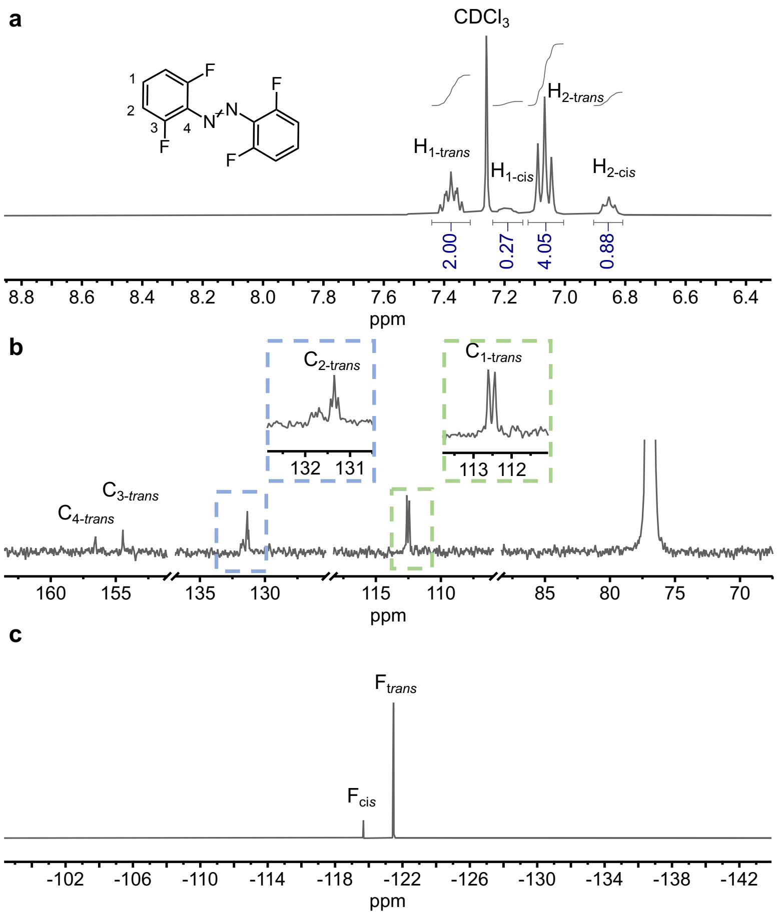

Supplementary Fig. 3. a, ^{1}H NMR (400 MHz, $CDCl_3$, 298 K); **b**, $^{13}C\{^{1}H\}$ NMR (126 MHz, $CDCl_3$, 298 K); **C**, $^{19}F\{^{1}H\}$ NMR (471 MHz, $CDCl_3$, 298 K) of *trans*-FAB product containing a trace of *cis*-FAB impurity.

2. Isomerization experiments of FAB

Irradiating *trans*-FAB solution (0.5 mL, 10mM) at 530 nm for 1 h promoted a 94 % conversion to *cis*-FAB. To trigger the reverse process, irradiating the sample at 400 nm for 1 h allowed the reformation of 92 % *trans*-FAB (Supplementary Fig. 4b).

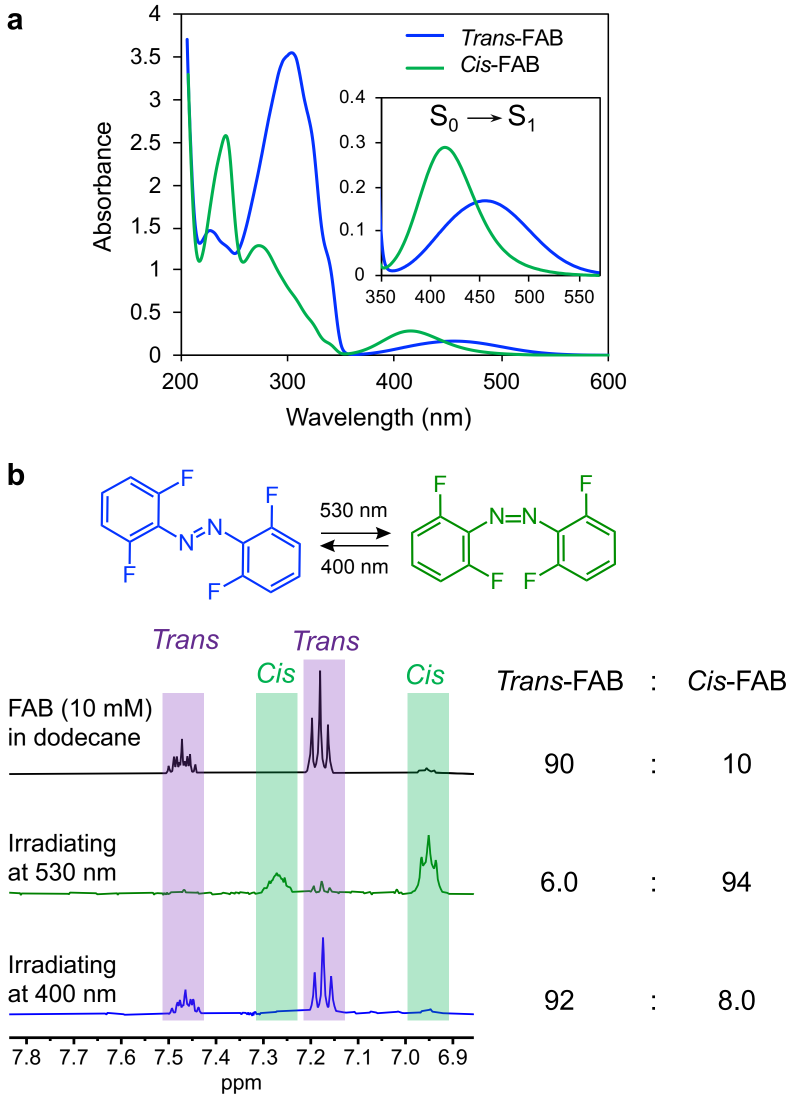

Supplementary Fig. 4. a, UV-Vis absorption of *trans* and *cis*-FAB (10 mM). **b**, Light irradiation triggered the nearly full conversion of *trans*- and *cis*-FAB isomerization. The isomer percentages are determined by ^{1}H NMR integration.

Kinetic studies of the isomerization of FAB was carried out by monitoring the change in concentrations of *trans*- and *cis*-FAB upon irradiation at 530 nm and 400 nm (Supplementary Fig. 5). The results were analysed according to the relationship below, to obtain the rate constants for FAB isomerization processes of *trans*- to *cis*-FAB (k_{iTC}) and *cis*- to *trans*-FAB (k_{iCT}) under irradiation at 530 nm and 400 nm.

For irradiation at 530 nm, concentrations of *trans*-FAB were monitored over time. From the chemical reaction in Supplementary Fig. 5e, we can write the change in concentration of *trans*-FAB as:

$$\frac{d[Trans]}{dt} = -k_{iTC,530nm}[Trans] + k_{iCT,530nm}[Cis] \quad \textbf{(Eq. 1)}$$

Solving differential equation for the kinetics of the reversible reaction, we can write a relationship:

$$\ln\left([Trans]_t - [Trans]_{eq}\right) = \ln\left([Trans]_0 - [Trans]_{eq}\right) - \left(k_{iTC,530nm} + k_{iCT,530nm}\right)t; \quad \textbf{(Eq. 2)}$$

where $[Trans]_t$ = concentration of *trans*-FAB at day t (mM),
$[Trans]_{eq}$ = concentration of *trans*-FAB at equilibrium (mM),
$[Trans]_0$ = initial concentration of *trans*-FAB (mM),
$k_{iTC,530nm}$ = rate constant for isomerization of *trans*- to *cis*-FAB at 530 nm (s^{-1}),
$k_{iCT,530nm}$ = rate constant for isomerization of *cis*- to *trans*-FAB at 530 nm (s^{-1}),
t = time (s).

Plotting $\ln([Trans]_t - [Trans]_{eq})$ against t gives a linear relationship with gradient $-(k_{iTC,530nm} + k_{iCT,530nm})$. The individual rate constants can be calculated from the gradient and the equilibrium constant for the isomerization at 530 nm ($K_{iT\text{-}C,\ 530nm}$), which was measured in Supplementary Fig. 4b, where $K_{iT\text{-}C,\ 530nm} = k_{iTC,530nm} / k_{iCT,530nm} = [Cis]_{eq} / [Trans]_{eq}$. This can be calculated as $K_{iT\text{-}C,\ 530nm} = 94 / 6.0 = 15.67 \pm 1.57$.

Similar calculation was also performed for irradiation at 400 nm. The values of the rate constants for the isomerization of FAB were summarized in Supplementary Fig. 5g-h. These were used later for calculations and predictions of experiments in this work.

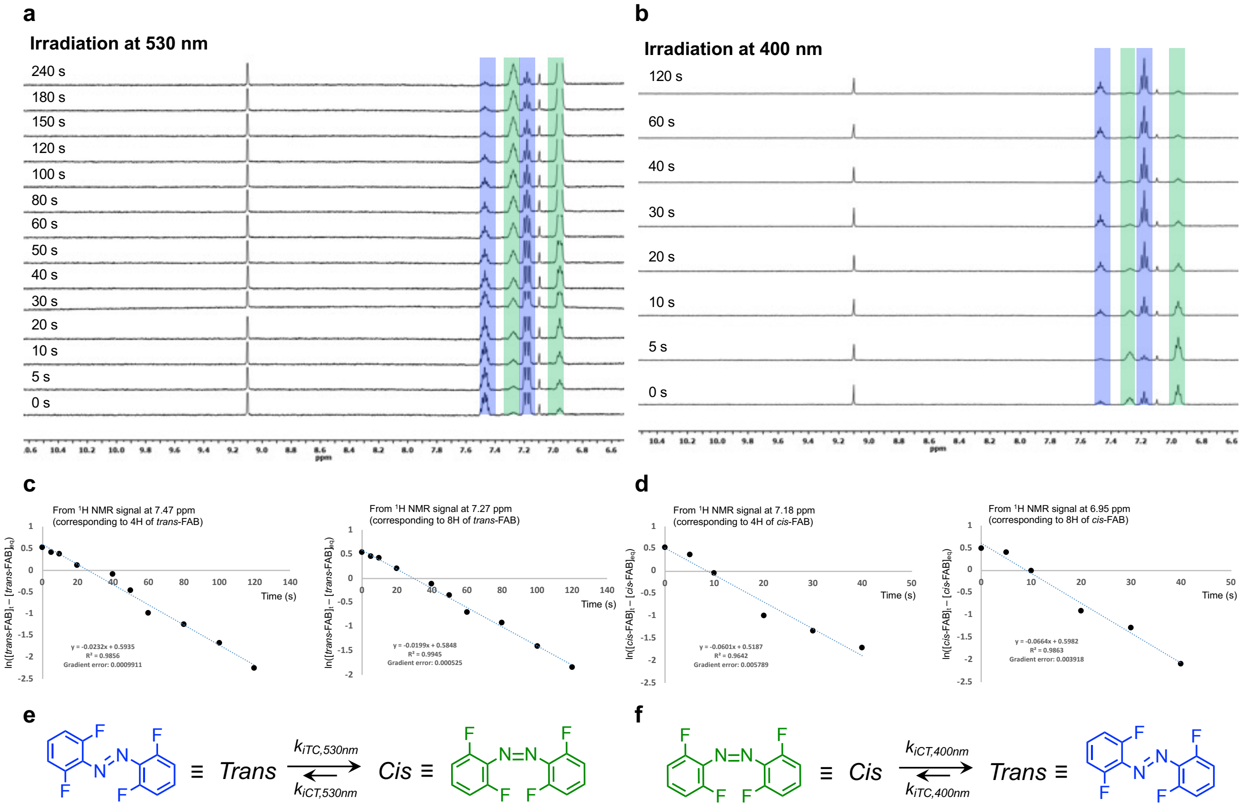

g

Isomerization from *trans*- to *cis*-FAB (green light – 530 nm)	
Average gradients of the graphs from **C** (s^{-1})	- 0.02155 ± 0.00076
Equilibrium constant: $K_{iT\text{-}C,\ 530\ nm}$	15.67 ± 1.57
**$k_{iCT,\ 530nm}$ (day^{-1})**	**111.7 ± 15.1**
**$k_{iTC,\ 530nm}$ (day^{-1})**	**1750 ± 236**

h

Isomerization from *cis*- to *trans*-FAB (blue light – 400 nm)	
Average gradients of the graphs from **D** (s^{-1})	- 0.06325 ± 0.00486
Equilibrium constant: $K_{iC\text{-}T,\ 400\ nm}$	11.50 ± 1.15
**$k_{iCT,\ 400nm}$ (day^{-1})**	**5028 ± 889**
**$k_{iTC,\ 400nm}$ (day^{-1})**	**437.2 ± 77.3**

Supplementary Fig. 5. a-b, ^{1}H NMR (500 MHz, dodecane, locked in D_2O) spectra showing isomerizations of *trans* to *cis*-FAB under irradiation at 530 nm (**a**), and *cis*- to *trans*-FAB under irradiation at 400 nm (**b**). NMR signals corresponding to *cis*-FAB and *trans*-FAB are highlighted in green and blue respectively. Coronene (9.1 ppm) was used as an internal standard for the measurements. The NMR solutions were kept under irradiation and the measurements were taken at different times. The tubes were covered with aluminum foil as the tubes were taken away from the light sources for measurements to avoid exposure to lights other than the light sources. The experiment time was paused during the NMR measurements and continued after the tubes were put back to the light sources. **c-d**, the plots showing the linear relationships for isomerization experiments with 530 nm (**c**) and 400 nm (**d**) light sources, as described by Eq. 2. Concentrations of corresponding FAB species were calculated from NMR measurements as shown in **a** and **b**. The gradients of the graphs were used further for calculation of the isomerization rate constants for *trans*- to *cis*-FAB (k_{iTC}) and *cis*- to *trans*-FAB (k_{iCT}). **e-f**, Chemical reactions for isomerization under irradiation at 530 nm (**e**) and 400 nm (**f**). **g-h**, Tables showing the values with errors for the average gradients of the graphs from part **c-d**, the isomerization equilibrium constants calculated from Supplementary Fig. 4b, and the rate constant k_{iTC} and k_{iCT} for isomerization processes under irradiation at 530 nm (**g**) and 400 nm (**h**).

3. ***Trans*- and *cis*-FAB encapsulation by cage 1 in water**

***Trans*-FAB ⊂ 1:** To a solution of cage **1** (2 mM, 0.4 mL) in D_2O, *trans*-FAB (0.7 mg, 2.8 μmol, 3.5 equiv.) was added. The sample was equilibrated for 24 hours at room temperature. ^1H NMR and ^1H DOSY NMR of the sample were measured to characterise the *trans*-FAB ⊂ **1** host-guest complex. The *trans*-FAB ⊂ **1** signals were observed at 6.4 ppm. The host signals were also observed to be shifted.

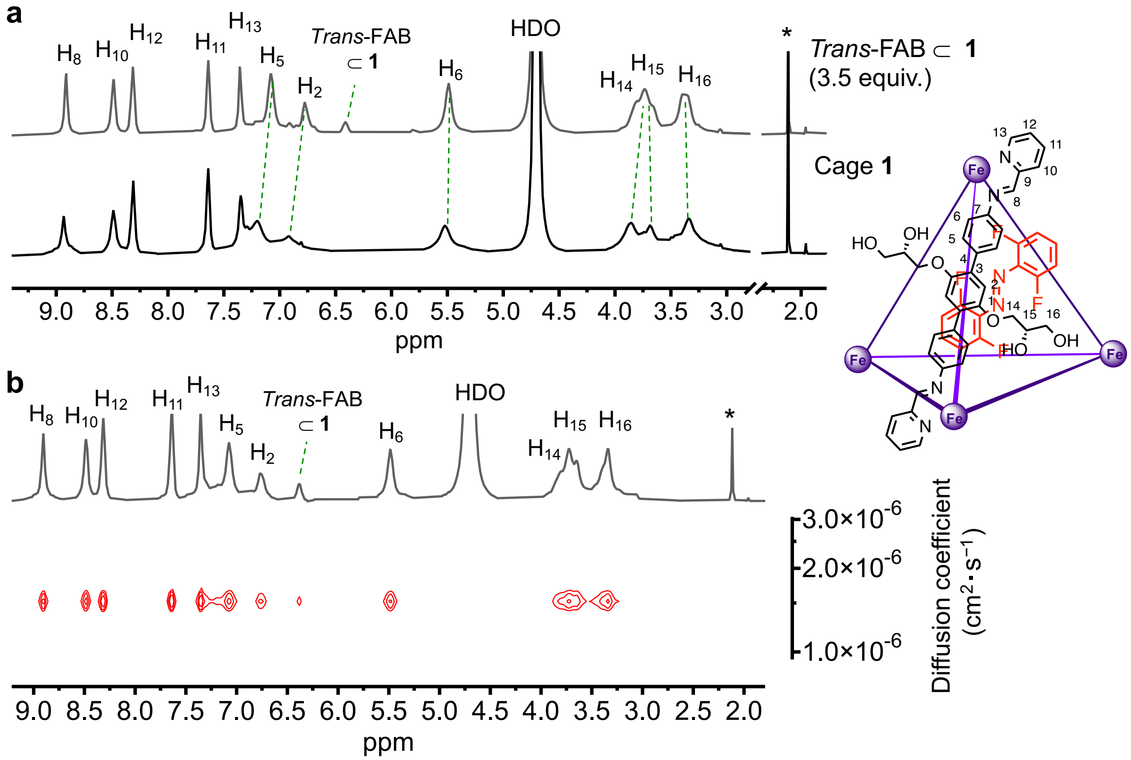

Supplementary Fig. 6. a, ^1H NMR (500 MHz, D_2O) spectra of cage **1** and the *trans*-FAB ⊂ **1** host-guest complex. (* = CH_3CN referencing signal). **b**, ^1H DOSY NMR (500 MHz, D_2O) spectrum of *trans*-FAB ⊂ **1**, suggesting that the cage and the encapsulated *trans*-FAB diffused together as a single entity. Because *trans*-FAB was poorly soluble in water, the excess unbound guest signals were not observed in the spectra.

***Cis*-FAB ⊂ 1:** To a solution of cage **1** (2 mM, 0.4 mL), *cis*-FAB (0.5 mg, 1.8 µmol, 2.3 equiv.) was added. After equilibration for 24 hours, ^{1}H NMR and ^{1}H DOSY NMR of the sample were measured to characterise the *cis*-FAB ⊂ **1** host-guest complex. The *cis*-FAB ⊂ **1** signals were observed at 6.3 and 5.8 ppm.

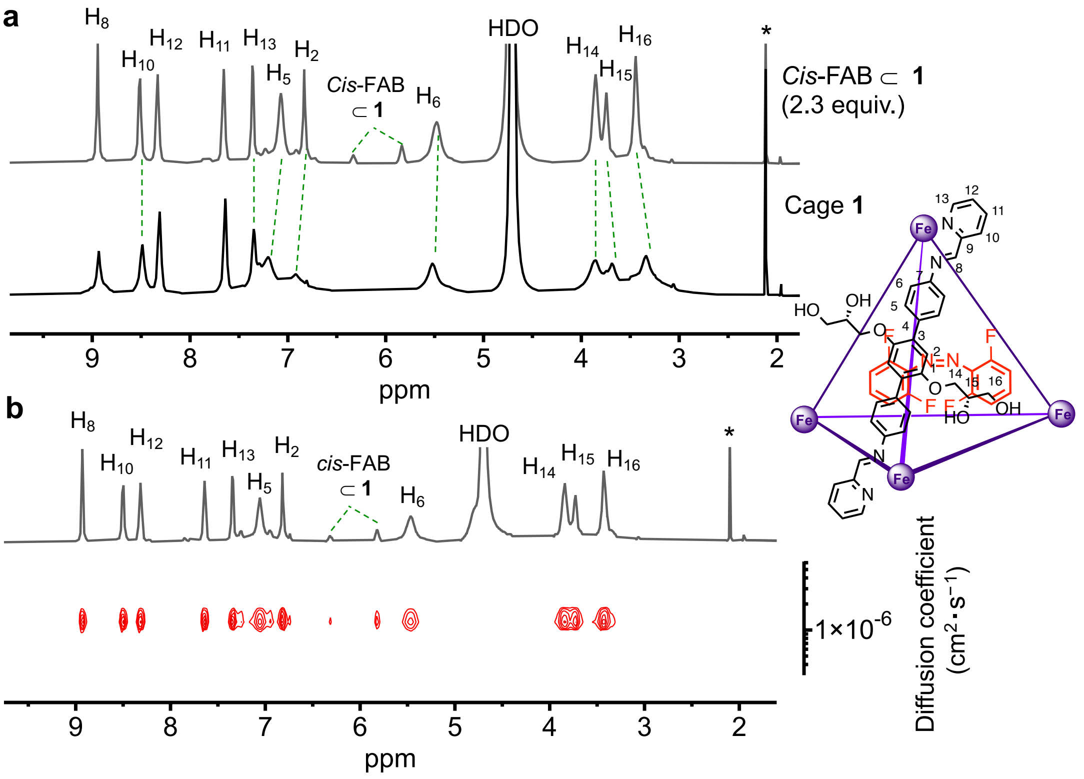

Supplementary Fig. 7. a, ^{1}H NMR (500 MHz, D_2O) spectra of cage **1** and the *cis*-FAB ⊂ **1** host-guest complex. (* = CH_3CN reference signal). **b**, ^{1}H DOSY NMR (500 MHz, D_2O) spectrum of *cis*-FAB ⊂ **1**, suggesting that the cage and the encapsulated *cis*-FAB diffused together as a single entity. The unbound guest signals were not observed in the spectra due to the low solubility of *cis*-FAB in water.

4. Naphthalene, *trans*- and *cis*-FAB hierarchy of encapsulation by 1 in water

In order to evaluate the relative binding affinities of naphthalene and the FAB isomers to cage **1**, we conducted an experiment to monitor the replacement of weakly-binding guests by strongly-binding ones. Cage **1** solution in D_2O (2 mM, 0.4 mL) was charged into an NMR tube. *Trans*-FAB (3.5 equiv.) was added and the system was left to equilibrate for 1 day. Guest encapsulation was studied by ^{1}H NMR experiments. To the same sample, *cis*-FAB (2 equiv.) was added, replacing the bound *trans*-FAB from **1** as the *cis*-FAB ⊂ **1** signals were observed instead of the *trans*-FAB ⊂ **1** ones. After naphthalene (5 equiv.) was added to the sample and the mixture was left equilibrating for 1 hour, the naphthalene ⊂ **1** signals were observed, suggesting that naphthalene had replaced the encapsulated *cis*-FAB. The relative binding strength of the guests to **1** is *trans*-FAB, *cis*-FAB and naphthalene in the order of increasing affinities.

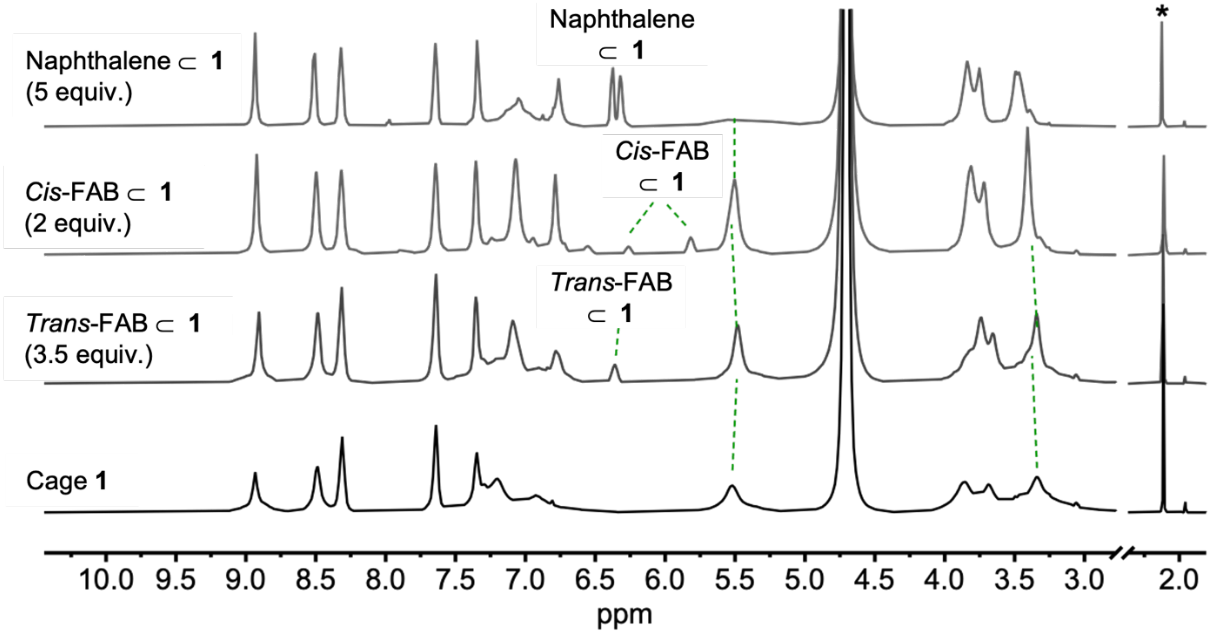

Supplementary Fig. 8. ^{1}H NMR (500 MHz, D_2O) monitoring of the relative binding strength of *trans*- and *cis*-FAB and naphthalene to cage **1** in water. *Trans*-FAB was first introduced to the cage solution and guest encapsulation was subsequently observed. Upon *cis*-FAB addition, the *trans*-FAB ⊂ **1** signals disappeared while the *cis*-FAB ⊂ **1** signals were observed. Addition of naphthalene displaced the encapsulated *cis*-FAB from cage **1**. (* = CH_3CN reference signal). *This figure is also presented in the Extended Data section as Extended Data Fig. 1.*

5. Quantitative analysis by ^{1}H NMR and uncertainty calculation

^{1}H NMR was used to monitor the concentration of compounds in the two arms of the U-tubes.

To conduct NMR measurements, a sample of 300 μL was taken from each of the two arms at each collection time point. The solutions were placed into NMR tubes (outer diameter = 5.0 mm, wall thickness 0.43 mm, length = 180 mm). Sealed capillary tubes (outer diameter = 1.8 – 2.0 mm, wall thickness = 0.28 – 0.32 mm, length = 100 mm) containing D_2O was inserted to the NMR tubes so that the experiments were locked with D_2O. After the measurement, the samples were used for UV-Vis measurements and re-injected back to the tubes for the continuing experiments. The solutions taken out were covered in aluminium foil to avoid external light at all times. The time used for every measurement was minimized (<30 minutes), to avoid disturbing the experiment.

All the ^{1}H NMR spectra used for quantitative studies were referenced to coronene as the internal standard, at 9.1 ppm. The coronene integral was measured from 9.15–9.05 ppm and was normalized to 100, corresponding to 12 protons.

To calculate uncertainty from NMR integration for each species following each measurement, two types of error are combined:

1) Systematic and processing error - this error is justified to ±5% of the integral for each signal,[3] deriving from relaxation, anodization, phasing, and baseline correction.

   To identify the systematic error of the measurements, the standard deviation of the absolute integrations of the coronene internal standard (0.25 mM) from all measurements was inferred to be ±7% of the average absolute integration value (n = 22). This standard deviation corresponds to the total uncertainty of the coronene internal standard peak integral for each measurement. The systematic error of the measurements is the standard deviation less the noise level (±2%, see below), which is ±5% of the integral for each signal.

2) Noise level – this is inferred to be ±2% of the integral of the coronene internal standard peak, corresponding to ±2 in absolute value, since the coronene integral was normalized to 100 for every spectrum. The noise level was calculated from the signal-to-noise ratio (SNR) with respect to the coronene internal standard peak. The SNR of coronene (0.25 mM) was found to be 110, measured by using the SNR Calculation script function in Mnova, taking the average of multiple spectra (n = 22), which corresponds to approximately ±2% uncertainty in the integral.[4]

$$\frac{\partial \int \text{coronene(noise)}}{\int \text{coronene}} = 0.02\,.$$

   For every spectrum, $\int$coronene is normalized to 100 during processing. Therefore, the noise level, $\Delta p = \partial \int \text{coronene(noise)} = 2$.

Since the integral width for each relevant peak in this work is comparable (~ 0.1 ppm), the absolute uncertainty of all peak integrations in the work is also the same; i.e., $\partial\int\text{peak(noise)} = \Delta\text{p} = 2$.

Combining two sources of uncertainties: 1) the systematic and processing error (±5% of each peak integration) and 2) the noise level (±2% of the integral of the coronene internal standard), we have calculated the total integration uncertainty for each peak:

$$\partial\int\text{peak(total)} = \partial\int\text{peak(systematic)} + \partial\int\text{peak(noise)} = (5\% \times \int\text{peak}) + \Delta\text{p}$$

$$\partial\int\text{peak(total)} = (0.05 \times \int\text{peak}) + (2) \qquad \textbf{(Eq. 3)}$$

where $\int\text{peak} =$ the normalised integral of a signal in the measurement (setting $\int\text{coronene} = 100$)

For example, $\partial\int\text{coronene} = (0.05 \times \int\text{coronene}) + 2 = 7$.

It is worth mentioning that the uncertainty from the noise contribution, $\partial\int\text{peak(noise)}$, is independent of the peak integral, meaning that it is most significant for the integration of the lower concentration peaks. At higher concentration, the total uncertainty is dominated by the systematic error of the NMR measurement.

FAB concentration and uncertainty

Trans-FAB signals were measured over a range of 7.52–7.42 ppm, corresponding to $2H_{j\text{-}trans}$, and 7.22–7.14 ppm, corresponding to $4H_{k\text{-}trans}$. *Cis*-FAB signals were measured over a range of 7.32–7.22 ppm, corresponding to $2H_{j\text{-}cis}$, and 7.00–6.90 ppm, corresponding to $4H_{k\text{-}cis}$. The concentrations of *trans*- and *cis*-FAB were calculated using Eq. 4, referencing to coronene internal standard (0.25 mM).

$$[trans/cis\text{-FAB}] = \frac{\text{F}_{trans/cis} \times 0.25\text{ mM} \times 12}{\int\text{coronene}}\ ; \qquad \textbf{(Eq. 4)}$$

$$\text{where } \text{F}_{trans/cis} = \frac{\int\text{H}_{\text{j}-trans/cis} + \int\text{H}_{\text{k}-trans/cis}}{6}$$

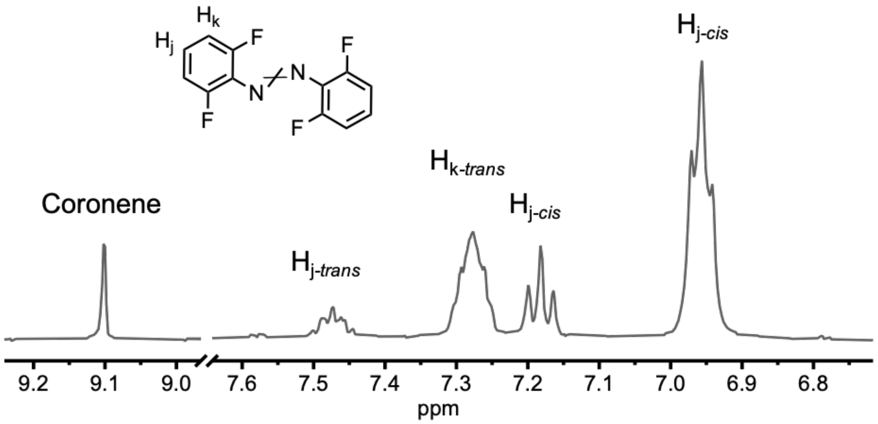

Supplementary Fig. 9. ^{1}H NMR spectrum (500 MHz, D_2O, 298 K) of a sample from the receiving arm containing FAB and coronene. The integrals of the *trans*- and *cis*-FAB peak signals were measured referenced to coronene at 9.1 ppm, the integral of which was normalized to 100.

The error propagation in *trans*- and *cis*-FAB concentration is calculated using Eq. 5 and 6. The derivation for uncertainty in [*trans*-FAB] (Eq. 5), taking into account the error propagation in *trans*-FAB concentration is provided below.

$$\partial[\textit{trans}\text{-FAB}] = \partial\left(\frac{\mathrm{F}_{trans} \times 0.25\ \mathrm{mM} \times 12}{\int \mathrm{coronene}}\right) = 3 \times \partial\left(\frac{\mathrm{F}_{trans}}{\int \mathrm{coronene}}\right)$$

in which:

$$\partial\left(\frac{\mathrm{F}_{trans}}{\int \mathrm{coronene}}\right) = \frac{\mathrm{F}_{trans}}{\int \mathrm{coronene}} \times \sqrt{\left(\frac{\partial \mathrm{F}_{trans}}{\mathrm{F}_{trans}}\right)^2 + \left(\frac{\partial \int \mathrm{coronene}}{\int \mathrm{coronene}}\right)^2}.$$

Therefore:

$$\partial[\textit{trans}\text{-FAB}] = 3 \times \frac{\mathrm{F}_{trans}}{\int \mathrm{coronene}} \times \sqrt{\left(\frac{\partial \mathrm{F}_{trans}}{\mathrm{F}_{trans}}\right)^2 + \left(\frac{\partial \int \mathrm{coronene}}{\int \mathrm{coronene}}\right)^2},$$

where

$$\partial \mathrm{F}_{trans} = \frac{1}{6} \times \sqrt{\left(\partial \mathrm{H}_{\mathrm{j}-trans}\right)^2 + (\partial \mathrm{H}_{\mathrm{k}-trans})^2}.$$

$\partial \mathrm{H}_{\mathrm{j}-trans}$ and $\partial \mathrm{H}_{\mathrm{k}-trans}$ are calculated according to Eq. 3:

$$\partial \mathrm{H}_{\mathrm{j}-trans} = (0.05 \times \int \mathrm{H}_{\mathrm{j}-trans}) + 2,$$

$$\partial \mathrm{H}_{\mathrm{k}-trans} = (0.05 \times \int \mathrm{H}_{\mathrm{k}-trans}) + 2.$$

As coronene internal standard integration is normalized to 100, ∫coronene = 100, and from Eq. 3, $\partial\int$coronene = (0.05 × ∫coronene) + 2 = 7. Hence,

$$\partial[trans\text{-FAB}] = 3 \times \frac{F_{trans}}{100} \times \sqrt{\left(\frac{\partial F_{trans}}{F_{trans}}\right)^2 + (0.07)^2} \quad \textbf{(Eq. 5)}$$

And similarly,

$$\partial[cis\text{-FAB}] = 3 \times \frac{F_{cis}}{100} \times \sqrt{\left(\frac{\partial F_{cis}}{F_{cis}}\right)^2 + (0.07)^2} \quad \textbf{(Eq. 6)}$$

Naphthalene concentration and uncertainty

Naphthalene signals were measured over a range of 8.06–7.95 ppm, corresponding to $4H_a$, and 7.68–7.58 ppm, corresponding to $4H_b$. The concentration of naphthalene was calculated using Eq. 7.

$$[\text{Naphthalene}] = \frac{\text{N} \times 0.25\ \text{mM} \times 12}{\int\text{coronene} \times 4} \quad \textbf{(Eq. 7)}$$

where N is the average of the integrals of H_a and H_b, corresponding to 4 protons.

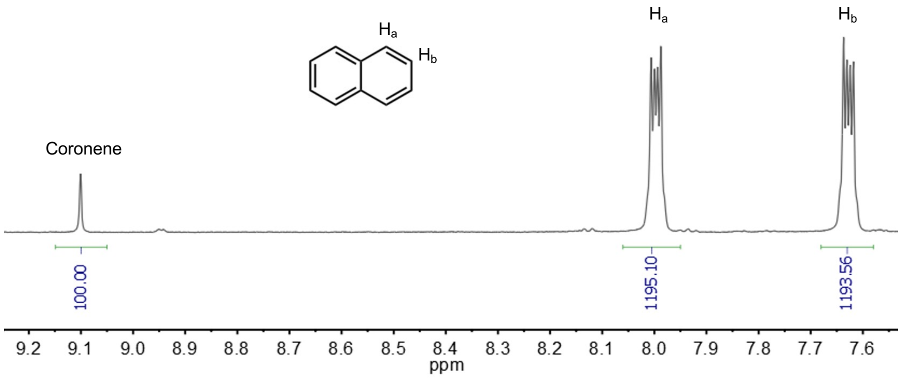

Supplementary Fig. 10. ^{1}H NMR spectrum (500 MHz, D_2O, 298 K) of a sample from the receiving arm containing naphthalene and coronene. The integrals for the naphthalene signals were referenced to coronene at 9.1 ppm, the integral of which was normalized to 100.

The derivation for uncertainty in [Naphthalene] (Eq. 8), corresponding to the error propagation in naphthalene concentration is provided below.

$$\partial[\text{Naphthalene}] = \partial\left(\frac{\text{N} \times 0.25\ \text{mM} \times 12}{\int\text{coronene} \times 4}\right) = \frac{3}{4} \times \partial\left(\frac{\text{N}}{\int\text{coronene}}\right)$$

in which:

$$\partial\left(\frac{\text{N}}{\int\text{coronene}}\right) = \frac{\text{N}}{\int\text{coronene}} \times \sqrt{\left(\frac{\partial\text{N}}{\text{N}}\right)^2 + \left(\frac{\partial\int\text{coronene}}{\int\text{coronene}}\right)^2},$$

where

$$\partial\text{N} = \frac{1}{2} \times \sqrt{(\partial\text{H}_\text{a})^2 + (\partial\text{H}_\text{b})^2}.$$

$\partial\text{H}_\text{a}$ and $\partial\text{H}_\text{b}$ are calculated according to Eq. 3:

$$\partial\text{H}_\text{a} = (0.05 \times \int\text{H}_\text{a}) + 2,$$

$$\partial\text{H}_\text{b} = (0.05 \times \int\text{H}_\text{b}) + 2.$$

As the integral of the coronene internal standard is normalized to 100, $\int$coronene = 100, and from Eq. 3, $\partial\int\text{coronene} = (0.05 \times \int\text{coronene}) + 2 = 7$. Hence,

$$\partial[\text{Naphthalene}] = \frac{3}{4} \times \frac{\text{N}}{100} \times \sqrt{\left(\frac{\partial\text{N}}{\text{N}}\right)^2 + (0.07)^2} \qquad \textbf{(Eq. 8)}$$

6. FAB UV-Vis absorption: calibration and peak fitting

The UV-Vis absorption spectra of the solutions in arms I and II were also measured. As suggested by the NMR results in System 1 (Supplementary Section 7), there was a significant amount of *trans*-FAB presented together with *cis*-FAB in the first two days. As a result, the absorption signal in arm I in the first two days was the sum of *trans*- and *cis*-FAB. The absorption band in the range of 350 – 600 nm was deconvoluted using LogNormal equations[3] to obtain the overlapping *trans*- and *cis*-FAB spectra. The fitting was performed using Origin Software (OriginPro 2020 SR1, version 9.7.0.188 (Academic)). After 2 days of irradiation at 530 nm, there was only a small trace of *trans*-FAB remaining as suggested by ^{1}H NMR results. The absorption maximum at 415 nm of the subsequently collected spectra was assigned to *cis*-FAB. Similarly, there was only a negligible amount of *cis*-FAB present in arm II. The absorption maximum at 465 nm was therefore attributed to *trans*-FAB.

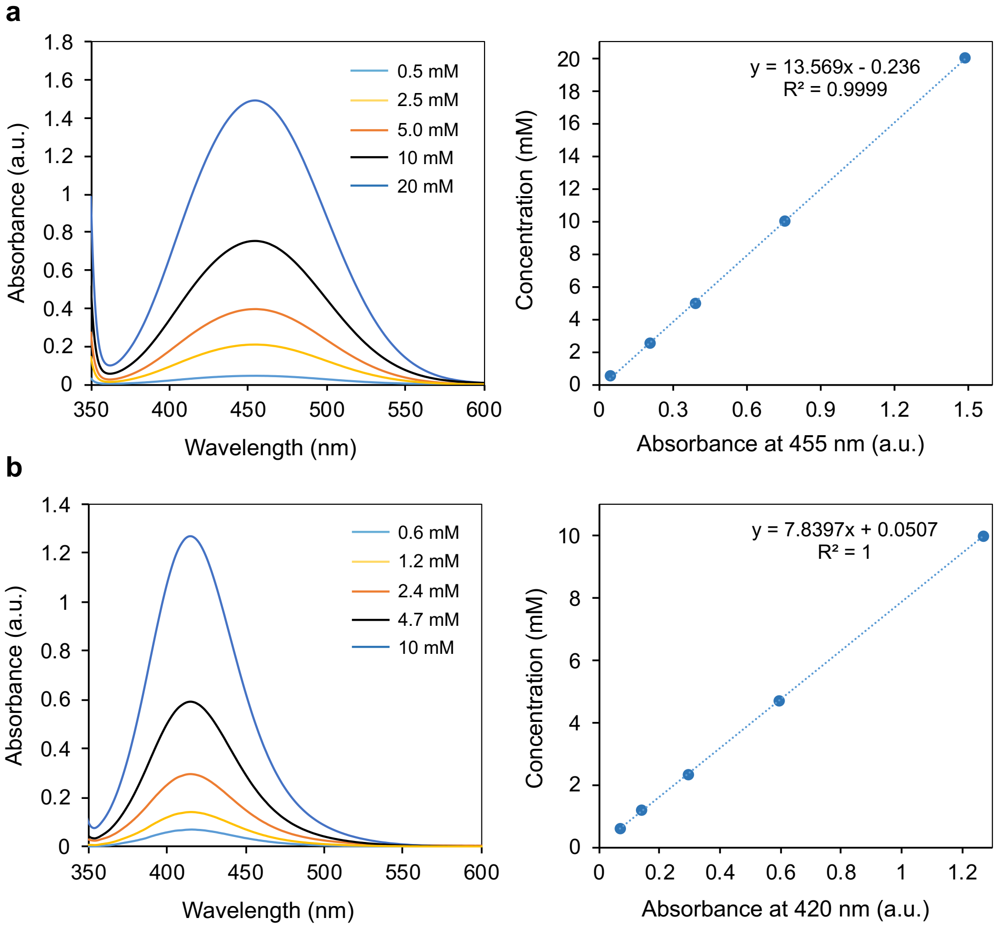

Supplementary Fig. 11. Calibration lines for **a**, *trans*- and **b**, *cis*-FAB concentration as a function of UV-Vis absorbance. The calibration lines were obtained from the samples after irradiations during 2 days, where the trace amount of the other FAB isomer present was treated as negligible.

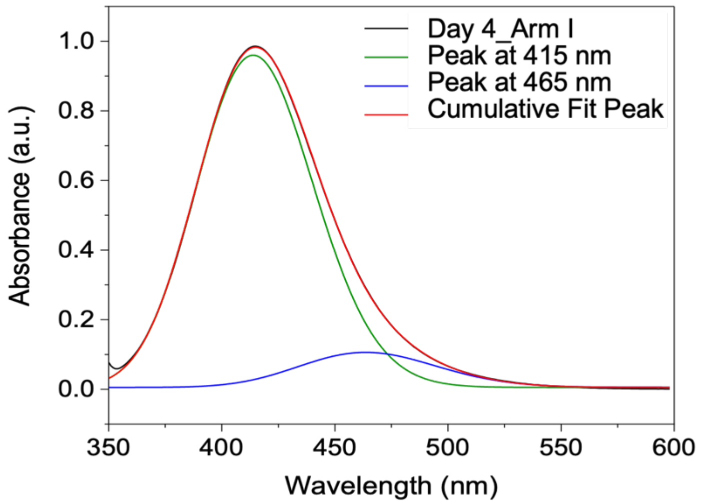

Supplementary Fig. 12. Example of the deconvolution of an arm I absorption curve using LogNormal equation. The fitted peaks were set to 465 and 415 nm corresponding to the wavelength of the absorption maximum of *trans*-FAB and *cis*-FAB, respectively. The fitting was performed using Origin.

Unlike NMR, the UV measurement cannot provide signals uniquely for each FAB isomer without data processing by deconvolution. Thus, the model fitting to the experimental results for this project was performed on the NMR experimental results for better quantitative analysis. UV-Vis experiments were carried out to ensure similar trend was observed for each experiment qualitatively.

7. Maxwell's Demon experiment (System 1): experimental results

In System 1, the U-tube was prepared by adding an aqueous solution of cage **1** (4 mM, 2.5 mL, 25 mol% relative to the total FAB in both arms) into the bottom of the U-tube (internal diameter 1.2 cm), and aliquots of a dodecane solution containing FAB (10 mM, 2 mL, 90% *trans*) into each arm. The dodecane solutions contained coronene (0.25 mM) as an internal standard. In addition, arm II contained triisopropylbenzene (10 mM) as an indicator. During the experiment, the cage layer was stirred at 250 rpm at room temperature with a cylindrical magnetic stir bar (3 x 6 mm). Over the first 24 days, arm I was irradiated at 530 nm with a LED light strip, and arm II at 400 nm. After 24 days, the LED light strips were exchanged, where arm I was irradiated at 400 nm, and arm II at 530 nm.

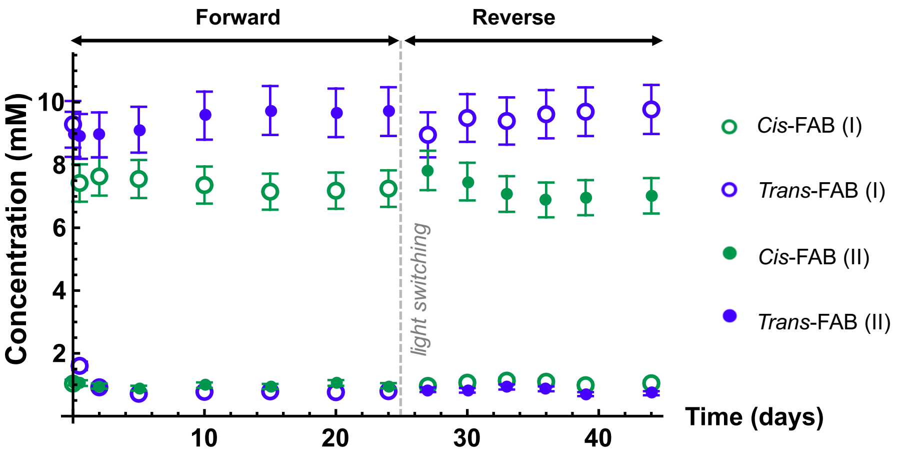

Supplementary Fig. 13. Graph showing experimental results measured by ^{1}H NMR referencing to coronene (0.25 mM) of *cis*- and *trans*-FAB in arm I and arm II solutions of System 1. Arm I was irradiated at 530 nm and 400 nm during the forward and reverse transport, respectively. Arm II was irradiated at 400 nm and 530 nm during the forward and reverse transport, respectively. The grey dashed line indicates the point which the LED light strips were swapped, switching from forward to reverse transport. Data are presented as mean values +/- measurement errors, derived from error propagation of the SD and the signal-to-noise ratio (n=22) of coronene (Supplementary Section 5).

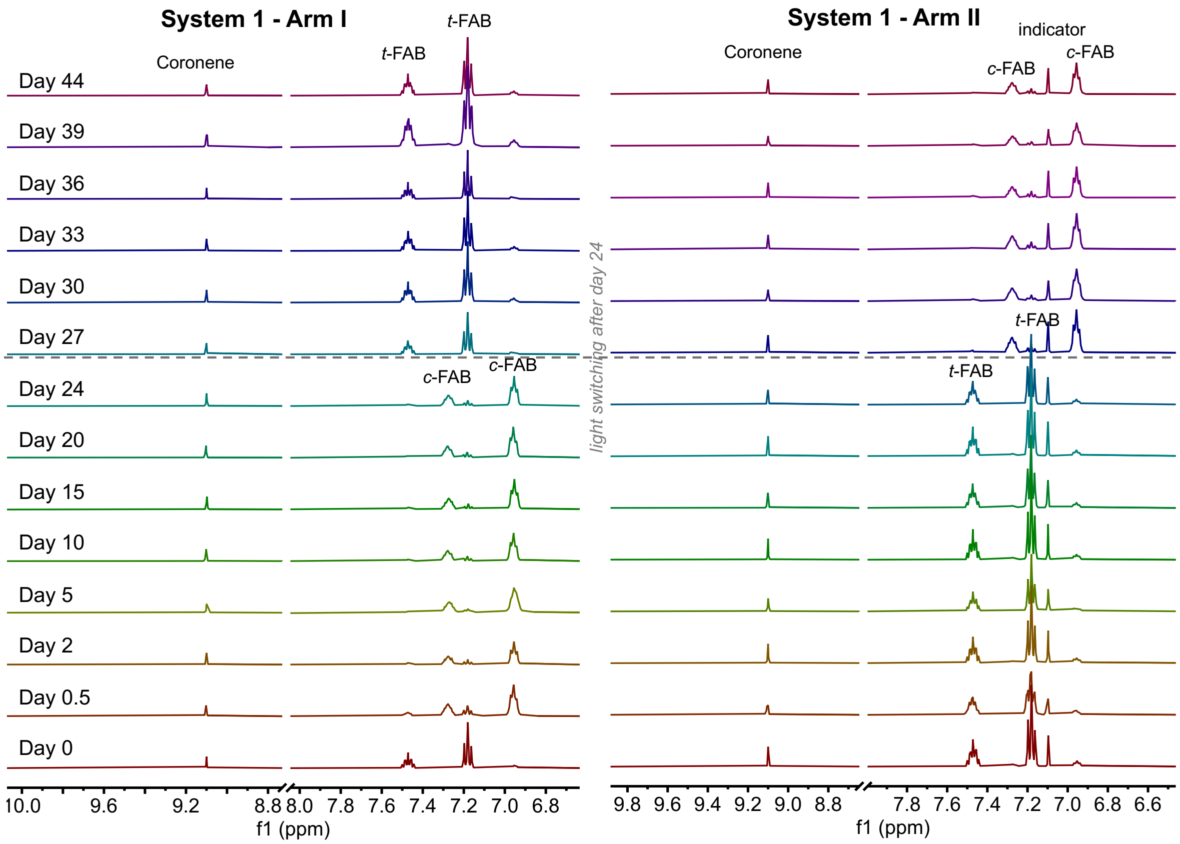

Supplementary Fig. 14. ^{1}H NMR spectra (500 MHz, D_2O, 298 K) of the measurements from System 1 at different times, showing the peaks for coronene, *trans*-FAB (*t*-FAB) and *cis*-FAB (*c*-FAB). The spectra on the left are for the samples taken from arm I and the spectra on the right are for the samples taken from arm II. The integrals of *trans*- and *cis*-FAB peak signals were measured referencing to coronene at 9.1 ppm which integral was normalized to 100. Arm I was irradiated at 530 nm and 400 nm during the forward and reverse transport, respectively. Arm II was irradiated at 400 nm and 530 nm during the forward and reverse transport, respectively. The grey dashed line indicates the point which the LED light strips were swapped, switching from forward to reverse transport.

Supplementary Table 1. Raw data from NMR integration, uncertainty and *trans*- and *cis*-FAB concentration values in arms I and II of System 1 measured by ^{1}H NMR referenced to coronene (0.25 mM). The green background color illustrates the period when the arms were irradiated at 530 nm, while the purple background color represents irradiation at 400 nm. The data are also included as a separate excel file as the Source Data for Supplementary Figures.

System 1	Maxwell's Demon Experiment	
IS	0.25 mM coronene (normalized to 100)	
Error	Systematic	5%
	Noise level	2

System 1

Arm I

Arm I	Time (day)	cis-FAB									trans-FAB									FAB (total)		
		∫Hj-cis	∂Hj-cis	∫Hk-cis	∂Hk-cis	Fcis	∂Fcis	**[cis-FAB]**	∂[cis-FAB]	%∂[cis-FAB]	∫Hj-trans	∂Hj-trans	∫Hk-trans	∂Hk-trans	Ftrans	∂Ftrans	**[trans-FAB]**	∂[trans-FAB]	%∂[trans-FAB]	**[FAB] (I)**	∂[FAB] (I)	%∂[FAB] (I)
Forward transport (530 nm)	**0**	68.45	5.42	138.70	8.94	34.53	1.74	**1.04**	0.09	8.63	620.83	33.04	1238.14	63.91	309.83	11.99	**9.29**	0.74	8.00	**10.33**	0.75	7.25
	**0.5**	478.68	25.93	1006.58	52.33	247.54	9.73	**7.43**	0.60	8.03	106.98	7.35	213.50	12.68	53.41	2.44	**1.60**	0.13	8.36	**9.03**	0.61	6.77
	**2**	506.31	27.32	1021.09	53.05	254.57	9.95	**7.64**	0.61	8.02	65.90	5.30	117.76	7.89	30.61	1.58	**0.92**	0.08	8.70	**8.56**	0.62	7.22
	**5**	494.27	26.71	1016.43	52.82	251.78	9.87	**7.55**	0.61	8.02	47.88	4.39	95.02	6.75	23.82	1.34	**0.71**	0.06	8.99	**8.27**	0.61	7.37
	**10**	490.20	26.51	981.86	51.09	245.34	9.59	**7.36**	0.59	8.02	50.74	4.54	104.24	7.21	25.83	1.42	**0.77**	0.07	8.90	**8.14**	0.59	7.30
	**15**	472.57	25.63	957.47	49.87	238.34	9.35	**7.15**	0.57	8.02	53.63	4.68	104.41	7.22	26.34	1.43	**0.79**	0.07	8.87	**7.94**	0.58	7.28
	**20**	466.62	25.33	969.80	50.49	239.40	9.41	**7.18**	0.58	8.03	50.42	4.52	102.53	7.13	25.49	1.41	**0.76**	0.07	8.91	**7.95**	0.58	7.31
	**24**	477.45	25.87	972.10	50.61	241.59	9.47	**7.25**	0.58	8.02	53.56	4.68	105.66	7.28	26.54	1.44	**0.80**	0.07	8.86	**8.04**	0.59	7.28
Reverse transport (400 nm)	**27**	54.31	4.72	138.12	8.91	32.07	1.68	**0.96**	0.08	8.74	602.59	32.13	1189.60	61.48	298.70	11.56	**8.96**	0.72	8.00	**9.92**	0.72	7.27
	**30**	62.43	5.12	150.70	9.54	35.52	1.80	**1.07**	0.09	8.65	631.79	33.59	1267.58	65.38	316.56	12.25	**9.50**	0.76	8.00	**10.56**	0.77	7.24
	**33**	66.69	5.33	159.52	9.98	37.70	1.89	**1.13**	0.10	8.60	625.40	33.27	1254.81	64.74	313.37	12.13	**9.40**	0.75	8.00	**10.53**	0.76	7.20
	**36**	63.22	5.16	155.78	9.79	36.50	1.84	**1.10**	0.09	8.63	661.10	35.06	1262.37	65.12	320.58	12.33	**9.62**	0.77	7.99	**10.71**	0.77	7.22
	**39**	48.77	4.44	148.27	9.41	32.84	1.73	**0.99**	0.09	8.77	656.90	34.85	1282.59	66.13	323.25	12.46	**9.70**	0.77	7.99	**10.68**	0.78	7.30
	**44**	60.03	5.00	149.34	9.47	34.90	1.78	**1.05**	0.09	8.67	654.30	34.72	1299.50	66.98	325.63	12.57	**9.77**	0.78	7.99	**10.82**	0.79	7.27

Arm II

Arm II	Time (day)	cis-FAB									trans-FAB									FAB (total)		
		∫Hj-cis	∂Hj-cis	∫Hk-cis	∂Hk-cis	Fcis	∂Fcis	**[cis-FAB]**	∂[cis-FAB]	%∂[cis-FAB]	∫Hj-trans	∂Hj-trans	∫Hk-trans	∂Hk-trans	Ftrans	∂Ftrans	**[trans-FAB]**	∂[trans-FAB]	%∂[trans-FAB]	**[FAB] (II)**	∂[FAB] (II)	%∂[FAB] (II)
Forward transport (400 nm)	**0**	70.54	5.53	143.93	9.20	35.75	1.79	**1.07**	0.09	8.60	598.47	31.92	1196.74	61.84	299.20	11.60	**8.98**	0.72	8.00	**10.05**	0.72	7.21
	**0.5**	65.63	5.28	144.96	9.25	35.10	1.77	**1.05**	0.09	8.64	595.52	31.78	1186.53	61.33	297.01	11.51	**8.91**	0.71	8.00	**9.96**	0.72	7.21
	**2**	58.92	4.95	132.80	8.64	31.95	1.66	**0.96**	0.08	8.72	594.17	31.71	1197.25	61.86	298.57	11.59	**8.96**	0.72	8.00	**9.92**	0.72	7.28
	**5**	57.79	4.89	120.47	8.02	29.71	1.57	**0.89**	0.08	8.76	610.78	32.54	1213.96	62.70	304.12	11.77	**9.12**	0.73	8.00	**10.02**	0.73	7.33
	**10**	63.70	5.19	133.95	8.70	32.94	1.69	**0.99**	0.09	8.67	639.22	33.96	1275.19	65.76	319.07	12.34	**9.57**	0.77	8.00	**10.56**	0.77	7.29
	**15**	57.63	4.88	131.32	8.57	31.49	1.64	**0.94**	0.08	8.73	648.93	34.45	1298.31	66.92	324.54	12.54	**9.74**	0.78	8.00	**10.68**	0.78	7.33
	**20**	62.92	5.15	148.66	9.43	35.26	1.79	**1.06**	0.09	8.65	644.30	34.22	1287.94	66.40	322.04	12.45	**9.66**	0.77	8.00	**10.72**	0.78	7.26
	**24**	57.85	4.89	136.02	8.80	32.31	1.68	**0.97**	0.08	8.72	647.32	34.37	1292.95	66.65	323.38	12.50	**9.70**	0.78	8.00	**10.67**	0.78	7.31
Reverse transport (530 nm)	**27**	509.70	27.49	1055.53	54.78	260.87	10.21	**7.83**	0.63	8.02	61.87	5.09	106.50	7.33	28.06	1.49	**0.84**	0.07	8.78	**8.67**	0.63	7.29
	**30**	470.90	25.55	1023.36	53.17	249.04	9.83	**7.47**	0.60	8.04	57.57	4.88	106.82	7.34	27.40	1.47	**0.82**	0.07	8.82	**8.29**	0.60	7.29
	**33**	454.88	24.74	960.18	50.01	235.84	9.30	**7.08**	0.57	8.03	61.66	5.08	124.03	8.20	30.95	1.61	**0.93**	0.08	8.72	**8.00**	0.57	7.17
	**36**	442.62	24.13	934.92	48.75	229.59	9.07	**6.89**	0.55	8.04	64.33	5.22	109.49	7.47	28.97	1.52	**0.87**	0.08	8.75	**7.76**	0.56	7.20
	**39**	455.94	24.80	935.83	48.79	231.96	9.12	**6.96**	0.56	8.03	51.95	4.60	88.28	6.41	23.37	1.32	**0.70**	0.06	8.98	**7.66**	0.56	7.34
	**44**	455.79	24.79	947.87	49.39	233.94	9.21	**7.02**	0.56	8.03	60.09	5.00	85.88	6.29	24.33	1.34	**0.73**	0.07	8.91	**7.75**	0.57	7.32

The UV-Vis absorption of *cis*-FAB and *trans*-FAB in arms I and arm II were also measured, respectively, The isomer concentrations were calculated based on maximum absorption of *trans*-FAB (at 465 nm) and *cis*-FAB (at 415 nm) using linear fitting equations in Supplementary Fig. 11.

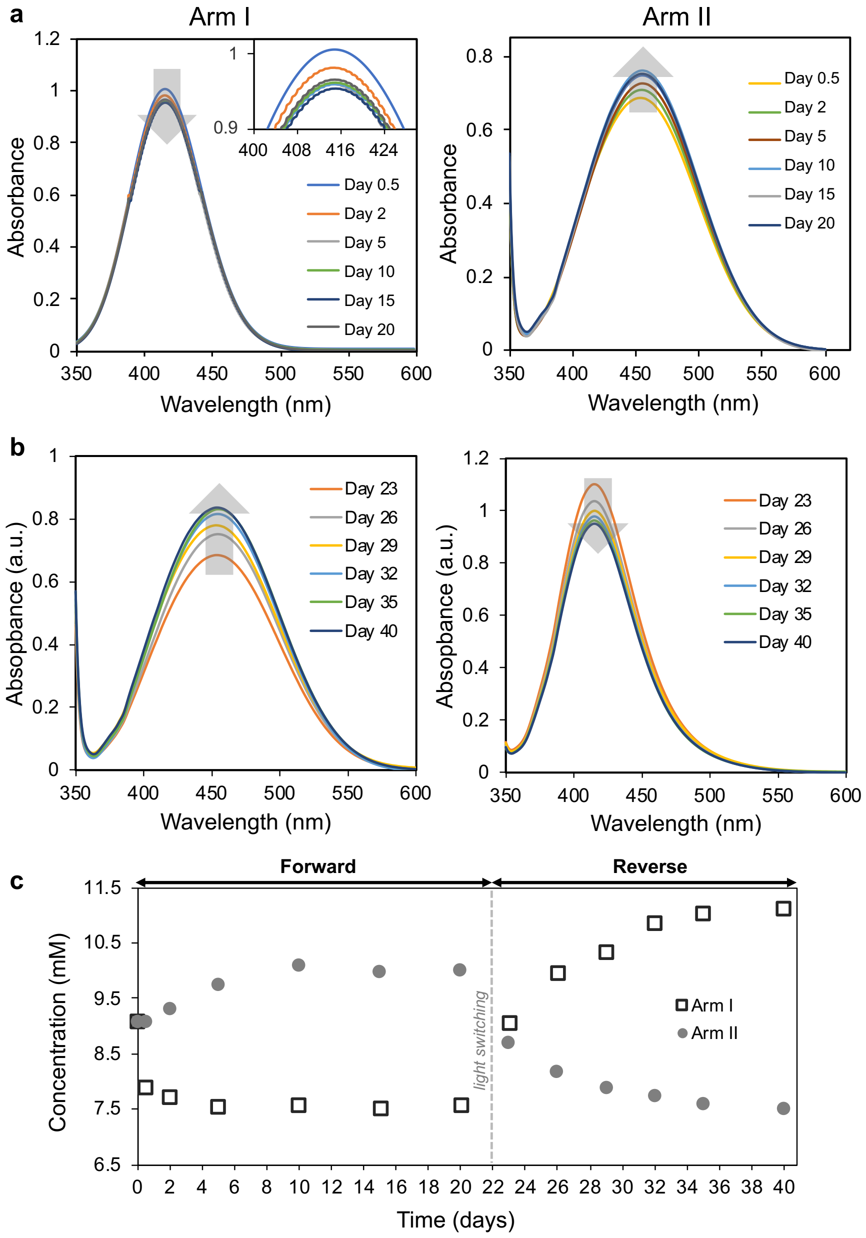

Supplementary Fig. 15. UV-Vis measurements for System 1. **a**, UV-Vis absorption of arm I and arm II solutions during the forward transport process. Arm I was irradiated at 400 nm to promote *trans*-FAB formation, and arm II was irradiating at 530 nm to obtain *cis*-FAB. While *trans*-FAB concentration was observed to accumulate in arm I, *cis*-FAB was depleted in arm II. **b**, UV-Vis absorption of arm I and arm II solutions during the reverse process with Arm I was irradiated at 530 nm and arm II was irradiating at 400 nm. **c**, Concentration of *trans*-FAB and *cis*-FAB in arm I and arm II were calculated using linear fitting equations for both forward and reversed processes. The grey dashed line indicates the point which the LED light strips were swapped, switching from

forward to reverse transport. Unlike NMR, the UV-Vis measurement does not provide unique signals for each FAB isomer without data processing by deconvolution. Thus, the quantification of our experimental results was performed using the NMR experimental results. UV-Vis experiments were performed as a qualitative check on each experiment.

Supplementary Table 2. Maximum absorbance from the UV measurements and FAB concentration values in arms I and II in System 1. Concentration of FAB in arm I and arm II were calculated using linear fitting equations to the maximum absorbance for both forward and reversed processes as shown in Supplementary Fig. 11. The green background illustrates the period when the arms were irradiated at 530 nm, while the purple background represents light irradiation at 400 nm. The data are also included as a separate excel file as the Source Data for Supplementary Figures

System 1 - UV								
Arm I					Arm II			
Arm I	Time (days)	Max Abs (a.u.)	Concentration (mM)		Arm II	Time (days)	Max Abs (a.u.)	Concentration (mM)
Forward transport (530 nm)	**0**	1.15	**9.09**	*light switching on day 20*	Forward transport (400 nm)	**0**	0.69	**9.09**
	**0.5**	1.00	**7.89**			**0.5**	0.69	**9.09**
	**2**	0.98	**7.73**			**2**	0.70	**9.30**
	**5**	0.96	**7.55**			**5**	0.74	**9.74**
	**10**	0.96	**7.58**			**10**	0.76	**10.08**
	**15**	0.95	**7.53**			**15**	0.75	**9.97**
	**20**	0.96	**7.58**			**20**	0.76	**10.01**
Reverse transport (400 nm)	**23**	0.68	**9.05**		Reverse transport (530 nm)	**23**	1.10	**8.69**
	**26**	0.75	**9.97**			**26**	1.04	**8.17**
	**29**	0.78	**10.35**			**29**	1.00	**7.88**
	**32**	0.82	**10.85**			**32**	0.98	**7.73**
	**35**	0.83	**11.04**			**35**	0.96	**7.60**
	**40**	0.84	**11.13**			**40**	0.95	**7.50**

8. Naphthalene Counterflow Augments Maxwell's Demon experiment (System 2): experimental results

In System 2, the U-tube was prepared by adding an aqueous solution of cage **1** (4 mM, 2.5 mL, 25 mol% relative to the total FAB in both arms) into the bottom of the U-tube (internal diameter 1.2 cm), and aliquots of a dodecane solution containing FAB (10 mM, 2 mL, 90% *trans*) into each arm. The solutions contained coronene (0.25 mM) as an internal standard. In addition, arm II contained triisopropylbenzene (10 mM) as an indicator. Naphthalene (11 mM) was also added to arm II. During the experiment, the cage layer was stirred at 250 rpm at room temperature with a cylindrical magnetic stir bar (3 x 6 mm). During the first 20 days, arm I was irradiated at 530 nm with a LED light strip, and arm II at 400 nm. After 20 days, the LED light strips were exchanged, where arm I was irradiated at 400 nm, and arm II at 530 nm.

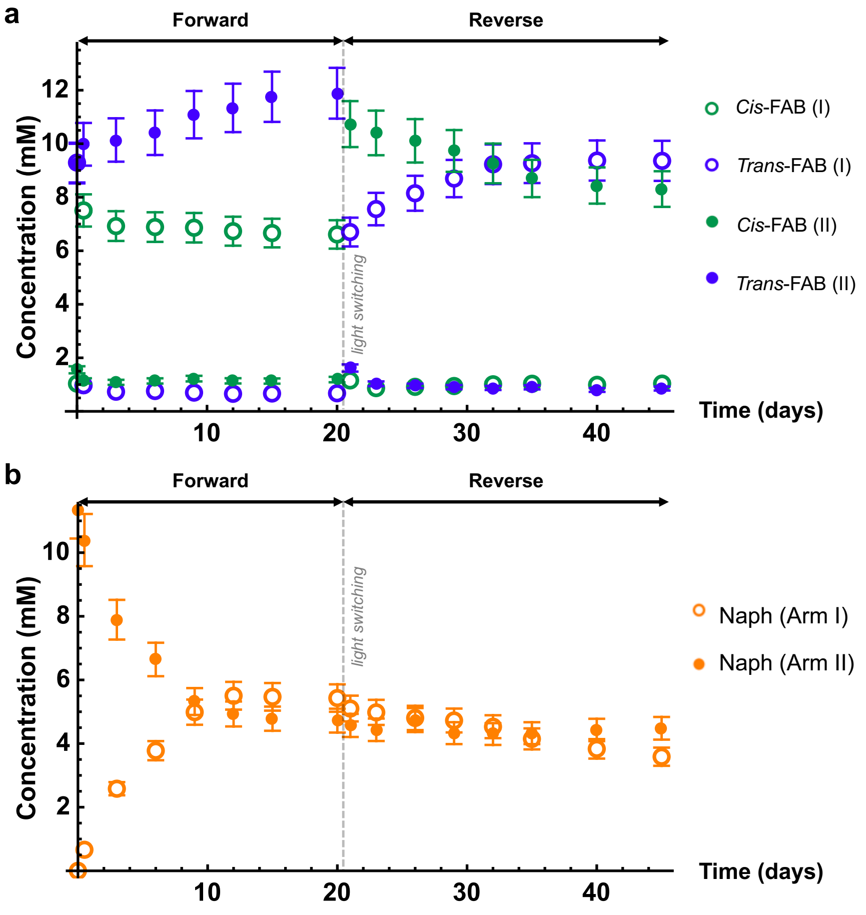

Supplementary Fig. 16. Graph showing experimental results measured by ^{1}H NMR referencing to coronene (0.25 mM) of *cis*- and *trans*-FAB (**a**) and naphthalene (**b**) in arm I and arm II solutions of System 2. Arm I was irradiated at 530 nm and 400 nm during the forward and reverse transport, respectively. Arm II was irradiated at 400 nm and 530 nm during the forward and reverse transport, respectively. The grey dashed line indicates the point which the LED light strips were swapped, switching from forward to reverse transport. Data are presented as mean values +/- measurement errors, derived from error propagation of the SD and the signal-to-noise ratio (n=22) of coronene (Supplementary Section 5).

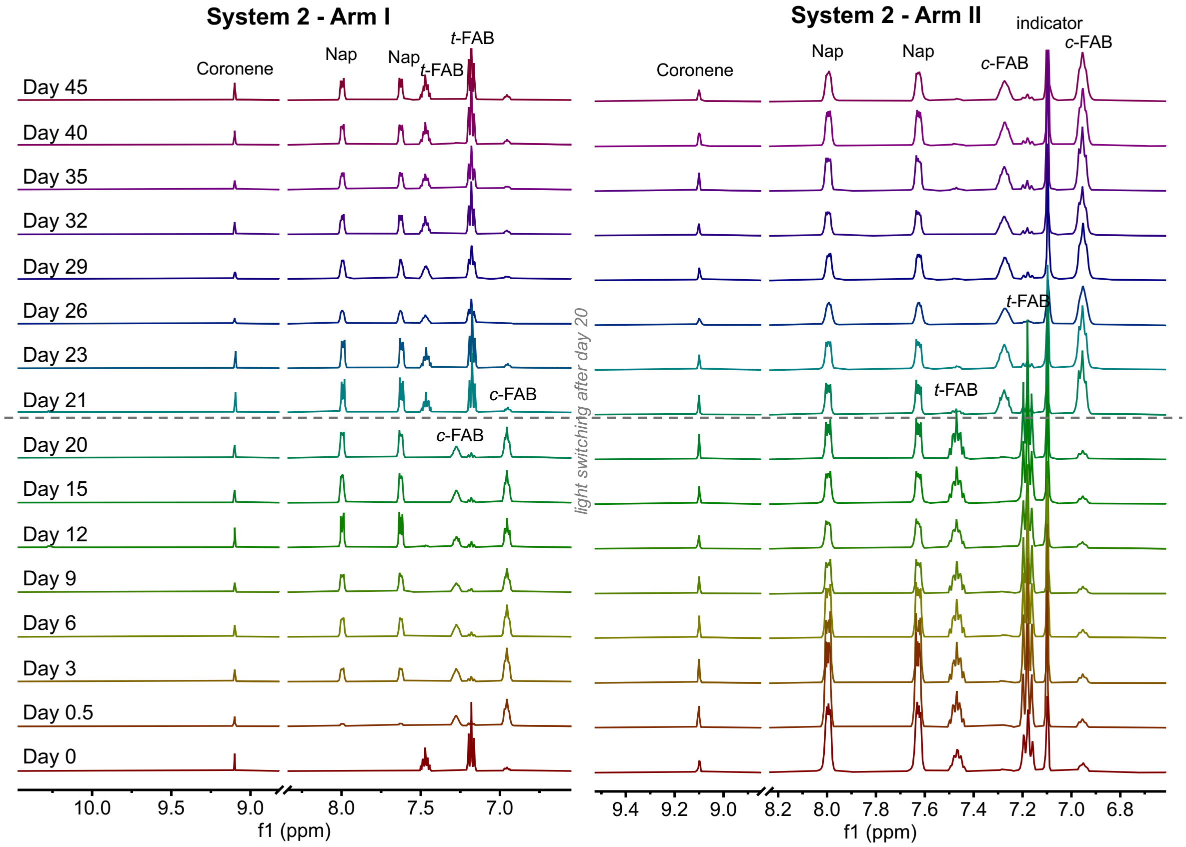

Supplementary Fig. 17. ^{1}H NMR spectra (500 MHz, D_2O, 298 K) of the measurements from System 2 at different times, showing the peaks for coronene, *trans*-FAB (*t*-FAB) and *cis*-FAB (*c*-FAB). The spectra on the left are for samples taken from arm I and the spectra on the right are for samples taken from arm II. The integrals of *trans*- and *cis*-FAB peak signals were referenced to coronene at 9.1 ppm, the integral of which was normalized to 100. Arm I was irradiated at 530 nm and 400 nm during forward and reverse transport, respectively. Arm II was irradiated at 400 nm and 530 nm during forward and reverse transport, respectively. The grey dashed line indicates the point which the LED light strips were swapped, separating forward and reverse transport..

Supplementary Table 3. Raw data from NMR integration, uncertainty and *trans*- and *cis*-FAB concentration values in arms I and II of System 2 measured by ^{1}H NMR referenced to coronene (0.25 mM). The green background color illustrates the period when the arms were irradiated at 530 nm, while the purple background color represents irradiation at 400 nm. The data are also included as a separate excel file as the Source Data for Supplementary Figures

System 3	Naphthalene Counterflow Augments Maxwell's Demon Experiment	
IS	0.25 mM coronene (normalized to 100)	
Error	Systematic	5%
	Noise level	2

System 2

Arm I	Arm I																					
	Time	cis-FAB									trans-FAB									FAB (total)		
	(day)	∫Hj-cis	∂Hj-cis	∫Hk-cis	∂Hk-cis	Fcis	∂Fcis	**[cis-FAB]**	∂[cis-FAB]	%∂[cis-FAB]	∫Hj-trans	∂Hj-trans	∫Hk-trans	∂Hk-trans	Ftrans	∂Ftrans	**[trans-FAB]**	∂[trans-FAB]	%∂[trans-FAB]	**[FAB] (I)**	∂[FAB] (I)	%∂[FAB] (I)
**Forward transport (530 nm)**	**0**	68.45	5.42	138.70	8.94	34.53	1.74	**1.04**	0.09	8.63	620.83	33.04	1238.14	63.91	309.83	11.99	**9.29**	0.74	8.00	**10.33**	0.75	7.25
	**0.5**	486.73	26.34	1014.63	52.73	250.23	9.82	**7.51**	0.60	8.03	57.67	4.88	137.77	8.89	32.57	1.69	**0.98**	0.09	8.71	**8.48**	0.61	7.17
	**3**	457.92	24.90	926.70	48.34	230.77	9.06	**6.92**	0.56	8.03	49.16	4.46	97.70	6.89	24.48	1.37	**0.73**	0.07	8.96	**7.66**	0.56	7.31
	**6**	452.81	24.64	924.39	48.22	229.53	9.03	**6.89**	0.55	8.03	51.74	4.59	100.31	7.02	25.34	1.40	**0.76**	0.07	8.91	**7.65**	0.56	7.28
	**9**	454.26	24.71	917.99	47.90	228.71	8.98	**6.86**	0.55	8.03	48.08	4.40	92.17	6.61	23.38	1.32	**0.70**	0.06	9.00	**7.56**	0.55	7.33
	**12**	444.00	24.20	902.37	47.12	224.40	8.83	**6.73**	0.54	8.03	38.78	3.94	92.56	6.63	21.89	1.29	**0.66**	0.06	9.14	**7.39**	0.54	7.36
	**15**	437.85	23.89	894.59	46.73	222.07	8.75	**6.66**	0.54	8.03	41.49	4.07	92.00	6.60	22.25	1.29	**0.67**	0.06	9.10	**7.33**	0.54	7.35
	**20**	427.63	23.38	894.49	46.72	220.35	8.71	**6.61**	0.53	8.04	41.32	4.07	93.89	6.69	22.54	1.31	**0.68**	0.06	9.09	**7.29**	0.53	7.34
**Reverse transport (400 nm)**	**21**	72.71	5.64	158.55	9.93	38.54	1.90	**1.16**	0.10	8.57	447.61	24.38	892.24	46.61	223.31	8.77	**6.70**	0.54	8.03	**7.86**	0.55	6.96
	**23**	52.27	4.61	121.59	8.08	28.98	1.55	**0.87**	0.08	8.81	503.07	27.15	1008.77	52.44	251.97	9.84	**7.56**	0.61	8.02	**8.43**	0.61	7.25
	**26**	48.74	4.44	134.38	8.72	30.52	1.63	**0.92**	0.08	8.81	543.10	29.16	1086.84	56.34	271.66	10.57	**8.15**	0.65	8.01	**9.07**	0.66	7.26
	**29**	56.05	4.80	133.09	8.65	31.52	1.65	**0.95**	0.08	8.74	576.27	30.81	1163.51	60.18	289.96	11.27	**8.70**	0.70	8.01	**9.64**	0.70	7.27
	**32**	62.58	5.13	139.41	8.97	33.67	1.72	**1.01**	0.09	8.67	613.58	32.68	1232.91	63.65	307.75	11.92	**9.23**	0.74	8.00	**10.24**	0.74	7.26
	**35**	52.48	4.62	153.65	9.68	34.36	1.79	**1.03**	0.09	8.72	621.67	33.08	1232.85	63.64	309.09	11.95	**9.27**	0.74	8.00	**10.30**	0.75	7.25
	**40**	50.67	4.53	147.48	9.37	33.03	1.74	**0.99**	0.09	8.75	623.39	33.17	1251.09	64.55	312.41	12.10	**9.37**	0.75	8.00	**10.36**	0.75	7.28
	**45**	57.36	4.87	149.85	9.49	34.54	1.78	**1.04**	0.09	8.69	626.02	33.30	1246.16	64.31	312.03	12.07	**9.36**	0.75	8.00	**10.40**	0.75	7.25

Arm II	Arm II																					
	Time	cis-FAB									trans-FAB									FAB (total)		
	(day)	∫Hj-cis	∂Hj-cis	∫Hk-cis	∂Hk-cis	Fcis	∂Fcis	**[cis-FAB]**	∂[cis-FAB]	%∂[cis-FAB]	∫Hj-trans	∂Hj-trans	∫Hk-trans	∂Hk-trans	Ftrans	∂Ftrans	**[trans-FAB]**	∂[trans-FAB]	%∂[trans-FAB]	**[FAB] (II)**	∂[FAB] (II)	%∂[FAB] (II)
**Forward transport (400 nm)**	**0**	72.97	5.65	237.54	13.88	51.75	2.50	**1.55**	0.13	8.50	628.98	33.45	1222.75	63.14	308.62	11.91	**9.26**	0.74	7.99	**10.81**	0.75	6.95
	**0.5**	65.09	5.25	162.50	10.13	37.93	1.90	**1.14**	0.10	8.61	669.33	35.47	1326.42	68.32	332.63	12.83	**9.98**	0.80	7.99	**11.12**	0.80	7.23
	**3**	55.63	4.78	161.37	10.07	36.17	1.86	**1.09**	0.09	8.68	683.33	36.17	1344.61	69.23	337.99	13.02	**10.14**	0.81	7.99	**11.22**	0.82	7.27
	**6**	66.42	5.32	160.98	10.05	37.90	1.90	**1.14**	0.10	8.60	697.92	36.90	1384.23	71.21	347.03	13.37	**10.41**	0.83	7.99	**11.55**	0.84	7.25
	**9**	69.83	5.49	174.80	10.74	40.77	2.01	**1.22**	0.10	8.56	747.11	39.36	1470.14	75.51	369.54	14.19	**11.09**	0.89	7.98	**12.31**	0.89	7.24
	**12**	61.68	5.08	166.26	10.31	37.99	1.92	**1.14**	0.10	8.63	757.75	39.89	1510.01	77.50	377.96	14.53	**11.34**	0.91	7.99	**12.48**	0.91	7.30
	**15**	51.10	4.56	175.39	10.77	37.75	1.95	**1.13**	0.10	8.70	780.14	41.01	1570.84	80.54	391.83	15.06	**11.75**	0.94	7.99	**12.89**	0.94	7.32
	**20**	72.62	5.63	164.92	10.25	39.59	1.95	**1.19**	0.10	8.56	791.86	41.59	1585.56	81.28	396.24	15.22	**11.89**	0.95	7.98	**13.07**	0.95	7.30
**Reverse transport (530 nm)**	**21**	692.17	36.61	1454.69	74.73	357.81	13.87	**10.73**	0.86	8.00	113.68	7.68	210.05	12.50	53.96	2.45	**1.62**	0.13	8.34	**12.35**	0.87	7.04
	**23**	671.92	35.60	1408.78	72.44	346.78	13.45	**10.40**	0.83	8.00	78.05	5.90	128.17	8.41	34.37	1.71	**1.03**	0.09	8.59	**11.43**	0.84	7.32
	**26**	625.69	33.28	1396.09	71.80	336.96	13.19	**10.11**	0.81	8.02	70.19	5.51	120.26	8.01	31.74	1.62	**0.95**	0.08	8.66	**11.06**	0.81	7.37
	**29**	611.54	32.58	1334.67	68.73	324.37	12.68	**9.73**	0.78	8.02	63.74	5.19	116.71	7.84	30.08	1.57	**0.90**	0.08	8.72	**10.63**	0.78	7.37
	**32**	603.11	32.16	1249.63	64.48	308.79	12.01	**9.26**	0.74	8.01	60.41	5.02	114.38	7.72	29.13	1.53	**0.87**	0.08	8.76	**10.14**	0.75	7.36
	**35**	564.28	30.21	1175.97	60.80	290.04	11.32	**8.70**	0.70	8.01	61.34	5.07	118.26	7.91	29.93	1.57	**0.90**	0.08	8.74	**9.60**	0.70	7.31
	**40**	556.07	29.80	1131.53	58.58	281.27	10.95	**8.44**	0.68	8.01	60.98	5.05	98.48	6.92	26.58	1.43	**0.80**	0.07	8.82	**9.24**	0.68	7.36
	**45**	543.80	29.19	1117.58	57.88	276.90	10.80	**8.31**	0.67	8.01	67.45	5.37	103.05	7.15	28.42	1.49	**0.85**	0.07	8.75	**9.16**	0.67	7.31

Supplementary Table 4. Raw data from NMR integration, uncertainty and naphthalene concentration values in arms I and II of System 2 measured by ^{1}H NMR referenced to coronene (0.25 mM). The green background color illustrates the period when the arms were irradiated at 530 nm, while the purple background color represents irradiation at 400 nm. The data are also included as a separate excel file as the Source Data for Supplementary Figures

System 2 - Naphthalene

Arm I	Arm I									
	Time	Naphthalene (Arm I)								
	(day)	∫Ha	∂Ha	∫Hb	∂Hb	N	∂N	[Naphthalene] (I)	∂[Naphthalene] (I)	%∂[Naphthalene] (I)
Forward transport (530 nm)	**0**	0.00	2.00	0.00	2.00	0.00	1.41	**0.00**	0.00	0.00
	**0.5**	84.25	6.21	92.21	6.61	88.23	4.54	**0.66**	0.06	8.68
	**3**	345.80	19.29	342.53	19.13	344.17	13.58	**2.58**	0.21	8.04
	**6**	505.12	27.26	501.95	27.10	503.54	19.22	**3.78**	0.30	7.97
	**9**	666.93	35.35	662.96	35.15	664.95	24.92	**4.99**	0.40	7.94
	**12**	742.06	39.10	725.39	38.27	733.73	27.36	**5.50**	0.44	7.93
	**15**	729.49	38.47	728.82	38.44	729.16	27.19	**5.47**	0.43	7.93
	**20**	727.20	38.36	720.91	38.05	724.06	27.01	**5.43**	0.43	7.93
Reverse transport (400 nm)	**21**	677.01	35.85	683.30	36.17	680.16	25.46	**5.10**	0.40	7.94
	**23**	662.96	35.15	665.07	35.25	664.02	24.89	**4.98**	0.40	7.94
	**26**	640.85	34.04	640.57	34.03	640.71	24.07	**4.81**	0.38	7.94
	**29**	621.19	33.06	638.76	33.94	629.98	23.69	**4.72**	0.38	7.95
	**32**	593.40	31.67	614.51	32.73	603.96	22.77	**4.53**	0.36	7.95
	**35**	555.37	29.77	550.41	29.52	552.89	20.96	**4.15**	0.33	7.96
	**40**	509.79	27.49	512.62	27.63	511.21	19.49	**3.83**	0.31	7.97
	**45**	470.96	25.55	485.52	26.28	478.24	18.32	**3.59**	0.29	7.98

Arm II	Arm II									
	Time	Naphthalene (Arm II)								
	(day)	∫Ha	∂Ha	∫Hb	∂Hb	N	∂N	[Naphthalene] (I)	∂[Naphthalene] (I)	%∂[Naphthalene] (I)
Forward transport (400 nm)	**0**	1515.94	77.80	1507.80	77.39	1511.87	54.87	**11.34**	0.89	7.88
	**0.5**	1393.57	71.68	1378.82	70.94	1386.20	50.42	**10.40**	0.82	7.89
	**3**	1056.76	54.84	1048.08	54.40	1052.42	38.62	**7.89**	0.62	7.90
	**6**	889.89	46.49	881.49	46.07	885.69	32.73	**6.64**	0.53	7.92
	**9**	707.51	37.38	711.36	37.57	709.44	26.50	**5.32**	0.42	7.93
	**12**	655.05	34.75	659.57	34.98	657.31	24.65	**4.93**	0.39	7.94
	**15**	645.77	34.29	629.39	33.47	637.58	23.96	**4.78**	0.38	7.94
	**20**	636.35	33.82	622.49	33.12	629.42	23.67	**4.72**	0.38	7.95
Reverse transport (530 nm)	**21**	611.62	32.58	607.01	32.35	609.32	22.96	**4.57**	0.36	7.95
	**23**	597.34	31.87	584.72	31.24	591.03	22.31	**4.43**	0.35	7.95
	**26**	633.45	33.67	630.23	33.51	631.84	23.75	**4.74**	0.38	7.95
	**29**	571.52	30.58	582.27	31.11	576.90	21.81	**4.33**	0.34	7.96
	**32**	578.72	30.94	567.71	30.39	573.22	21.68	**4.30**	0.34	7.96
	**35**	575.78	30.79	577.90	30.90	576.84	21.81	**4.33**	0.34	7.96
	**40**	594.99	31.75	585.68	31.28	590.34	22.29	**4.43**	0.35	7.95
	**45**	602.69	32.13	591.87	31.59	597.28	22.53	**4.48**	0.36	7.95

The UV-Vis absorption of *cis*-FAB and *trans*-FAB in arms I and II were also measured, respectively, The isomer concentrations were calculated based on the maximum absorption of *trans*-FAB (at 465 nm) and *cis*-FAB (at 415 nm) using a linear fit in Supplementary Fig. 11. *Trans*-FAB was observed to accumulate in arm II, while *cis*-FAB was depleted in arm I. Compared to the transport of *cis*-FAB in the absence of naphthalene described in Supplementary Section 7, the shift in the FAB concentration in arm I and arm II is more significant with naphthalene in arm II at a steady state. The UV-Vis results aligned with the NMR results.

After 20 days, the light irradiation was switched between the two arms and the flow of FAB was reversed (arm II to arm I).

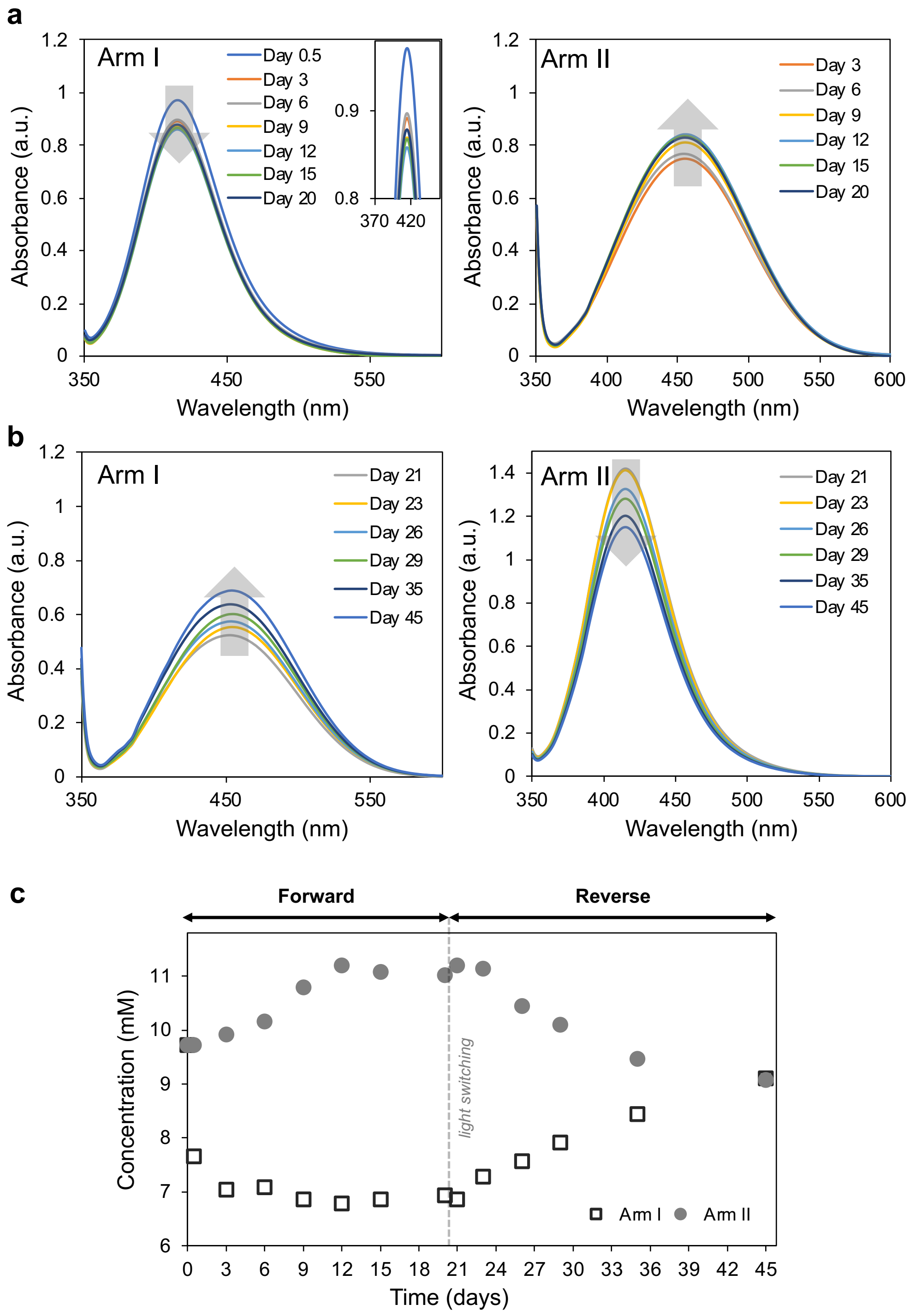

Supplementary Fig. 18. UV-Vis measurements for System 2. **a**, UV-Vis absorption of FAB solution in arm I and arm II during the forward transport process. *Trans*- and *cis*-FAB concentrations in arm I and arm II were calculated and plotted as function of time. **b**, UV-Vis absorption of FAB solution in arm I and II in the reversed transport process. While *trans*-FAB

concentration was observed to accumulated in arm I, *cis*-FAB was depleted in arm II. **c**, Concentration of *trans*-FAB and *cis*-FAB in arm I and arm II were calculated using linear fitting equations for both forward and reversed processes. Arm I was irradiated at 530 nm and 400 nm during the forward and reverse transport, respectively. Arm II was irradiated at 400 nm and 530 nm during the forward and reverse transport, respectively. The grey dashed line indicates the point which the LED light strips were swapped, separating forward and reverse transport. Unlike NMR, the UV-Vis measurement does not provide unique signals for each FAB isomer without data processing by deconvolution. Thus, the quantification of our experimental results was performed using the NMR experimental results. UV-Vis experiments were performed as a qualitative check on each experiment.

Supplementary Table 5. Maximum absorbance values from UV-Vis measurements and FAB concentration values in arms I and II in System 2. Concentration of FAB in arm I and arm II were calculated using linear fitting on the maximum absorbance for both forward and reverse processes according to Supplementary Fig. 11. The green background illustrates the period when the arms were irradiated at 530 nm, while the purple background represents light irradiation at 400 nm. The data are also included as a separate excel file as the Source Data for Supplementary Figures

**System 2 - UV**									
**Arm I**					**Arm II**				
**Arm I**	**Time (days)**	**Max Abs (a.u.)**	**Concentration (mM)**		**Arm II**	**Time (days)**	**Max Abs (a.u.)**	**Concentration (mM)**
**Forward transport (530 nm)**	**0**	0.73	**9.72**	*light switching on day 20*	**Forward transport (400 nm)**	**0**	0.73	**9.72**
	**0.5**	0.97	**7.66**			**0.5**	0.73	**9.72**
	**3**	0.89	**7.04**			**3**	0.75	**9.92**
	**6**	0.90	**7.09**			**6**	0.77	**10.16**
	**9**	0.87	**6.87**			**9**	0.81	**10.79**
	**12**	0.86	**6.78**			**12**	0.84	**11.19**
	**15**	0.87	**6.86**			**15**	0.83	**11.08**
	**20**	0.88	**6.94**			**20**	0.83	**11.02**
**Reverse transport (400 nm)**	**21**	0.52	**6.87**		**Reverse transport (530 nm)**	**21**	1.42	**11.19**
	**23**	0.55	**7.28**			**23**	1.41	**11.14**
	**26**	0.58	**7.57**			**26**	1.32	**10.44**
	**29**	0.60	**7.92**			**29**	1.28	**10.09**
	**35**	0.64	**8.44**			**35**	1.20	**9.47**
	**45**	0.69	**9.10**			**45**	1.15	**9.07**

9. Maxwell's Demon Drives Naphthalene Counterflow experiment (System 3): experimental results

In System 3, the U-tube was prepared by adding an aqueous solution of cage **1** (4 mM, 2.5 mL, 25 mol% relative to the total FAB in both arms) into the bottom of the U-tube (internal diameter 1.2 cm), and aliquots of a dodecane solution containing FAB (10 mM, 2 mL, 90% *trans*) and naphthalene (6 mM) into each arm. The solutions contained coronene (0.25 mM) as an internal standard. In addition, arm II contained triisopropylbenzene (10 mM) as an indicator. During the experiment, the cage layer was stirred at 250 rpm at room temperature with a cylindrical magnetic stir bar (3 x 6 mm). During the first 20 days, arm I was irradiated at 530 nm with a LED light strip, and arm II at 400 nm. After 20 days, the LED light strips were exchanged, where arm I was irradiated at 400 nm, and arm II at 530 nm.

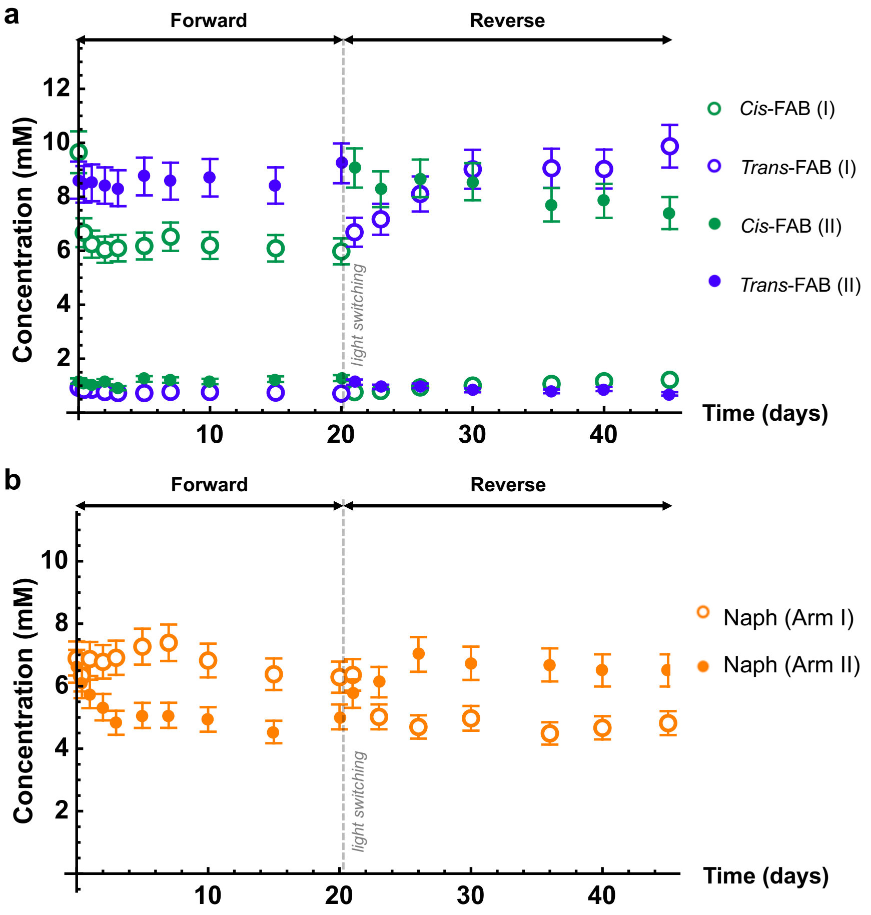

Supplementary Fig. 19. Graph showing experimental results measured by ^{1}H NMR referencing to coronene (0.25 mM) of *cis*- and *trans*-FAB (**a**) and naphthalene (**b**) in arm I and arm II solutions of System 3. Arm I was irradiated at 530 nm and 400 nm during the forward and reverse transport, respectively. Arm II was irradiated at 400 nm and 530 nm during the forward and reverse transport, respectively. The grey dashed line indicates the point which the LED light strips were swapped, switching from forward to reverse transport. Data are presented as mean values +/- measurement errors, derived from error propagation of the SD and the signal-to-noise ratio (n=22) of coronene (Supplementary Section 5).

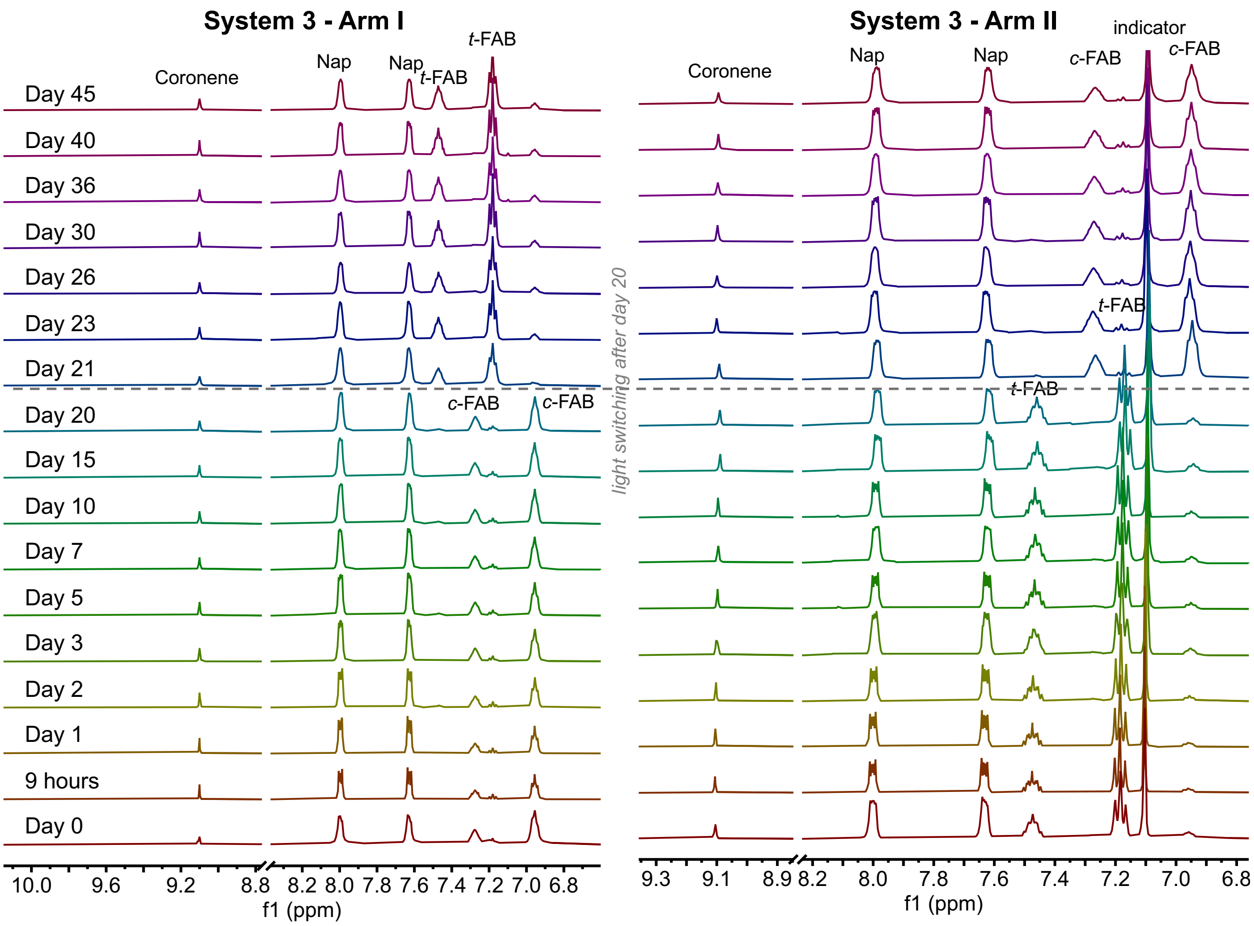

Supplementary Fig. 20. ^{1}H NMR spectra (500 MHz, D_2O, 298 K) of the measurements from System 3 at different times, showing the peaks for coronene, *trans*-FAB (*t*-FAB) and *cis*-FAB (*c*-FAB). The spectra on the left are for the samples taken from arm I and the spectra on the right are for the samples taken from arm II. The integrals of the *trans*- and *cis*-FAB peak signals were referenced to coronene at 9.1 ppm, the integral of which was normalized to 100. Arm I was irradiated at 530 nm and 400 nm during forward and reverse transport, respectively. Arm II was irradiated at 400 nm and 530 nm during forward and reverse transport, respectively. The grey dashed line indicates the point which the LED light strips were swapped, separating forward and the reverse transport

Supplementary Table 6. Raw data from NMR integration, uncertainty and *trans*- and *cis*-FAB concentration values in arms I and II of System 3 measured by ^{1}H NMR referenced to coronene (0.25 mM). The green background color illustrates the period when the arms were irradiated at 530 nm, while the purple background color represents irradiation at 400 nm. The data are also included as a separate excel file as the Source Data for Supplementary Figures

System 3	Maxwell's Demon Drives Naphthalene Counterflow Experiment	
IS	0.25 mM coronene (normalized to 100)	
Error	Systematic	5%
	Noise level	2

System 3

Arm I

Arm I	Time (day)	cis-FAB ∫Hj-cis	∂Hj-cis	∫Hk-cis	∂Hk-cis	Fcis	∂Fcis	[cis-FAB]	∂[cis-FAB]	%∂[cis-FAB]	trans-FAB ∫Hj-trans	∂Hj-trans	∫Hk-trans	∂Hk-trans	Ftrans	∂Ftrans	[trans-FAB]	∂[trans-FAB]	%∂[trans-FAB]	FAB (total) [FAB] (I)	∂[FAB] (I)	%∂[FAB] (I)
Forward transport (530 nm)	**0**	600.93	32.05	1329.20	68.46	321.69	12.60	**9.65**	0.77	8.02	74.55	5.73	110.44	7.52	30.83	1.58	**0.92**	0.08	8.67	**10.58**	0.78	7.36
	**0.38**	444.90	24.25	889.18	46.46	222.35	8.73	**6.67**	0.54	8.03	66.37	5.32	103.34	7.17	28.29	1.49	**0.85**	0.07	8.76	**7.52**	0.54	7.19
	**0.92**	406.62	22.33	842.46	44.12	208.18	8.24	**6.25**	0.50	8.04	63.15	5.16	107.55	7.38	28.45	1.50	**0.85**	0.07	8.76	**7.10**	0.51	7.15
	**2**	394.14	21.71	814.10	42.71	201.37	7.98	**6.04**	0.49	8.04	58.89	4.94	94.31	6.72	25.53	1.39	**0.77**	0.07	8.87	**6.81**	0.49	7.21
	**3**	399.32	21.97	820.08	43.00	203.23	8.05	**6.10**	0.49	8.04	56.88	4.84	89.10	6.46	24.33	1.35	**0.73**	0.07	8.92	**6.83**	0.49	7.25
	**5**	400.60	22.03	834.56	43.73	205.86	8.16	**6.18**	0.50	8.04	55.02	4.75	90.68	6.53	24.28	1.35	**0.73**	0.07	8.93	**6.90**	0.50	7.26
	**7**	427.27	23.36	877.13	45.86	217.40	8.58	**6.52**	0.52	8.04	58.67	4.93	96.06	6.80	25.79	1.40	**0.77**	0.07	8.86	**7.30**	0.53	7.24
	**10**	402.89	22.14	836.20	43.81	206.52	8.18	**6.20**	0.50	8.04	62.12	5.11	91.23	6.56	25.56	1.39	**0.77**	0.07	8.85	**6.96**	0.50	7.22
	**15**	387.87	21.39	830.58	43.53	203.08	8.08	**6.09**	0.49	8.05	57.89	4.89	91.46	6.57	24.89	1.37	**0.75**	0.07	8.89	**6.84**	0.50	7.24
	**20**	382.83	21.14	812.91	42.65	199.29	7.93	**5.98**	0.48	8.05	53.97	4.70	88.21	6.41	23.70	1.32	**0.71**	0.06	8.96	**6.69**	0.49	7.26
Reverse transport (400 nm)	**21**	54.18	4.71	98.44	6.92	25.44	1.40	**0.76**	0.07	8.89	465.27	25.26	872.09	45.60	222.89	8.69	**6.69**	0.54	8.01	**7.45**	0.54	7.25
	**23**	48.63	4.43	112.31	7.62	26.82	1.47	**0.80**	0.07	8.89	494.90	26.75	938.50	48.93	238.90	9.29	**7.17**	0.57	8.01	**7.97**	0.58	7.26
	**26**	51.20	4.56	136.35	8.82	31.26	1.65	**0.94**	0.08	8.78	535.03	28.75	1085.83	56.29	270.14	10.53	**8.10**	0.65	8.01	**9.04**	0.65	7.24
	**30**	47.44	4.37	153.54	9.68	33.50	1.77	**1.00**	0.09	8.77	603.83	32.19	1200.09	62.00	300.65	11.64	**9.02**	0.72	8.00	**10.02**	0.73	7.25
	**36**	50.33	4.52	162.91	10.15	35.54	1.85	**1.07**	0.09	8.72	615.61	32.78	1196.01	61.80	301.94	11.66	**9.06**	0.72	7.99	**10.12**	0.73	7.21
	**40**	74.67	5.73	158.33	9.92	38.83	1.91	**1.17**	0.10	8.55	615.66	32.78	1189.96	61.50	300.94	11.62	**9.03**	0.72	7.99	**10.19**	0.73	7.15
	**45**	70.98	5.55	172.79	10.64	40.63	2.00	**1.22**	0.10	8.56	647.00	34.35	1327.58	68.38	329.10	12.75	**9.87**	0.79	8.00	**11.09**	0.80	7.18

Arm II

Arm II	Time (day)	cis-FAB ∫Hj-cis	∂Hj-cis	∫Hk-cis	∂Hk-cis	Fcis	∂Fcis	[cis-FAB]	∂[cis-FAB]	%∂[cis-FAB]	trans-FAB ∫Hj-trans	∂Hj-trans	∫Hk-trans	∂Hk-trans	Ftrans	∂Ftrans	[trans-FAB]	∂[trans-FAB]	%∂[trans-FAB]	FAB (total) [FAB] (II)	∂[FAB] (II)	%∂[FAB] (II)
Forward transport (400 nm)	**0**	76.00	5.80	158.57	9.93	39.10	1.92	**1.17**	0.10	8.55	602.70	32.14	1120.60	58.03	287.22	11.06	**8.62**	0.69	7.99	**9.79**	0.70	7.11
	**0.38**	82.30	6.12	137.04	8.85	36.56	1.79	**1.10**	0.09	8.55	579.33	30.97	1112.71	57.64	282.01	10.90	**8.46**	0.68	8.00	**9.56**	0.68	7.15
	**0.92**	72.65	5.63	137.46	8.87	35.02	1.75	**1.05**	0.09	8.60	584.14	31.21	1119.30	57.97	283.91	10.97	**8.52**	0.68	8.00	**9.57**	0.69	7.18
	**2**	80.38	6.02	149.96	9.50	38.39	1.87	**1.15**	0.10	8.53	577.10	30.86	1106.09	57.30	280.53	10.85	**8.42**	0.67	8.00	**9.57**	0.68	7.11
	**3**	69.17	5.46	113.10	7.66	30.38	1.57	**0.91**	0.08	8.70	559.03	29.95	1104.55	57.23	277.26	10.77	**8.32**	0.67	8.00	**9.23**	0.67	7.27
	**5**	83.46	6.17	166.61	10.33	41.68	2.01	**1.25**	0.11	8.49	619.95	33.00	1130.41	58.52	291.73	11.20	**8.75**	0.70	7.98	**10.00**	0.71	7.07
	**7**	75.71	5.79	166.29	10.31	40.33	1.97	**1.21**	0.10	8.54	610.16	32.51	1105.51	57.28	285.95	10.98	**8.58**	0.68	7.98	**9.79**	0.69	7.08
	**10**	79.06	5.95	152.54	9.63	38.60	1.89	**1.16**	0.10	8.54	607.52	32.38	1133.55	58.68	290.18	11.17	**8.71**	0.70	7.99	**9.86**	0.70	7.12
	**15**	74.75	5.74	173.90	10.70	41.44	2.02	**1.24**	0.11	8.53	608.75	32.44	1074.84	55.74	280.60	10.75	**8.42**	0.67	7.98	**9.66**	0.68	7.04
	**20**	67.81	5.39	188.73	11.44	42.76	2.11	**1.28**	0.11	8.56	652.66	34.63	1195.26	61.76	307.99	11.80	**9.24**	0.74	7.98	**10.52**	0.75	7.08
Reverse transport (530 nm)	**21**	601.10	32.06	1212.28	62.61	302.23	11.72	**9.07**	0.73	8.00	77.92	5.90	148.71	9.44	37.77	1.85	**1.13**	0.10	8.55	**10.20**	0.73	7.18
	**23**	549.85	29.49	1106.18	57.31	276.01	10.74	**8.28**	0.66	8.01	65.82	5.29	125.25	8.26	31.85	1.64	**0.96**	0.08	8.68	**9.24**	0.67	7.24
	**26**	564.29	30.21	1172.37	60.62	289.44	11.29	**8.68**	0.70	8.01	70.57	5.53	128.48	8.42	33.18	1.68	**1.00**	0.09	8.64	**9.68**	0.70	7.24
	**30**	537.28	28.86	1174.47	60.72	285.29	11.21	**8.56**	0.69	8.03	62.13	5.11	104.09	7.20	27.70	1.47	**0.83**	0.07	8.79	**9.39**	0.69	7.36
	**36**	469.66	25.48	1071.35	55.57	256.84	10.19	**7.71**	0.62	8.05	67.05	5.35	91.19	6.56	26.37	1.41	**0.79**	0.07	8.81	**8.50**	0.62	7.34
	**40**	501.98	27.10	1068.16	55.41	261.69	10.28	**7.85**	0.63	8.03	60.48	5.02	115.22	7.76	29.28	1.54	**0.88**	0.08	8.76	**8.73**	0.63	7.27
	**45**	470.82	25.54	1008.54	52.43	246.56	9.72	**7.40**	0.59	8.03	57.73	4.89	81.95	6.10	23.28	1.30	**0.70**	0.06	8.96	**8.10**	0.60	7.38

Supplementary Table 7. Raw data from NMR integration, uncertainty and naphthalene concentration values in arms I and II in System 3 measured by ^{1}H NMR, referenced to coronene (0.25 mM). The green background illustrates the period when the arms were irradiated at 530 nm, while the purple background represents irradiation at 400 nm. The data are also included as a separate excel file as the Source Data for Supplementary Figures

System 3 - Naphthalene

Arm I	Arm I									
	Time (day)	Naphthalene (Arm I)								
		∫Ha	∂Ha	∫Hb	∂Hb	N	∂N	**[Naphthalene] (I)**	∂[Naphthalene] (I)	%∂[Naphthalene] (I)
Forward transport (530 nm)	**0**	916.39	47.82	920.16	48.01	918.28	33.88	**6.89**	0.54	7.91
	**0.38**	844.28	44.21	844.78	44.24	844.53	31.27	**6.33**	0.50	7.92
	**0.92**	915.94	47.80	916.19	47.81	916.07	33.80	**6.87**	0.54	7.91
	**2**	901.37	47.07	906.60	47.33	903.99	33.38	**6.78**	0.54	7.91
	**3**	922.07	48.10	920.61	48.03	921.34	33.99	**6.91**	0.55	7.91
	**5**	967.53	50.38	969.92	50.50	968.73	35.66	**7.27**	0.57	7.91
	**7**	978.75	50.94	991.84	51.59	985.30	36.25	**7.39**	0.58	7.91
	**10**	914.50	47.73	904.63	47.23	909.57	33.57	**6.82**	0.54	7.91
	**15**	854.34	44.72	847.87	44.39	851.11	31.51	**6.38**	0.51	7.92
	**20**	844.63	44.23	832.44	43.62	838.54	31.06	**6.29**	0.50	7.92
Reverse transport (400 nm)	**21**	903.16	47.16	793.23	41.66	848.20	31.46	**6.36**	0.50	7.92
	**23**	675.93	35.80	662.41	35.12	669.17	25.07	**5.02**	0.40	7.94
	**26**	645.79	34.29	606.29	32.31	626.04	23.56	**4.70**	0.37	7.95
	**30**	682.00	36.10	643.77	34.19	662.89	24.86	**4.97**	0.39	7.94
	**36**	596.78	31.84	600.10	32.01	598.44	22.57	**4.49**	0.36	7.95
	**40**	644.51	34.23	600.12	32.01	622.32	23.43	**4.67**	0.37	7.95
	**45**	667.34	35.37	617.09	32.85	642.22	24.14	**4.82**	0.38	7.95

Arm II	Arm II									
	Time (day)	Naphthalene (Arm II)								
		∫Ha	∂Ha	∫Hb	∂Hb	N	∂N	**[Naphthalene] (I)**	∂[Naphthalene] (I)	%∂[Naphthalene] (I)
Forward transport (400 nm)	**0**	888.77	46.44	881.09	46.05	884.93	32.70	**6.64**	0.53	7.92
	**0.38**	815.38	42.77	811.18	42.56	813.28	30.17	**6.10**	0.48	7.92
	**0.92**	764.64	40.23	769.28	40.46	766.96	28.53	**5.75**	0.46	7.93
	**2**	707.02	37.35	713.92	37.70	710.47	26.53	**5.33**	0.42	7.93
	**3**	651.57	34.58	636.18	33.81	643.88	24.18	**4.83**	0.38	7.94
	**5**	681.00	36.05	669.68	35.48	675.34	25.29	**5.07**	0.40	7.94
	**7**	680.34	36.02	671.22	35.56	675.78	25.31	**5.07**	0.40	7.94
	**10**	661.90	35.10	654.11	34.71	658.01	24.68	**4.94**	0.39	7.94
	**15**	608.24	32.41	600.71	32.04	604.48	22.79	**4.53**	0.36	7.95
	**20**	671.79	35.59	666.20	35.31	669.00	25.07	**5.02**	0.40	7.94
Reverse transport (530 nm)	**21**	762.18	40.11	774.84	40.74	768.51	28.59	**5.76**	0.46	7.93
	**23**	811.03	42.55	823.47	43.17	817.25	30.31	**6.13**	0.49	7.92
	**26**	928.50	48.43	942.67	49.13	935.59	34.49	**7.02**	0.56	7.91
	**30**	892.46	46.62	903.16	47.16	897.81	33.16	**6.73**	0.53	7.91
	**36**	901.14	47.06	880.74	46.04	890.94	32.92	**6.68**	0.53	7.92
	**40**	857.36	44.87	877.33	45.87	867.35	32.08	**6.51**	0.52	7.92
	**45**	852.27	44.61	882.33	46.12	867.30	32.08	**6.50**	0.51	7.92

The UV-Vis absorption of *cis*-FAB and *trans*-FAB in arms I and II were also measured. The isomer concentrations were calculated based on the maximum absorption of *trans*-FAB (at 465 nm) and *cis*-FAB (at 415 nm) using linear fitting equations in Supplementary Fig. 11.

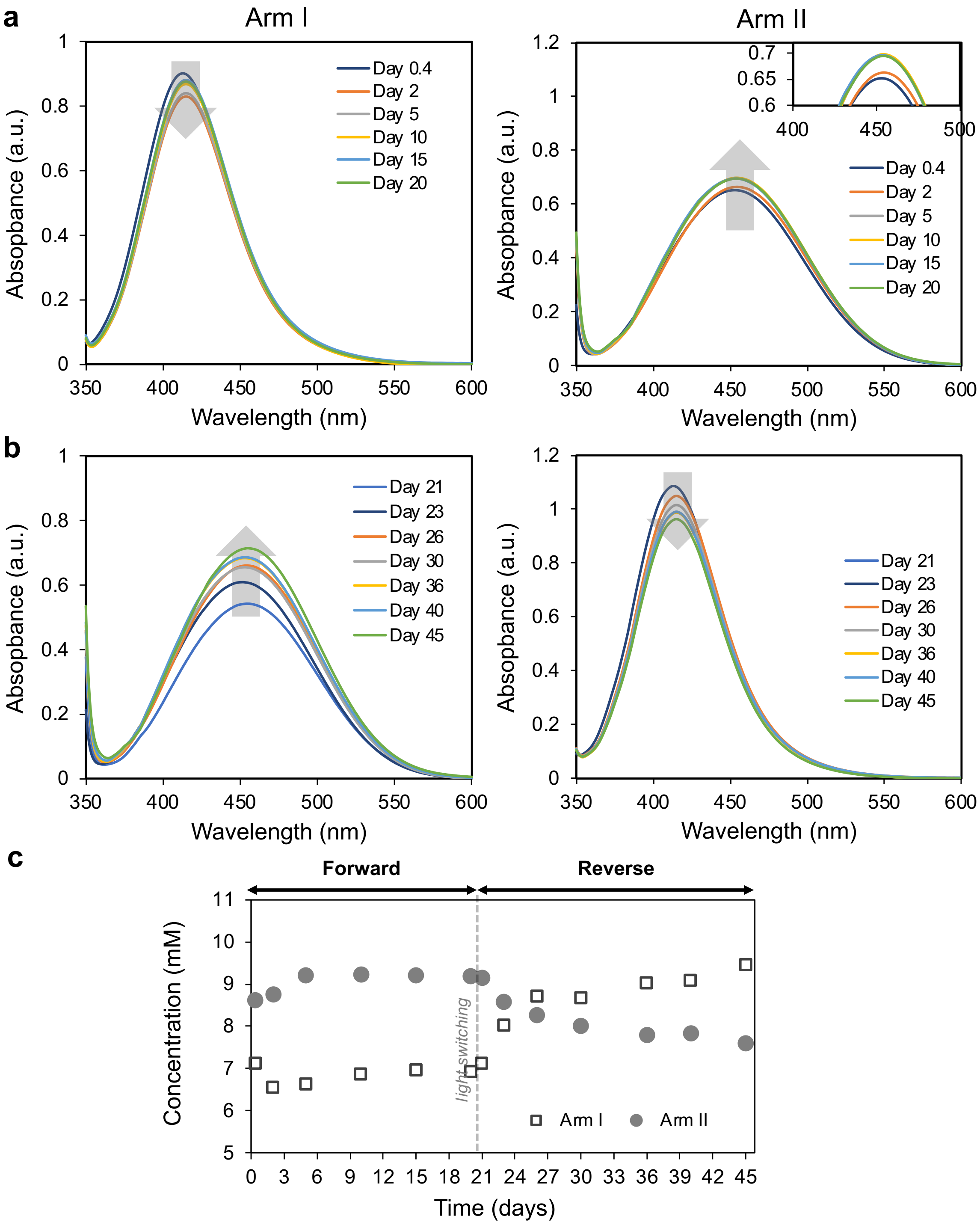

Supplementary Fig. 21. UV-Vis measurements for System 3. UV-Vis absorption spectra of FAB in arm I and arm II during the forward transport during (**a**) forward and (**b**) reverse process. **c**, Concentration of *trans*-FAB and *cis*-FAB in arm I and arm II were calculated using linear fitting equations for both forward and reverse processes. Arm I was irradiated at 530 nm and 400 nm during the forward and reverse transport, respectively. Arm II was irradiated at 400 nm and 530 nm during the forward and reverse transport, respectively. The grey dashed line indicates the point

which the LED light strips were swapped, switching from forward to reverse transport. Unlike NMR, the UV-Vis measurement does not provide unique signals for each FAB isomer without data processing by deconvolution. Thus, the quantification of our experimental results was performed using the NMR experimental results. UV-Vis experiments were performed as a qualitative check on each experiment.

Supplementary Table 8. Maximum absorbance from UV measurements and FAB concentration values in arm I and arm II solutions of System 3. Concentrations of FAB in arm I and arm II were calculated using linear fitting equations to the maximum absorbance for both forward and reversed processes as shown in Supplementary Fig. 11. The green background illustrates the period when the arms were irradiated at 530 nm, while the purple background represents irradiation at 400 nm. The data are also included as a separate excel file as the Source Data for Supplementary Figures

System 3 - UV								
Arm I					Arm II			
Arm I	Time (days)	Max Abs (a.u.)	Concentration (mM)		Arm II	Time (days)	Max Abs (a.u.)	Concentration (mM)
Forward transport (530 nm)	0.38	0.9	**7.12**	*light switching on day 20*	Forward transport (400 nm)	0.38	0.9	**7.12**
	2	0.83	**6.55**			2	0.83	**6.55**
	5	0.84	**6.63**			5	0.84	**6.63**
	10	0.87	**6.87**			10	0.87	**6.87**
	15	0.88	**6.96**			15	0.88	**6.96**
	20	0.88	**6.92**			20	0.88	**6.92**
Reverse transport (400 nm)	21	0.54	**7.11**		Reverse transport (530 nm)	21	0.54	**7.11**
	23	0.61	**8.02**			23	0.61	**8.02**
	26	0.66	**8.71**			26	0.66	**8.71**
	30	0.66	**8.67**			30	0.66	**8.67**
	36	0.68	**9.02**			36	0.68	**9.02**
	40	0.69	**9.08**			40	0.69	**9.08**
	45	0.71	**9.46**			45	0.71	**9.46**

10. Model prediction for Maxwell's Demon experiment (System 1)

Differential rate equations describing the proposed kinetic model (Supplementary Fig. 22) were written defining the change in concentrations of each species in the system. The predictions of the concentrations of each FAB isomer in each arm were calculated using Mathematica (Supplementary Fig. 23) by solving a set of differential rate equations and mass balance equations listed below (Eq. 9-26).

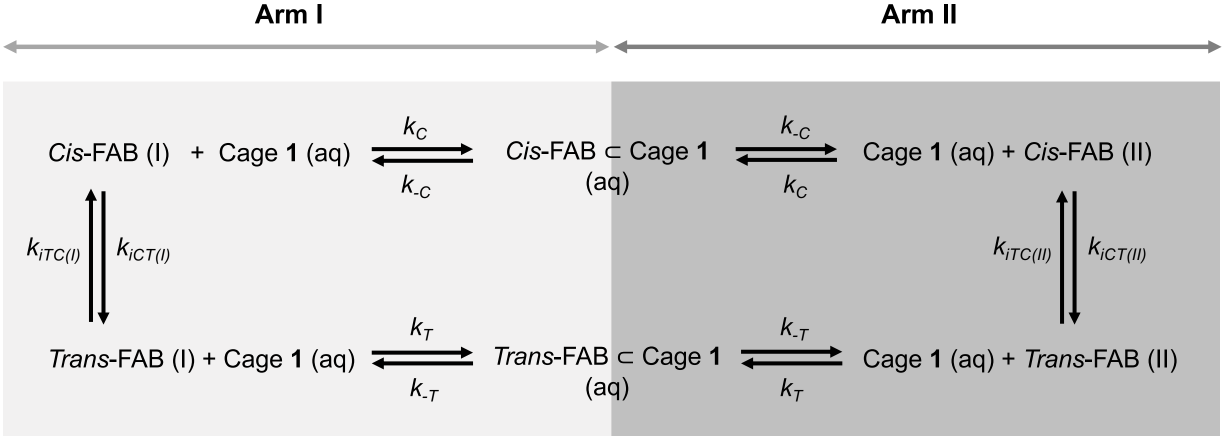

Supplementary Fig. 22. Kinetic model used to describe System 1.

For the forward transport:

Eq. 9: $\frac{d[\textit{Cis}\text{-FAB(I)}]}{dt} = -k_{\mathrm{C}}[\textit{Cis}\text{-FAB(I)}]\left(\frac{[Cage\ \mathbf{1}(aq)]}{V_{ratio}}\right) + k_{-\mathrm{C}}\frac{[\textit{Cis}\text{-FAB} \subset \text{Cage } \mathbf{1}\text{(aq)}]}{V_{ratio}} + k_{iTC,530nm}[\textit{Trans}\text{-FAB(I)}] - k_{iCT,530nm}[\textit{Cis}\text{-FAB(I)}]$

Eq. 10: $\frac{d[\textit{Trans}\text{-FAB(I)}]}{dt} = -k_{\mathrm{T}}[\textit{Trans}\text{-FAB(I)}]\left(\frac{[Cage\ \mathbf{1}(aq)]}{V_{ratio}}\right) + k_{-\mathrm{T}}\frac{[\textit{Trans}\text{-FAB} \subset \text{Cage } \mathbf{1}\text{(aq)}]}{V_{ratio}} - k_{iTC,530nm}[\textit{Trans}\text{-FAB(I)}] + k_{iCT,530nm}[\textit{Cis}\text{-FAB(I)}]$

Eq. 11: $\frac{d[\textit{Cis}\text{-FAB(II)}]}{dt} = -k_{\mathrm{C}}[\textit{Cis}\text{-FAB(II)}]\left(\frac{[Cage\ \mathbf{1}(aq)]}{V_{ratio}}\right) + k_{-\mathrm{C}}\frac{[\textit{Cis}\text{-FAB} \subset \text{Cage } \mathbf{1}\text{(aq)}]}{V_{ratio}} + k_{iTC,400nm}[\textit{Trans}\text{-FAB(II)}] - k_{iCT,400nm}[\textit{Cis}\text{-FAB(II)}]$

Eq. 12: $\frac{d[\textit{Trans}\text{-FAB(II)}]}{dt} = -k_{\mathrm{T}}[\textit{Trans}\text{-FAB(II)}]\left(\frac{[Cage\ \mathbf{1}(aq)]}{V_{ratio}}\right) + k_{-\mathrm{T}}\frac{[\textit{Trans}\text{-FAB} \subset \text{Cage } \mathbf{1}\text{(aq)}]}{V_{ratio}} - k_{iTC,400nm}[\textit{Trans}\text{-FAB(II)}] + k_{iCT,400nm}[\textit{Cis}\text{-FAB(II)}]$

Eq. 13: $\frac{d[\textit{Cis}\text{-FAB} \subset \text{Cage } \mathbf{1}\text{(aq)}]}{dt} = k_{\mathrm{C}}[\textit{Cis}\text{-FAB(I)}]\left(\frac{[Cage\ \mathbf{1}(aq)]}{V_{ratio}}\right) + k_{\mathrm{C}}[\textit{Cis}\text{-FAB(II)}]\left(\frac{[Cage\ \mathbf{1}(aq)]}{V_{ratio}}\right) - 2k_{-\mathrm{C}}\frac{[\textit{Cis}\text{-FAB} \subset \text{Cage } \mathbf{1}\text{(aq)}]}{V_{ratio}}$

Eq. 14: $\frac{d[\textit{Trans}\text{-FAB} \subset \text{Cage } \mathbf{1}\text{(aq)}]}{dt} = k_{\mathrm{T}}[\textit{Trans}\text{-FAB(I)}]\left(\frac{[Cage\ \mathbf{1}(aq)]}{V_{ratio}}\right) + k_{\mathrm{T}}[\textit{Trans}\text{-FAB(II)}]\left(\frac{[Cage\ \mathbf{1}(aq)]}{V_{ratio}}\right) - 2k_{-\mathrm{T}}\frac{[\textit{Trans}\text{-FAB} \subset \text{Cage } \mathbf{1}\text{(aq)}]}{V_{ratio}}$

Eq. 15: $\frac{d[\text{Cage } \mathbf{1}\text{(aq)}]}{dt} = -k_{\mathrm{C}}[\textit{Cis}\text{-FAB(I)}]\left(\frac{[Cage\ \mathbf{1}(aq)]}{V_{ratio}}\right) - k_{\mathrm{C}}[\textit{Cis}\text{-FAB(II)}]\left(\frac{[Cage\ \mathbf{1}(aq)]}{V_{ratio}}\right) - k_{\mathrm{T}}[\textit{Trans}\text{-FAB(I)}]\left(\frac{[Cage\ \mathbf{1}(aq)]}{V_{ratio}}\right) - k_{\mathrm{T}}[\textit{Trans}\text{-FAB(II)}]\left(\frac{[Cage\ \mathbf{1}(aq)]}{V_{ratio}}\right) + 2k_{-\mathrm{C}}\frac{[\textit{Cis}\text{-FAB} \subset \text{Cage } \mathbf{1}\text{(aq)}]}{V_{ratio}} + 2k_{-\mathrm{T}}\frac{[\textit{Trans}\text{-FAB} \subset \text{Cage } \mathbf{1}\text{(aq)}]}{V_{ratio}}$

Eq. 16: $[FAB]_{total} = [\textit{Cis}\text{-FAB(I)}] + [\textit{Cis}\text{-FAB(II)}] + [\textit{Trans}\text{-FAB(I)}] + [\textit{Trans}\text{-FAB(II)}] + \frac{[\textit{Cis}\text{-FAB} \subset \text{Cage } \mathbf{1}\text{(aq)}]}{V_{ratio}} + \frac{[\textit{Trans}\text{-FAB} \subset \text{Cage } \mathbf{1}\text{(aq)}]}{V_{ratio}}$

Eq. 17: $[Cage\ \mathbf{1}]_{total} = [\textit{Cis}\text{-FAB} \subset \text{Cage } \mathbf{1}\text{(aq)}] + [\textit{Trans}\text{-FAB} \subset \text{Cage } \mathbf{1}\text{(aq)}] + [Cage\ \mathbf{1}(aq)]$

For the reverse transport:

Eq. 18: $\frac{d[\textit{Cis}\text{-FAB(I)}]}{dt} = -k_{\mathrm{C}}[\textit{Cis}\text{-FAB(I)}]\left(\frac{[Cage\ \mathbf{1}(aq)]}{V_{ratio}}\right) + k_{-\mathrm{C}}\frac{[\textit{Cis}\text{-FAB} \subset \text{Cage } \mathbf{1}\text{(aq)}]}{V_{ratio}} + k_{iTC,400nm}[\textit{Trans}\text{-FAB(I)}] - k_{iCT,400nm}[\textit{Cis}\text{-FAB(I)}]$

Eq. 19: $\frac{d[\textit{Trans}\text{-FAB(I)}]}{dt} = -k_{\mathrm{T}}[\textit{Trans}\text{-FAB(I)}]\left(\frac{[Cage\ \mathbf{1}(aq)]}{V_{ratio}}\right) + k_{-\mathrm{T}}\frac{[\textit{Trans}\text{-FAB} \subset \text{Cage } \mathbf{1}\text{(aq)}]}{V_{ratio}} - k_{iTC,400nm}[\textit{Trans}\text{-FAB(I)}] + k_{iCT,400nm}[\textit{Cis}\text{-FAB(I)}]$

Eq. 20: $\frac{d[\textit{Cis}\text{-FAB(II)}]}{dt} = -k_{\mathrm{C}}[\textit{Cis}\text{-FAB(II)}]\left(\frac{[Cage\ \mathbf{1}(aq)]}{V_{ratio}}\right) + k_{-\mathrm{C}}\frac{[\textit{Cis}\text{-FAB} \subset \text{Cage } \mathbf{1}\text{(aq)}]}{V_{ratio}} + k_{iTC,530nm}[\textit{Trans}\text{-FAB(II)}] - k_{iCT,530nm}[\textit{Cis}\text{-FAB(II)}]$

Eq. 21: $\frac{d[\textit{Trans}\text{-FAB(II)}]}{dt} = -k_{\mathrm{T}}[\textit{Trans}\text{-FAB(II)}]\left(\frac{[Cage\ \mathbf{1}(aq)]}{V_{ratio}}\right) + k_{-\mathrm{T}}\frac{[\textit{Trans}\text{-FAB} \subset \text{Cage } \mathbf{1}\text{(aq)}]}{V_{ratio}} - k_{iTC,530nm}[\textit{Trans}\text{-FAB(II)}] + k_{iCT,530nm}[\textit{Cis}\text{-FAB(II)}]$

Eq. 22: $\frac{d[\textit{Cis}\text{-FAB} \subset \text{Cage } \mathbf{1}\text{(aq)}]}{dt} = k_{\mathrm{C}}[\textit{Cis}\text{-FAB(I)}]\left(\frac{[Cage\ \mathbf{1}(aq)]}{V_{ratio}}\right) + k_{\mathrm{C}}[\textit{Cis}\text{-FAB(II)}]\left(\frac{[Cage\ \mathbf{1}(aq)]}{V_{ratio}}\right) - 2k_{-\mathrm{C}}\frac{[\textit{Cis}\text{-FAB} \subset \text{Cage } \mathbf{1}\text{(aq)}]}{V_{ratio}}$

Eq. 23: $\frac{d[\textit{Trans}\text{-FAB} \subset \text{Cage } \mathbf{1}\text{(aq)}]}{dt} = k_{\mathrm{T}}[\textit{Trans}\text{-FAB(I)}]\left(\frac{[Cage\ \mathbf{1}(aq)]}{V_{ratio}}\right) + k_{\mathrm{T}}[\textit{Trans}\text{-FAB(II)}]\left(\frac{[Cage\ \mathbf{1}(aq)]}{V_{ratio}}\right) - 2k_{-\mathrm{T}}\frac{[\textit{Trans}\text{-FAB} \subset \text{Cage } \mathbf{1}\text{(aq)}]}{V_{ratio}}$

Eq. 24: $\frac{d[\text{Cage } \mathbf{1}\text{(aq)}]}{dt} = -k_{\mathrm{C}}[\textit{Cis}\text{-FAB(I)}]\left(\frac{[Cage\ \mathbf{1}(aq)]}{V_{ratio}}\right) - k_{\mathrm{C}}[\textit{Cis}\text{-FAB(II)}]\left(\frac{[Cage\ \mathbf{1}(aq)]}{V_{ratio}}\right) - k_{\mathrm{T}}[\textit{Trans}\text{-FAB(I)}]\left(\frac{[Cage\ \mathbf{1}(aq)]}{V_{ratio}}\right) - k_{\mathrm{T}}[\textit{Trans}\text{-FAB(II)}]\left(\frac{[Cage\ \mathbf{1}(aq)]}{V_{ratio}}\right) + 2k_{-\mathrm{C}}\frac{[\textit{Cis}\text{-FAB} \subset \text{Cage } \mathbf{1}\text{(aq)}]}{V_{ratio}} + 2k_{-\mathrm{T}}\frac{[\textit{Trans}\text{-FAB} \subset \text{Cage } \mathbf{1}\text{(aq)}]}{V_{ratio}}$

Eq. 25: $[FAB]_{total} = [\textit{Cis}\text{-FAB(I)}] + [\textit{Cis}\text{-FAB(II)}] + [\textit{Trans}\text{-FAB(I)}] + [\textit{Trans}\text{-FAB(II)}] + \frac{[\textit{Cis}\text{-FAB} \subset \text{Cage } \mathbf{1}\text{(aq)}]}{V_{ratio}} + \frac{[\textit{Trans}\text{-FAB} \subset \text{Cage } \mathbf{1}\text{(aq)}]}{V_{ratio}}$

Eq. 26: $[Cage\ \mathbf{1}]_{total} = [\textit{Cis}\text{-FAB} \subset \text{Cage } \mathbf{1}\text{(aq)}] + [\textit{Trans}\text{-FAB} \subset \text{Cage } \mathbf{1}\text{(aq)}] + [Cage\ \mathbf{1}(aq)]$

Here, $k_{iCT(I)}$, $k_{iTC(I)}$, $k_{iCT(II)}$ and $k_{iTC(II)}$ as shown by the model in Supplementary Fig. 22 for the forward transport are corresponded to the values of $k_{iCT,530nm}$, $k_{iTC,530nm}$, $k_{iCT,400nm}$ and $k_{iTC,400nm}$ from Supplementary Fig. 5, respectively, as arm I was irradiated with the light at 530 nm and arm II was irradiated with the light at 400 nm in the forward transport experiment. However, $k_{iCT(I)}$, $k_{iTC(I)}$, $k_{iCT(II)}$ and $k_{iTC(II)}$ for the reverse transport are corresponded to the values of $k_{iCT,400nm}$, $k_{iTC,400nm}$, $k_{iCT,530nm}$ and $k_{iTC,530nm}$ from Supplementary Fig. 5, respectively, as arm I was irradiated with the light at 400 nm and arm II was irradiated with the light at 530 nm in the reverse transport experiment.

For each term in the Eq. 9-16 and Eq. 18-25 that contain the species in the aqueous phase, the factor V_{ratio} is required to obtain the relative concentration in the organic (dodecane) phase. V_{ratio} (= 0.8) can be calculated by dividing the volume of the solution in the organic phase (2.0 mL) by the volume of the solution in the aqueous phase (2.5 mL).

Unfortunately, the model is too complicated to be solved analytically due to the presence of the second-order terms depending on concentrations of two different species (guest uptake rates). Mathematica is not able to provide a general solution to the equations. However, resorting to numerical methods allowed us to obtain the time-dependence of concentrations graphically.

The built-in Mathematica functions for fitting could not be performed due to the complexity of the model. To fit the data, the least-square method was used instead by applying the code in Supplementary Fig. 20. With this method, the optimized rate constants obtained for *cis*- FAB were k_C = 0.42 mM^{-1}day^{-1} and k_{-C} = 1.69 day^{-1} and for *trans*-FAB were k_T = 0.29 mM^{-1}day^{-1} and k_{-T} = 1.23 day^{-1}, respectively. The fitting is shown in Supplementary Fig. 25.

```
(*Data can be imported from a csv file. Here we define functions recording the values to show the details of the NMR measurements*)
datatimef = {0.`, 0.5`, 2.`, 5.`, 10.`, 15.`, 20.`, 24.`};
datatimeb = {27.`, 30.`, 33.`, 36.`, 39.`, 44.`};
datatime = {0.`, 0.5`, 2.`, 5.`, 10.`, 15.`, 20.`, 24.`, 27.`, 30.`, 33.`, 36.`, 39.`, 44.`};
dataGcisI = {1.03575`, 7.4263`, 7.637`, 7.5535`, 7.3603`, 7.1502`, 7.1821`, 7.24775`, 0.96215`, 1.06565`, 1.13105`, 1.095`, 0.9852`, 1.04685`};
dataGtransI = {9.29485`, 1.6024`, 0.9183`, 0.7145`, 0.7749`, 0.7902`, 0.76475`, 0.7961`, 8.96095`, 9.49685`, 9.40105`, 9.61735`, 9.69745`, 9.769`};
dataGcisII = {1.07235`, 1.05295`, 0.9586`, 0.8913`, 0.98825`, 0.94475`, 1.0579`, 0.96935`, 7.82615`, 7.4713`, 7.0753`, 6.8877`, 6.95885`, 7.0183`};
dataGtransII = {8.97605`, 8.91025`, 8.9571`, 9.1237`, 9.57205`, 9.7362`, 9.6612`, 9.70135`, 0.84185`, 0.82195`, 0.92845`, 0.8691`, 0.70115`, 0.72985`};
eerrordataGcisI = {.089, .596, .612, .606, .590, .574, .577, .582, .084, .092, .097, .095, .086, .091};
errordataGtransI = {.743, .134, .080, .064, .069, .070, .068, .071, .717, .760, .752, .768, .775, .781};
errordataGcisII = {.092, .091, .084, .078, .086, .082, .091, .084, .628, .600, .568, .554, .559, .564};
errordataGtransII = {.718, .713, .717, .730, .765, .779, .773, .776, .074, .072, .081, .076, .063, .065};
datatimeGcisI = {};datatimeGtransI = {}; datatimeGcisII = {};datatimeGtransII = {}; datatimeIsum = {}; datatimeIIsum = {}; datatimediff = {};
For[i = 1, i <= Length[datatime], i++, AppendTo[datatimeGcisI, List[datatime[[i]], dataGcisI[[i]] \[PlusMinus] errordataGcisI[[i]]]]];
For[i = 1, i <= Length[datatime], i++, AppendTo[datatimeGtransI, List[datatime[[i]], dataGtransI[[i]] \[PlusMinus] errordataGtransI[[i]]]]];
For[i = 1, i <= Length[datatime], i++, AppendTo[datatimeGcisII, List[datatime[[i]], dataGcisII[[i]] \[PlusMinus] errordataGcisII[[i]]]]];
For[i = 1, i <= Length[datatime], i++, AppendTo[datatimeGtransII, List[datatime[[i]], dataGtransII[[i]] \[PlusMinus] errordataGtransII[[i]]]]];
For[i = 1, i <= Length[datatime], i++, AppendTo[datatimeIsum, List[datatime[[i]], (dataGcisI[[i]] + dataGtransI[[i]]) \[PlusMinus] Sqrt[errordataGcisI[[i]]^2 + errordataGtransI[[i]]^2]]]];
For[i = 1, i <= Length[datatime], i++, AppendTo[datatimeIIsum, List[datatime[[i]], (dataGcisII[[i]] + dataGtransII[[i]]) \[PlusMinus] Sqrt[errordataGcisII[[i]]^2 + errordataGtransII[[i]]^2]]]];
For[i = 1, i <= Length[datatime], i++, AppendTo[datatimediff, List[datatime[[i]], (dataGcisI[[i]] + dataGtransI[[i]] - dataGcisII[[i]] - dataGtransII[[i]]) \[PlusMinus] Sqrt[errordataGcisI[[i]]^2 +
errordataGtransI[[i]]^2 + errordataGcisII[[i]]^2 + errordataGtransII[[i]]^2]]]];
datatGcisI = Transpose@{datatime, dataGcisI}; datatGtransI = Transpose@{datatime, dataGtransI};
datatGcisII = Transpose@{datatime, dataGcisII}; datatGtransII = Transpose@{datatime, dataGtransII};

(*Plotting the experimental datapoint with errors*)
plt2 = ListPlot[datatimeGcisI, PlotStyle -> RGBColor[0, 0.59, 0.3], PlotMarkers -> {"OpenMarkers", Offset[10]}];
plt3 = ListPlot[datatimeGtransI, PlotStyle -> RGBColor[0.27, 0, 1], PlotMarkers -> {"OpenMarkers", Offset[10]}];
plt4 = ListPlot[datatimeGcisII, PlotStyle -> RGBColor[0, 0.59, 0.3]];
plt5 = ListPlot[datatimeGtransII, PlotStyle -> RGBColor[0.27, 0, 1]];

(*Defining rate constants, from optimizing the model and isomerization experiments*)
kf1 = 0.42; kb1 = 1.69; kf2 = 0.29; kb2 = 1.23; kTtCI = 1750.205; kCtTI = 111.7152; kTtCII = 437.184; kCtTII = 5027.616; Vratio = 0.8; totalconcMD = 21.45; totcage = 4;

(*Writing down differential equations and mass balance equations for the forward transport*)
sforwardnew = NDSolve[{
cisI'[t] == -kf1 cisI[t] (Ccaget[t]/Vratio) + kb1 (ciscage[t]/Vratio) + kTtCI transI[t] - kCtTI cisI[t],
transI'[t] == -kf2 transI[t] (Ccaget[t]/Vratio) + kb2 (transcage[t]/Vratio) - kTtCI transI[t] + kCtTI cisI[t] ,
cisII'[t] == -kf1 cisII[t] (Ccaget[t]/Vratio) + kb1 (ciscage[t]/Vratio) + kTtCII transII[t] - kCtTII cisII[t] ,
transII'[t] == -kf2 transII[t] (Ccaget[t]/Vratio) + kb2 (transcage[t]/Vratio) - kTtCII transII[t] + kCtTII cisII[t] ,
ciscage'[t] == kf1 cisI[t] Ccaget[t] - 2 kb1 ciscage[t] + kf1 cisII[t] Ccaget[t] ,
totalconcMD == cisI[t] + cisII[t] + ciscage[t]/Vratio + transI[t] + transII[t] + transcage[t]/Vratio,
totcage == Ccaget[t] + ciscage[t] + transcage[t],
cisI[0] == 0.051*totalconcMD, transI[0] == 0.455*totalconcMD, cisII[0] == 0.054*totalconcMD, transII[0] == 0.440*totalconcMD, ciscage[0] == 0, transcage[0] == 0, Ccaget[0] ==
totcage},
{cisI[t], transI[t], cisII[t], transII[t], ciscage[t], transcage[t], Ccaget[t]}, {t, 0, 25.}];
CI[t] = cisI[t] /. sforwardnew; CII[t] = cisII[t] /. sforwardnew; TI[t] = transI[t] /. sforwardnew; TII[t] = transII[t] /. sforwardnew;
cageC[t] = ciscage[t] /. sforwardnew; cageT[t] = transcage[t] /. sforwardnew; cage0[t] = Ccaget[t] /. sforwardnew;
cisIvalue = CI[t] /. t -> 24.; cisIIvalue = CII[t] /. t -> 24.; transIvalue = TI[t] /. t -> 24.; transIIvalue = TII[t] /. t -> 24.;
cageCvalue = cageC[t] /. t -> 24.; cageTvalue = cageT[t] /. t -> 24.; cage0value = cage0[t] /. t -> 24.;

(*Writing down differential equations and mass balance equations for the reverse transport*)
sbackward = NDSolve[{
cisI'[t] == -kf1 cisI[t] (Ccaget[t]/Vratio) + kb1 (ciscage[t]/Vratio) + kTtCII transI[t] - kCtTII cisI[t],
transI'[t] == -kf2 transI[t] (Ccaget[t]/Vratio) + kb2 (transcage[t]/Vratio) - kTtCII transI[t] + kCtTII cisI[t],
cisII'[t] == -kf1 cisII[t] (Ccaget[t]/Vratio) + kb1 (ciscage[t]/Vratio) + kTtCI transII[t] - kCtTI cisII[t],
transII'[t] == -kf2 transII[t] (Ccaget[t]/Vratio) + kb2 (transcage[t]/Vratio) - kTtCI transII[t] + kCtTI cisII[t],
ciscage'[t] == kf1 cisI[t] Ccaget[t] - 2 kb1 ciscage[t] + kf1 cisII[t] Ccaget[t],
cisIvalue[[1]] + cisIIvalue[[1]] + cageCvalue[[1]]/Vratio + transIvalue[[1]] + transIIvalue[[1]] + cageTvalue[[1]]/Vratio == cisI[t] + cisII[t] + ciscage[t]/Vratio + transI[t] + transII[t] +
transcage[t]/Vratio,
cageCvalue[[1]] + cageTvalue[[1]] + cage0value[[1]] == Ccaget[t] + ciscage[t] + transcage[t],
cisI[24.] == cisIvalue[[1]], transI[24.] == transIvalue[[1]], cisII[24.] == cisIIvalue[[1]], transII[24.] == transIIvalue[[1]], ciscage[24.] == cageCvalue[[1]], transcage[24.] == cageTvalue[[1]],
Ccaget[24.] == cage0value[[1]]},
{cisI[t], transI[t], cisII[t], transII[t], ciscage[t], transcage[t], Ccaget[t]}, {t, 24., 44}, AccuracyGoal -> 6, PrecisionGoal -> 6];

(*Plotting the model prediction for each isomer*)
pltf = Plot[Evaluate[{cisI[t], transI[t], cisII[t], transII[t]} /.sforwardnew], {t, 0, 24}, PlotLegends -> {GcisA[t], GtransA[t], GcisB[t], GtransB[t]}, PlotStyle -> {{RGBColor[0, 0.59, 0.3],
Dashed, Thick}, {RGBColor[0.27, 0, 1], Dashed, Thick}, {RGBColor[0, 0.59, 0.3]}, {RGBColor[0.27, 0., 1]}}];
pltb = Plot[Evaluate[{cisI[t], transI[t], cisII[t], transII[t]} /.sbackward], {t, 24, 44}, PlotStyle -> {{RGBColor[0, 0.59, 0.3], Dashed, Thick}, {RGBColor[0.27, 0, 1], Dashed, Thick},
{RGBColor[0, 0.59, 0.3]}, {RGBColor[0.27, 0., 1]}}];

(*Plotting the model prediction for total FAB concentration*)
pltfsum = Plot[Evaluate[{cisI[t] + transI[t], cisII[t] + transII[t]} /.sforwardnew], {t, 0, 24}, PlotLegends -> {"Arm I", "Arm II"}, PlotStyle -> {{Gray}, {Black}}];
pltbsum = Plot[Evaluate[{cisI[t] + transI[t], cisII[t] + transII[t]} /.sbackward], {t, 24, 44}, PlotStyle -> {{Gray}, {Black}}];
plt2sum = ListPlot[datatimeIsum, PlotStyle -> Gray];
plt3sum = ListPlot[datatimeIIsum, PlotStyle -> Black];

(*Plotting the model prediction for the aqueous layer*)
pltfcage = Plot[Evaluate[{ciscage[t], transcage[t], Ccaget[t], ciscage[t] + transcage[t] + Ccaget[t]} /. sforwardnew], {t, 0, 24}, PlotLegends -> {ciscage[t], transcage[t], emptycage[t],
totalcage}, PlotStyle -> {{RGBColor[0, 0.59, 0.3], Thick}, {RGBColor[0.27, 0., 1], Thick}, {Black, Dashed, Thick}, {Black, Thick}}];
pltbcage = Plot[Evaluate[{ciscage[t], transcage[t], Ccaget[t], ciscage[t] + transcage[t] + Ccaget[t]} /. sbackward], {t, 24, 44}, PlotStyle -> {{RGBColor[0, 0.59, 0.3], Thick},
{RGBColor[0.27, 0., 1], Thick}, {Black, Dashed, Thick}, {Black, Thick}}];

(*Showing the plots*)
Show[pltf, pltb, plt2, plt3, plt4, plt5, PlotRange -> {{0, 44}, {0, 11}}, AxesLabel -> {"Time / days", "Concentration / mM"}, LabelStyle -> Directive[Black, Bold]]
Show[pltfsum, pltbsum, plt2sum, plt3sum, PlotRange -> {{0, 44}, {0, 14}}, AxesLabel -> {"Time / days", "Concentration / mM"}, AxesOrigin -> {0, 0}]
Show[pltfcage, pltbcage, PlotRange -> {{0, 44}, {0, 4.}}, AxesLabel -> {"Time / days", "Concentration / mM"}, AxesOrigin -> {0, 0}, LabelStyle -> Directive[Black, Bold]]
```

Supplementary Fig. 23. Mathematica code for System 1, showing the differential equations and mass balance equations defining the system according to the kinetic model in Fig. 2e.

```
datafitMDdatafbtot = {};
kTtCI = 1750.205;
kCtTI = 111.7152;
kTtCII = 437.184;
kCtTII = 5027.616;
kDCTc = 0;
kDTCc = 0;
Vratio = 0.8;
totalconcMD = 21.45;
totcage = 4;
For[ kf1 = 0.50001, kf1 ≤ 0.52, kf1 += 0.002,
  For[ kb1 = 0.80001, kb1 ≤ 1.05, kb1 += 0.05,
   For[ kf2 = 0.3401, kf2 ≤ 0.365, kf2 += 0.005,
    For[ kb2 = 17.8001, kb2 ≤ 19.1, kb2 += 0.3;
     sforwardnew = NDSolve[{cisI'[t] == -kf1 cisI[t] (Ccaget[t] / Vratio) + kb1 (ciscage[t] / Vratio) + kTtCI transI[t] - kCtTI cisI[t] - kDCTc cisI[t] (transcage[t] / Vratio) + kDTCc transI[t] (ciscage[t] / Vratio),
        transI'[t] == -kf2 transI[t] (Ccaget[t] / Vratio) + kb2 (transcage[t] / Vratio) - kTtCI transI[t] + kCtTI cisI[t] + kDCTc cisI[t] (transcage[t] / Vratio) - kDTCc transI[t] (ciscage[t] / Vratio),
        cisII'[t] == -kf1 cisII[t] (Ccaget[t] / Vratio) + kb1 (ciscage[t] / Vratio) + kTtCII transII[t] - kCtTII cisII[t] - kDCTc cisII[t] (transcage[t] / Vratio) + kDTCc transII[t] (ciscage[t] / Vratio),
        transII'[t] == -kf2 transII[t] (Ccaget[t] / Vratio) + kb2 (transcage[t] / Vratio) - kTtCII transII[t] + kCtTII cisII[t] + kDCTc cisII[t] (transcage[t] / Vratio) - kDTCc transII[t] (ciscage[t] / Vratio),
        ciscage'[t] == kf1 cisI[t] × Ccaget[t] - 2 kb1 ciscage[t] + kf1 cisII[t] × Ccaget[t] + kDCTc cisI[t] × transcage[t] - kDTCc transI[t] × ciscage[t] + kDCTc cisII[t] × transcage[t] - kDTCc transII[t] × ciscage[t],
        totalconcMD == cisI[t] + cisII[t] + ciscage[t] / Vratio + transI[t] + transII[t] + transcage[t] / Vratio,
        totcage == Ccaget[t] + ciscage[t] + transcage[t],
        cisI[0] == 0.051 * totalconcMD, transI[0] == 0.455 * totalconcMD, cisII[0] == 0.054 * totalconcMD, transII[0] == 0.440 * totalconcMD, ciscage[0] == 0, transcage[0] == 0, Ccaget[0] == totcage},
       {cisI[t], transI[t], cisII[t], transII[t], ciscage[t], transcage[t], Ccaget[t]}, {t, 0, 25.}];
     CI[t] = cisI[t] /. sforwardnew;
     CII[t] = cisII[t] /. sforwardnew;
     TI[t] = transI[t] /. sforwardnew;
     TII[t] = transII[t] /. sforwardnew;
     cageC[t] = ciscage[t] /. sforwardnew;
     cageT[t] = transcage[t] /. sforwardnew;
     cage0[t] = Ccaget[t] /. sforwardnew;
     cisIvalue = CI[t] /. t → 24.;
     cisIIvalue = CII[t] /. t → 24.;
     transIvalue = TI[t] /. t → 24.;
     transIIvalue = TII[t] /. t → 24.;
     cageCvalue = cageC[t] /. t → 24.;
     cageTvalue = cageT[t] /. t → 24.;
     cage0value = cage0[t] /. t → 24.;
     sbackward = NDSolve[{cisI'[t] == -kf1 cisI[t] (Ccaget[t] / Vratio) + kb1 (ciscage[t] / Vratio) + kTtCII transI[t] - kCtTII cisI[t] - kDCTc cisI[t] (transcage[t] / Vratio) + kDTCc transI[t] (ciscage[t] / Vratio),
        transI'[t] == -kf2 transI[t] (Ccaget[t] / Vratio) + kb2 (transcage[t] / Vratio) - kTtCII transI[t] + kCtTII cisI[t] + kDCTc cisI[t] (transcage[t] / Vratio) - kDTCc transI[t] (ciscage[t] / Vratio),
        cisII'[t] == -kf1 cisII[t] (Ccaget[t] / Vratio) + kb1 (ciscage[t] / Vratio) + kTtCI transII[t] - kCtTI cisII[t] - kDCTc cisII[t] (transcage[t] / Vratio) + kDTCc transII[t] (ciscage[t] / Vratio),
        transII'[t] == -kf2 transII[t] (Ccaget[t] / Vratio) + kb2 (transcage[t] / Vratio) - kTtCI transII[t] + kCtTI cisII[t] + kDCTc cisII[t] (transcage[t] / Vratio) - kDTCc transII[t] (ciscage[t] / Vratio),
        ciscage'[t] == kf1 cisI[t] × Ccaget[t] - 2 kb1 ciscage[t] + kf1 cisII[t] × Ccaget[t] + kDCTc cisI[t] × transcage[t] - kDTCc transI[t] × ciscage[t] + kDCTc cisII[t] × transcage[t] - kDTCc transII[t] × ciscage[t],
        cisIvalue[[1]] + cisIIvalue[[1]] + cageCvalue[[1]] / Vratio + transIvalue[[1]] + transIIvalue[[1]] + cageTvalue[[1]] / Vratio == cisI[t] + cisII[t] + ciscage[t] / Vratio + transI[t] + transII[t] + transcage[t] / Vratio,
        cageCvalue[[1]] + cageTvalue[[1]] + cage0value[[1]] == Ccaget[t] + ciscage[t] + transcage[t],
        cisI[24.] == cisIvalue[[1]], transI[24.] == transIvalue[[1]], cisII[24.] == cisIIvalue[[1]], transII[24.] == transIIvalue[[1]], ciscage[24.] == cageCvalue[[1]], transcage[24.] == cageTvalue[[1]],
        Ccaget[24.] == cage0value[[1]]},
       {cisI[t], transI[t], cisII[t], transII[t], ciscage[t], transcage[t], Ccaget[t]}, {t, 24., 44}, AccuracyGoal → 6, PrecisionGoal → 6];
     cisAf[t] = cisI[t] /. sforwardnew;
     cisBf[t] = cisII[t] /. sforwardnew;
     transAf[t] = transI[t] /. sforwardnew;
     transBf[t] = transII[t] /. sforwardnew;
     cisAb[t] = cisI[t] /. sbackward;
     cisBb[t] = cisII[t] /. sbackward;
     transAb[t] = transI[t] /. sbackward;
     transBb[t] = transII[t] /. sbackward;
     concvaluescisAf = cisAf[t] /. t → datatimef;
     concvaluescisAb = cisAb[t] /. t → datatimeb;
     concvaluescisBf = cisBf[t] /. t → datatimef;
     concvaluescisBb = cisBb[t] /. t → datatimeb;
     concvaluestransAf = transAf[t] /. t → datatimef;
     concvaluestransAb = transAb[t] /. t → datatimeb;
     concvaluestransBf = transBf[t] /. t → datatimef;
     concvaluestransBb = transBb[t] /. t → datatimeb;
     difflistsq = (dataGcisI - Flatten[Join[concvaluescisAf, concvaluescisAb]]) ^ 2 + (dataGcisII - Flatten[Join[concvaluescisBf, concvaluescisBb]]) ^ 2 +
       (dataGtransI - Flatten[Join[concvaluestransAf, concvaluestransAb]]) ^ 2 + (dataGtransII - Flatten[Join[concvaluestransBf, concvaluestransBb]]) ^ 2;
     err = Total[difflistsq];
     AppendTo[datafitMDdatafbtot, List[kf1, kb1, kf2, kb2, Sqrt[err / 41]]]]]]];
result = MinimalBy[datafitMDdatafbtot, Last]
```

Supplementary Fig. 24. Mathematica code for optimisation of the fitting using the least-square method. Due to the complexity of the system, ranges of each rate constant k_C, k_{-C}, k_T and k_{-T} for guest uptake and release of *cis*- FAB and *trans*- FAB were screened starting with larger increment before narrowing down to 2 decimal place accuracy. A set of the rate constants that provided minimum error was used for the fitting with the experimental data ($k_C = 0.42$ mM^{-1}day^{-1}, $k_{-C} = 1.69$ day^{-1}, $k_T = 0.29$ mM^{-1}day^{-1} and $k_{-T} = 1.23$ day^{-1}).

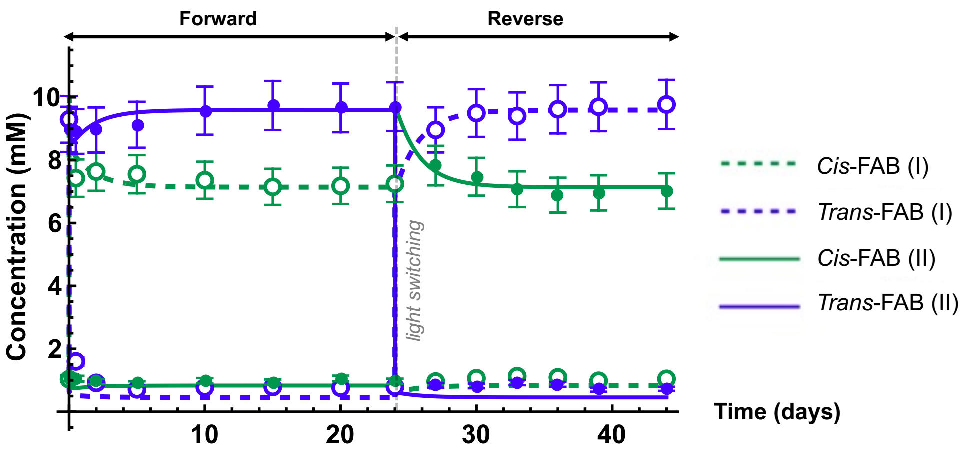

Supplementary Fig. 25. Concentrations of *cis*-FAB (green) and *trans*-FAB (blue) in arm I (hollow dots and dashed line) and arm II (solid dots and solid line) during the forward and reverse transport in System 1, showing experimental results measured by ^{1}H NMR (dots), error bars and model predictions (lines) for each arm. In the forward transport, *cis*-FAB was observed to flow from arm I to arm II where it was isomerized to *trans*-FAB. Upon switching the light stimuli in the reverse transport, the flow of *cis*-FAB was reversed (from arm II to arm I) and the subsequent isomerization to *trans*-FAB was happening in arm I. These processes caused the shift in the total FAB concentrations. The grey dashed line indicates the point which the LED light strips were swapped, switching from forward to reverse transport. Data are presented as mean values +/- measurement errors, derived from error propagation of the SD and the signal-to-noise ratio (n=22) of coronene (Supplementary Section 5). *This figure is also presented in the Extended Data section as Extended Data Fig. 2.*

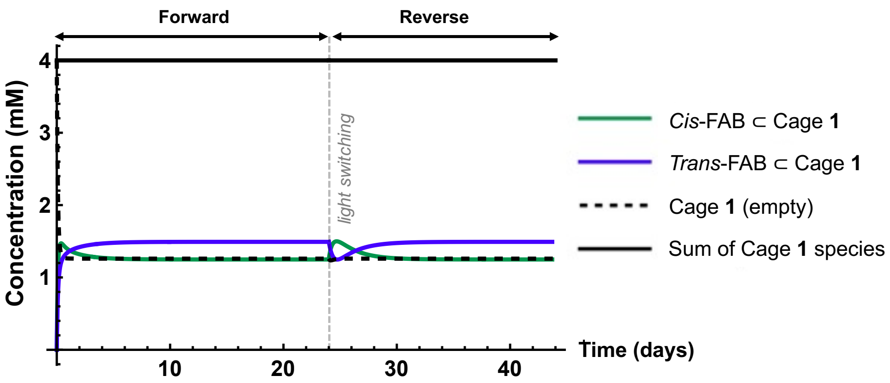

Supplementary Fig. 26. Model predictions of the concentrations of *cis*-FAB ⊂ Cage **1** (green solid line), *trans*-FAB ⊂ Cage **1** (blue solid line), Cage **1** with empty cavity (black dashed line) and total concentration of Cage **1** species (black solid line) in the aqueous layer during the forward and reverse transport for System 1. Initially, *cis*-FAB ⊂ Cage **1** was present in large quantity due to *cis*- FAB having faster guest uptake rate than *trans*- FAB. Interestingly, the concentration of *trans*- FAB ⊂ Cage **1** is higher than *cis*- FAB ⊂ Cage **1** at the steady state, although *cis*-FAB bound stronger in the cage (Supplementary Fig. 8). This is because multiple dependent processes including isomerization are present together in the system, and there is more *trans*-FAB present in the system overall. The grey dashed line indicates the point which the LED light strips were swapped, switching from forward to reverse transport.

11. Model prediction for Naphthalene Counterflow Augments Maxwell's Demon experiment (System 2), and Maxwell's Demon Drives Naphthalene Counterflow experiment (System 3)

In System 2 and 3, we initially assume that naphthalene affects the system by reducing the number of available cages for FAB transport, whereby only α *[Cage **1**]$_{initial}$ of Cage **1** is active ($0 \le \alpha \le 1$). With this assumption, the model predicts larger FAB concentration difference between the two arms at lower α at the steady state (Supplementary Fig. 27).

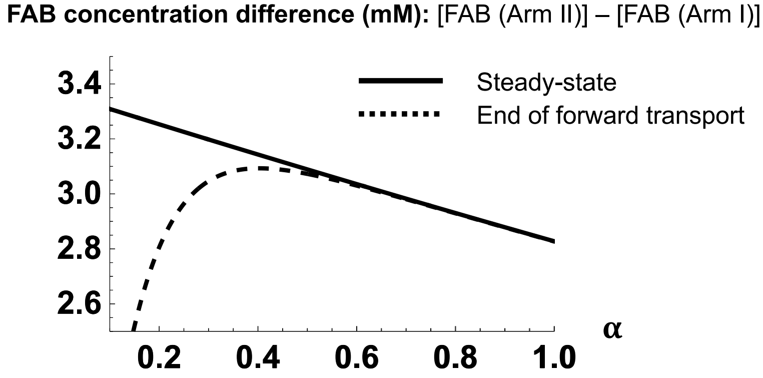

Supplementary Fig. 27. The graph showing the model predictions of FAB concentration differences as a function of α values, accounting for the presence of naphthalene in System 2 and 3. Only α *[Cage **1**]$_{initial}$ of Cage **1** is active in the system ($0 \le \alpha \le 1$). The prediction indicates larger FAB concentration difference between 2 arms at lower α at the steady-state (solid line). At the end of the forward transport (day 20 for System 2 and 3), the relationship is more complicated at lower α (dashed line), since lower α values mean that there are less cages available for transporting FAB across the aqueous layer, resulting in slower transport. Hence, it would take longer to reach steady state at lower α.

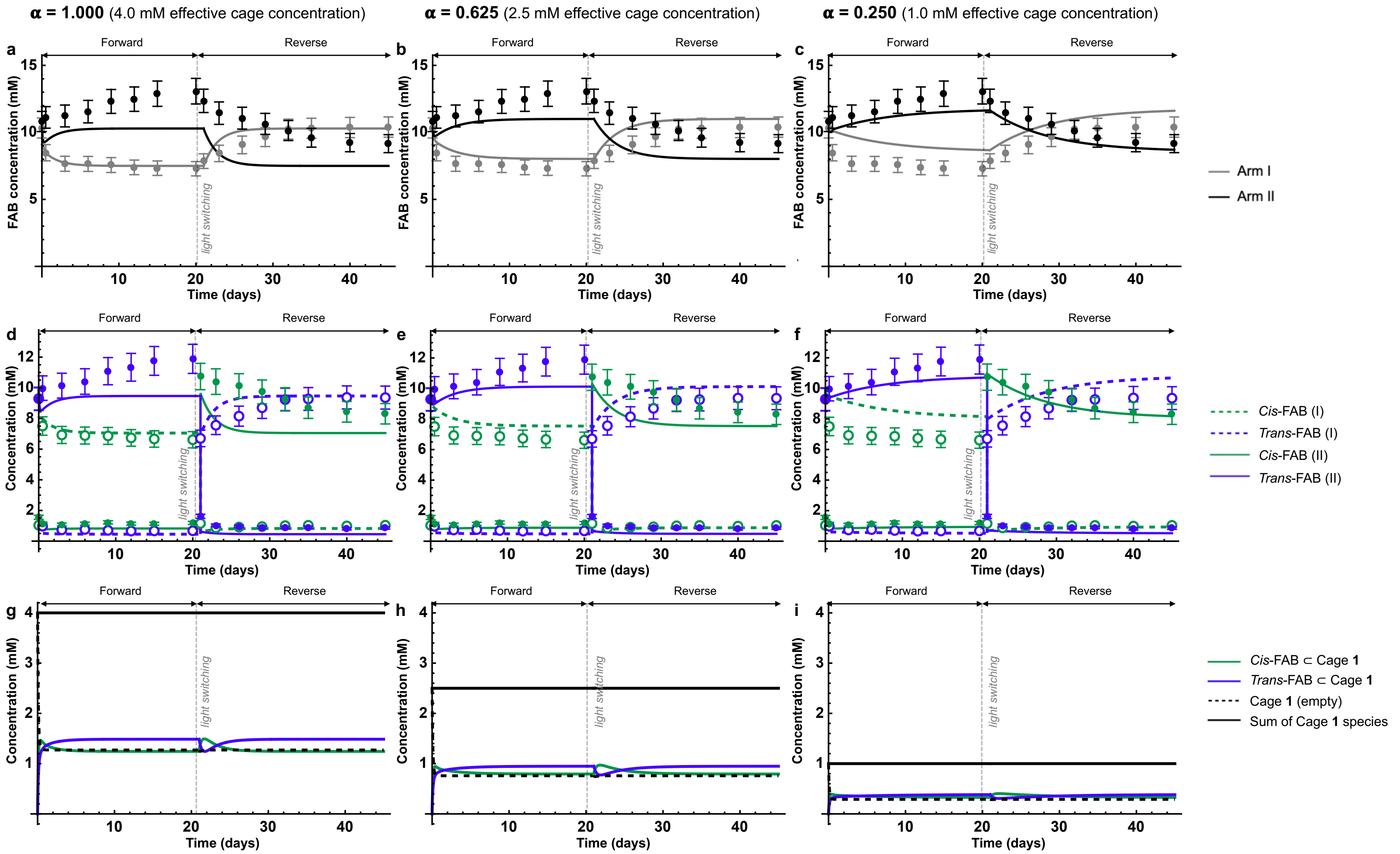

Supplementary Fig. 28. **a**, **b** and **c**, Sum of the *trans*- and *cis*-FAB concentration in arm I (grey) and arm II (black) during the forward and reverse transport for System 2, showing the experimental results measured by ^{1}H NMR (dots), error bars and model predictions (solid lines) for each arm. **d**, **e** and **f**, Concentrations of *cis*-FAB (green) and *trans*-FAB (blue) in arm I (hollow dots and dashed line) and arm II (solid dots and solid line) during the forward and reverse transport for System 2, showing the experimental results measured by ^{1}H NMR (dots), error bars and model predictions (lines) for

each arm. Data in **a-f** are presented as mean values +/- measurement errors, derived from error propagation of the SD and the signal-to-noise ratio (n=22) of coronene (Supplementary Section 5). **g**, **h** and **i**, Model predictions of the concentrations of *cis*-FAB ⊂ Cage **1** (green solid line), *trans*-FAB ⊂ Cage **1** (blue solid line), Cage **1** with empty cavity (black dashed line) and total concentration of Cage **1** species (black solid line) in the aqueous layer during the forward and reverse transport for System 2. Here, the assumption is made such that the presence of naphthalene reduces the amount of the cage available for FAB transport (effective cage concentration). The effective cage concentration was defined by α multiplied by the initial concentration of Cage **1**. The model predictions are provided with α values equal to 1.000 (**a, d** and **g**), 0.625 (**b, e** and **h**) and 0.250 (**c, f** and **i**). Following the finding in Supplementary Fig. 27, the prediction at α = 0.625 (**b** and **f**) provides a better fitting for the experimental data of the forward transport, showing a greater concentration difference compared to the system with α = 1.000 (**c**). However, the prediction at α = 0.250 (**c** and **f**) provides a qualitatively better fitting at the start of the reverse transport with the slow formation of the concentration difference between arm I and arm II. This is due to the slower FAB transportation as more naphthalene has been encapsulated, and so there is less FAB transported by the cage at any time. Therefore, it would take longer for the system to reach the steady-state prediction. We note that this assumption cannot account for the change in naphthalene concentration over time. Hence, the prediction using this simplification would only work at a steady state, which the system has not reached. Further refinement of the model is provided in the following section, where naphthalene transport over time is considered.

Improving the model further to account for naphthalene transport in System 2, we develop a model where independent naphthalene ingress and egress by Cage **1** are considered (Supplementary Fig. 29c). Putting this into the context of the α values, considering naphthalene transport allows α values to be adjusted as a function of time, depending on how much naphthalene has been encapsulated. This improves the fitting, especially for the initial period of the forward transport (Supplementary Fig. 29).

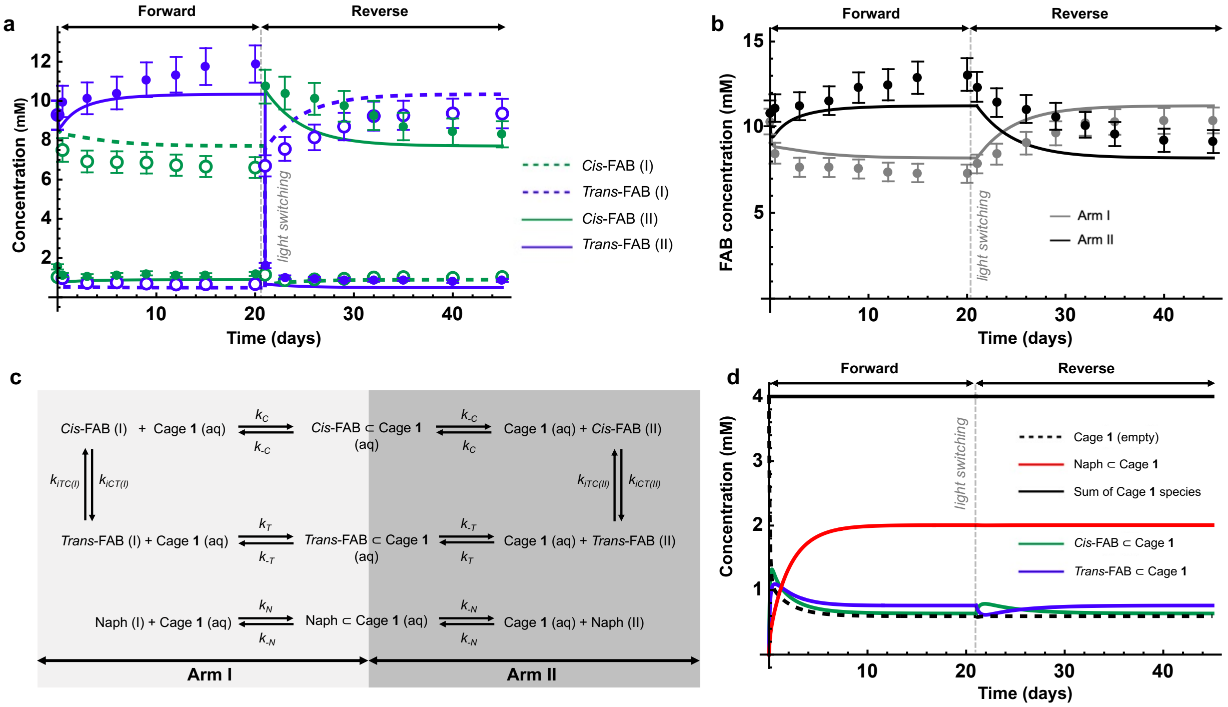

Supplementary Fig. 29. a, Concentrations of *cis*-FAB (green) and *trans*-FAB (blue) in arm I (hollow dots and dashed line) and arm II (solid dots and solid line) during the forward and reverse transport for System 2, showing experimental results measured by ^{1}H NMR (dots), error bars and model predictions (lines) for each arm for the model with the kinetics of naphthalene ingress and egress included. **b**, Sum of *trans*- and *cis*-FAB concentration in arm I (grey) and arm II (black) during the forward and reverse transport for System 2, showing the experimental results measured by ^{1}H NMR (dots), error bars and model predictions (solid lines) for each arm. **c**, The improved model including the kinetics of naphthalene ingress (rate constant k_N) and egress (rate constant k_{-N}). Here, the model prediction is based on the optimized values of $k_N = 0.075$ mM^{-1}day^{-1} and $k_{-N} = 0.100$ day^{-1}. **d**, Model predictions of the concentrations of *cis*-FAB ⊂ Cage **1** (green solid line), *trans*-FAB ⊂ Cage **1** (blue solid line), Cage **1** with empty cavity (black dashed line), naphthalene ⊂ Cage **1** (red solid line) and total concentration of Cage **1** species (black solid line) in the aqueous layer during the forward and reverse transport for System 2. Data in **a-b** are presented as mean values +/- measurement errors, derived from error propagation of the SD and the signal-to-noise ratio (n=22) of coronene (Supplementary Section 5).

However, the additional consideration of independent naphthalene ingress and egress by Cage 1 alone is not sufficient to account for the observations of naphthalene transport in System 2 (Supplementary Fig. 31a), where transport is faster than predicted. This observation provides further insight into the system, indicating the presence of other mechanisms that promote more rapid transport of naphthalene from arm I to the aqueous layer, and more rapid release from the aqueous layer to arm II. Furthermore, we observe a greater degree of overshooting of the naphthalene concentration in arm I during forward transport from day 12, something which the current model cannot predict.

To account for these details, a competitive displacement mechanism can be added to the model (Supplementary Fig. 30). This mechanism would allow one guest to displace another guest directly without requiring the cage to be empty first. Adding 6 new rate constants to the model results in more challenging optimization process. The values for the competitive displacement rate constants (k_{CN} = 0.1 day^{-1}, k_{NC} = 0.04 day^{-1}, k_{TN} = 0.05 day^{-1}, k_{NT} = 0.04 day^{-1}, k_{CT} = 0.01 day^{-1}, and k_{TC} = 0.01 day^{-1}) were selected to fit the experimental data qualitatively. These constants are used for both System 2 and 3 (Supplementary Fig. 31-33).

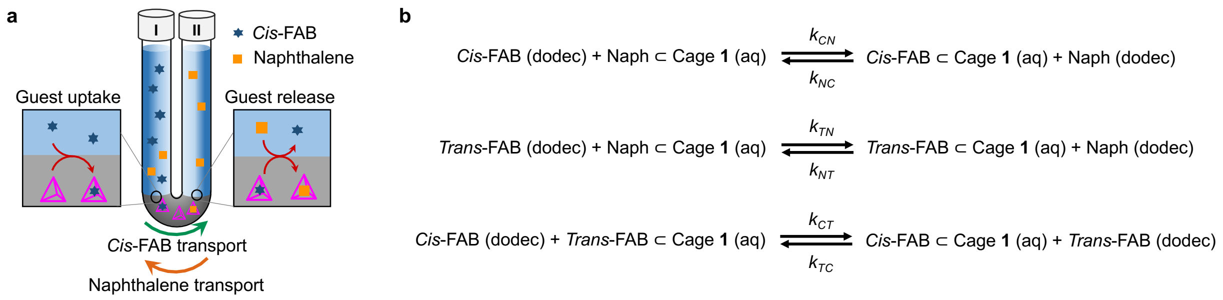

Supplementary Fig. 30. a, Illustration of guest uptake and release mechanisms triggered by naphthalene and *cis*-FAB competitive displacement at the dodecane/aqueous arm II interface. **b**, Chemical reactions showing competitive displacement mechanisms for each species presented in System 2 and 3, with their corresponding rate constants. The constants used in this study for both System 2 and 3 are k_{CN} = 0.1 day^{-1}, k_{NC} = 0.04 day^{-1}, k_{TN} = 0.05 day^{-1}, k_{NT} = 0.04 day^{-1}, k_{CT} = 0.01 day^{-1}, and k_{TC} = 0.01 day^{-1}, which align with our suggestion that *cis*-FAB displaces naphthalene ⊂ Cage **1** better than *trans*-FAB ($k_{CN} > k_{TN}$).

This competitive displacement mechanism model provides an improved explanation of naphthalene transport in System 2 (Supplementary Fig. 31b). The model is able to explain the shift in the concentration of naphthalene further away from equilibrium at the end of the forward and reverse transport. The utility of this model increases further in System 3, where the flow of FAB out of equilibrium was used to drive naphthalene counterflow (Supplementary Fig. 33c). Consideration of competitive displacement also predicts that naphthalene will be driven out of equilibrium.

The competitive displacement model thus predicts the qualitative behavior of the system when naphthalene is present. Considering naphthalene transport and competitive displacement add complexity to the system, leading to challenges in optimization. Our treatment using the Mathematica software package[6] was not able to fit the behavior of the system. Selection of the values of the kinetic constants is therefore based on qualitative fitting.

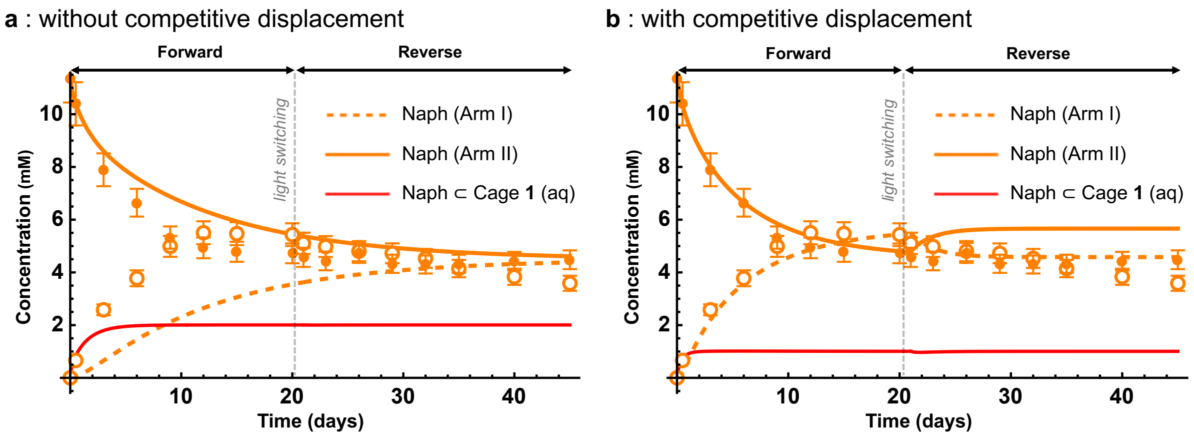

Supplementary Fig. 31. Concentrations of naphthalene measured by ^{1}H NMR in arm I (hollow orange dots) and arm II (solid orange dots), and the model predictions for the concentrations of naphthalene in arm I (orange dashed line), arm II (orange solid line) and naphthalene ⊂ Cage **1** (red solid line) during the forward and reverse transport for System 2, for the models without (**a**) and with (**b**) competitive displacement included. Data are presented as mean values +/- measurement errors, derived from error propagation of the SD and the signal-to-noise ratio (n=22) of coronene (Supplementary Section 5). Without competitive displacement, only independent guest ingress and egress of naphthalene were included in the model (k_N = 0.075 mM^{-1}day^{-1} and k_{-N} = 0.100 day^{-1}); the model is only accurate for the prediction at the steady-state condition at the end of the experiment; the model predicts slower transport of naphthalene from arm I to arm II compared to the experimental results. With competitive displacement included (k_{CN} = 0.1 day^{-1}, k_{NC} = 0.04 day^{-1}, k_{TN} = 0.05 day^{-1}, k_{NT} = 0.04 day^{-1}, k_{CT} = 0.01 day^{-1}, and k_{TC} = 0.01 day^{-1}), the model is able to predict the concentration of naphthalene at the initial stage, as well as the non-zero concentration gradient where the concentration of naphthalene in arm I overtakes that of arm II for the forward transport (day 12-20). Introducing 6 new rate constants to the model (Supplementary Fig. 30b) increases the difficulty of optimizing the process to fit data perfectly. The model with competitive displacement included is able to predict the non-zero concentration gradient behavior for the reverse transport, suggesting arm II has higher naphthalene concentration than arm I.

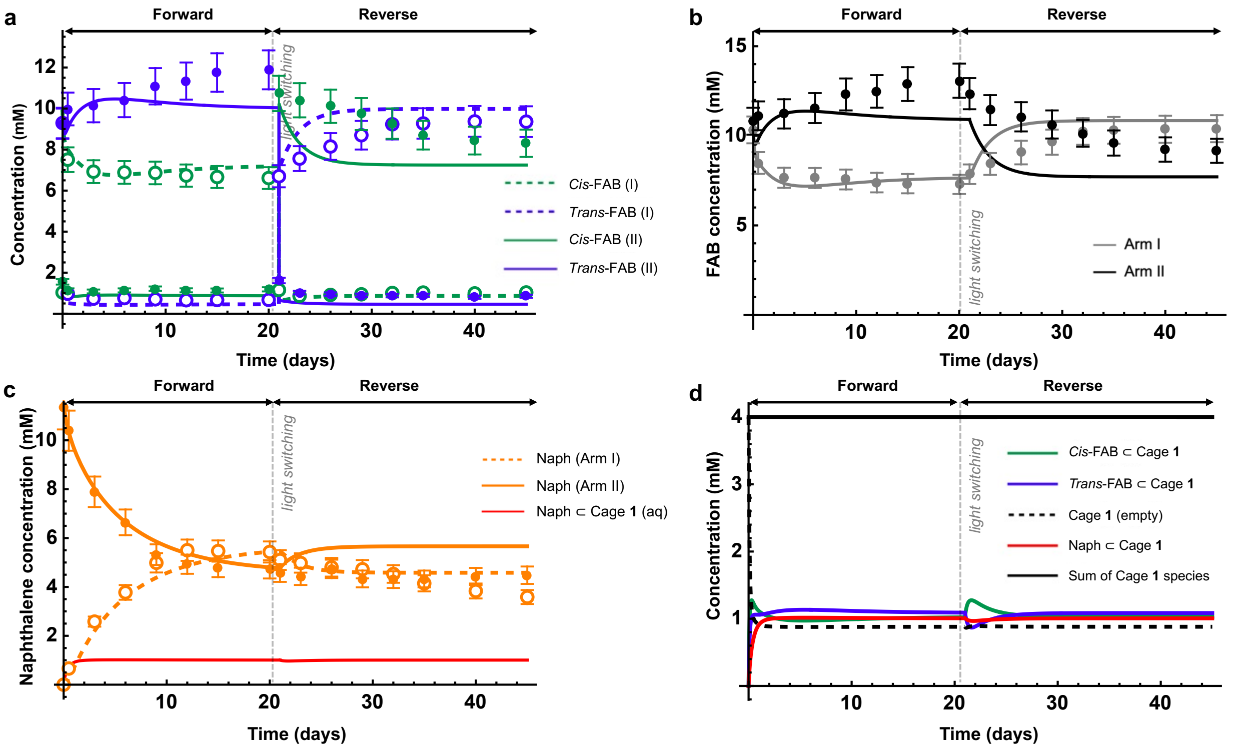

Supplementary Fig. 32. a, Concentrations of *cis*-FAB (green) and *trans*-FAB (blue) in arm I (hollow dots and dashed line) and arm II (solid dots and solid line) during the forward and reverse transport for System 2, showing the experimental results measured by ^{1}H NMR (dots), error bars and model predictions (lines) for each arm, for the model with competitive displacement included (as shown in Supplementary Fig. 30). The constants used here are k_{CN} = 0.1 day^{-1}, k_{NC} = 0.04 day^{-1}, k_{TN} = 0.05 day^{-1}, k_{NT} = 0.04 day^{-1}, k_{CT} = 0.01 day^{-1}, and k_{TC} = 0.01 day^{-1}. **b**, Model predictions of the concentrations of *cis*-FAB ⊂ Cage **1** (green solid line), *trans*-FAB ⊂ Cage **1** (blue solid line), Cage **1** with empty cavity (black dashed line), naphthalene ⊂ Cage **1** (red solid line) and total concentration of Cage **1** species (black solid line) in the aqueous layer during the forward and reverse transport for System 2. **c**, Concentrations of naphthalene measured by ^{1}H NMR in arm I (hollow orange dots) and arm II (solid orange dots), and the model predictions for the concentrations of naphthalene in arm I (orange dashed line), arm II (orange solid line) and naphthalene ⊂ Cage **1** (red solid line) during the forward and reverse transport for System 2. **d**, Model predictions of the concentrations of *cis*-FAB ⊂ Cage **1** (green solid line), *trans*-FAB ⊂ Cage **1** (blue solid line), Cage **1** with empty cavity (black dashed line), naphthalene ⊂ Cage **1** (red solid line) and total concentration of Cage **1** species (black solid line) in the aqueous layer during the forward and reverse transport for System 2. Data in **a-c** are presented as mean values +/- measurement errors, derived from error propagation of the SD and the signal-to-noise ratio (n=22) of coronene (Supplementary Section 5). The competitive displacement model thus predicts the qualitative behavior of the system when naphthalene is present. Considering naphthalene transport and competitive displacement add complexity to the system, leading to challenges in optimization. Our treatment using the Mathematica software package[6] was not able to fit the behavior of the system. Selection of the values of the kinetic constants is therefore based on qualitative fitting.

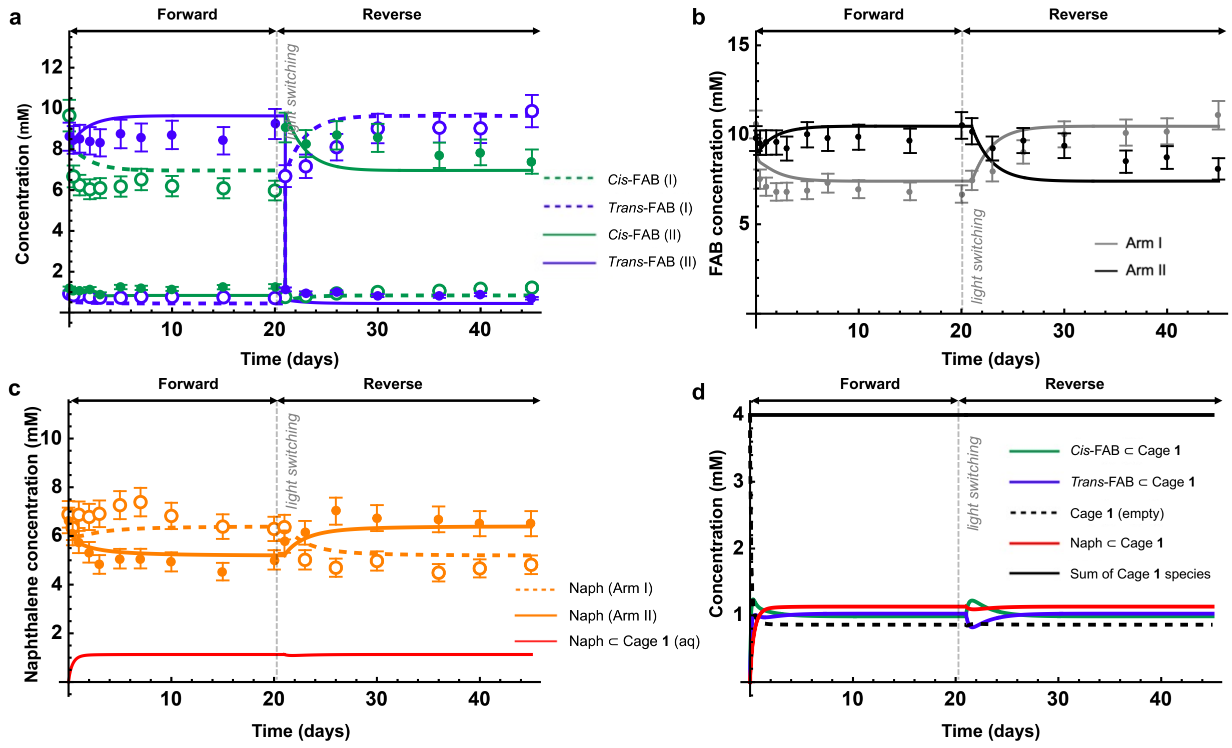

Supplementary Fig. 33. a, Concentrations of *cis*-FAB (green) and *trans*-FAB (blue) in arm I (hollow dots and dashed line) and arm II (solid dots and solid line) during the forward and reverse transport for System 3, showing the experimental results measured by ^{1}H NMR (dots), error bars and model predictions (lines) for each arm, for the model with competitive displacement included (as shown by Supplementary Fig. 30). The constants used here are k_{CN} = 0.1 day^{-1}, k_{NC} = 0.04 day^{-1}, k_{TN} = 0.05 day^{-1}, k_{NT} = 0.04 day^{-1}, k_{CT} = 0.01 day^{-1}, and k_{TC} = 0.01 day^{-1}. **b**, Sum of *trans*- and *cis*-FAB concentration in arm I (grey) and arm II (black) during forward and reverse transport for System 3, showing the experimental results measured by ^{1}H NMR (dots), error bars and model predictions (solid lines) for each arm. **c**, Concentrations of naphthalene measured by ^{1}H NMR in arm I (hollow orange dots) and arm II (solid orange dots), and the model predictions for the concentrations of naphthalene in arm I (orange dashed line), arm II (orange solid line) and naphthalene ⊂ Cage **1** (red solid line) during the forward and reverse transport for System 3. **d**, Model predictions of the concentrations of *cis*-FAB ⊂ Cage **1** (green solid line), *trans*-FAB ⊂ Cage **1** (blue solid line), Cage **1** with empty cavity (black dashed line), naphthalene ⊂ Cage **1** (red solid line) and total concentration of Cage **1** species (black solid line) in the aqueous layer during the forward and reverse transport for System 3. Data in **a-c** are presented as mean values +/- measurement errors, derived from error propagation of the SD and the signal-to-noise ratio (n=22) of coronene (Supplementary Section 5). The competitive displacement model thus predicts the qualitative behavior of the system when naphthalene is present. Considering naphthalene transport and competitive displacement add complexity to the system, leading to challenges in optimization. Our treatment using the Mathematica software package[6] was not able to fit the behavior of the system. Selection of the values of the kinetic constants is therefore based on qualitative fitting.

12. ***Trans*- and *cis*-FAB transport without cage 1**

To investigate the role of cage **1** in transporting FAB, a control experiment was conducted with pure D_2O in the membrane layer instead of the solution of cage **1**. Arm I, containing FAB (10 mM, 2 mL), coronene (0.25 mM) and triisopropylbenzne (10 mM), was exposed to 530 nm light, to generate *cis*-FAB. Arm II, containing FAB (10 mM, 2 mL) and coronene (0.25 mM), was irradiated with 400 nm light, to promote *trans*-FAB formation. ^{1}H NMR was used to monitor the change of FAB concentration in both arm I and arm II. It was observed that in the absence of cage **1** carriers, there was no noticeable change in the FAB concentration in both arms, consistent with the inference that the flow of the compounds between the arms was facilitated by cage **1**.

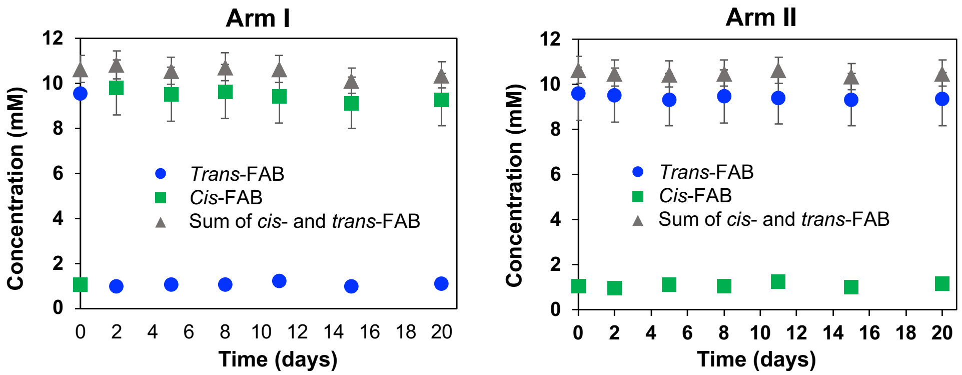

Supplementary Fig. 34. In the absence of cage **1**, little change of FAB isomers was observed in both arm I and arm II. We thus tributed the shift in the FAB concentration between the two arms to the isomer uptake and release by cage **1**. Data are presented as mean values +/- measurement errors, derived from error propagation of the SD and the signal-to-noise ratio (n=22) of coronene (Supplementary Section 5).

13. Independent transport of *trans*- and *cis*-FAB

Solutions of *trans*-FAB (90%) and *cis*-FAB (94%) (10 mM, 2 mL) in dodecane were introduced separately to arm I of two identical U-tubes (Supplementary Fig. 35). Aqueous solutions of cage **1** (4 mM, 2.5 mL, 50 mol% relative to the total FAB in both arms) were used as carriers to transport the isomers. Pure dodecane layers were inserted to arm II as receiving phases. Triisopropylbenzene (10 mM), which is not a guest for **1**, was added to arm I as an indicator to make sure that there was no mixing between arm I and arm II. For the experiment involving *trans*-FAB, the procedure was carried out in the dark to avoid *trans* to *cis* isomerization, making sure that cage **1** only transported *trans*-FAB, whereas both arm I and arm II in the experiment involving *cis*-FAB were irradiated at 530 nm to ensure that only *cis*-FAB was transported. The accumulation of the isomer in arm II was monitored by ^{1}H NMR integration, referencing to the internal standard coronene (0.25 mM).

The transport of *cis*-FAB in this independent transport experiment (Supplementary Fig. 35b) was slower than in the Maxwell's Demon experiment (Supplementary Fig. 13), where significant amount of *trans*-FAB was also present in the other arm, suggesting that competitive displacement is likely to be taking place in the Maxwell's Demon experiment. This observation was the inspiration for the design of the experiments in System 2 and 3.

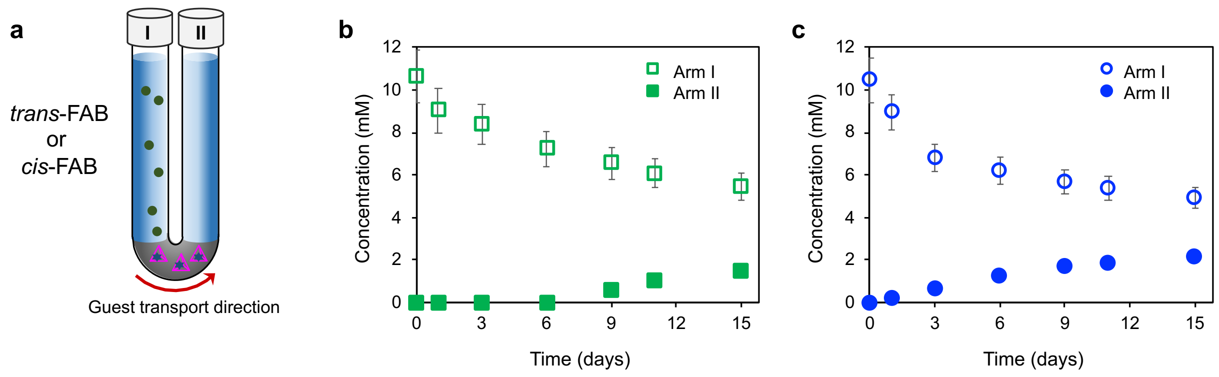

Supplementary Fig. 35. a, U-tube setup for the independent transport experiments. **b**, *Cis*-FAB concentrations in arm I and arm II monitored by ^{1}H NMR in the independent transport experiment of *cis*-FAB, where the U-tube was irradiated at 530 nm. **c**, *Trans*-FAB concentrations in arm I and arm II monitored by ^{1}H NMR in the independent transport experiment of *trans*-FAB, where the U-tube was left in the dark. Data are presented as mean values +/- measurement errors, derived from error propagation of the SD and the signal-to-noise ratio (n=22) of coronene (Supplementary Section 5).

14. Control experiment to confirm FAB transport in the presence of light

In this control experiment, two glass tubes (20 mL) were prepared, each containing a solution of cage **1** (2.5 mL, 4.0 mM in D_2O) and a FAB stock solution (2.0 mL, 10 mM in dodecane). The dodecane layer contained a 0.25 mM coronene internal standard for NMR analysis. Tube 1 was irradiated at 400 nm and Tube 2 at 530 nm. A magnetic stir bar was added into each tube and the solutions were stirred thoroughly during the experiments, ensuring good mixing between the D_2O and dodecane phases. Each tube was sealed with a fresh septum to avoid exposure to the external environment. Blue (400 nm) and green (530 nm) LED strips were rolled around Tube 1 and Tube 2, respectively; and each tube was kept in the dark with a black cloth covering it to avoid external light.

The NMR spectrum of the dodecane layer of each tube was taken after 6 hours and then 24 hours of irradiation, to measure the FAB concentration. For NMR measurements, samples of 300 μL were taken out from the dodecane layer of each tube. These solutions were placed into NMR tubes (outer diameter 5.0 mm, wall thickness 0.43 mm, length 180 mm). Sealed capillary tubes (outer diameter = 1.8 – 2.0 mm, wall thickness = 0.28 – 0.32 mm, length = 100 mm) containing D_2O were inserted into the NMR tubes to provide a deuterium signal for locking the spectrometer. After measurement, each samples was re-injected into the tubes from which it was taken. The solutions taken out were covered in aluminium foil to avoid external light outside of the NMR spectrometer. The time used for each measurement was minimized (<30 minutes), to avoid disturbing the experiment. All ^1H NMR spectra used for quantitative studies were referenced to coronene as the internal standard at 9.1 ppm. FAB concentration was determined following the procedure described in Supplementary Section 5.

These experiments showed the extent of FAB partitioning into the D_2O layer containing 4.0 mM cage 1, thus acting as a control experiment for System 1 in Fig. 1c. The decrease in the FAB concentration in Tube 1 (from 10.01±0.58 mM in the stock solution to 9.02±0.65 mM after 24 h irradiation at 400 nm, Supplementary Fig. 36c) supports the inference that mass transport takes place in System 1, resulting in the observed difference between FAB concentration between arm I and arm II (Fig. 2d), as opposed to the cage simply acting as a reservoir for FAB taken selectively from one of the two arms. Without active transport occurring, the FAB concentration in the arm irradiated at 400 nm in System 1 would not differ from that observed in Tube 1 in this control experiment (Supplementary Fig. 36a). Hence, the increase in FAB concentration in the arm irradiated at 400 nm in System 1 above the level observed in Tube 1 is inferred to result from the net transport of FAB from the other arm.

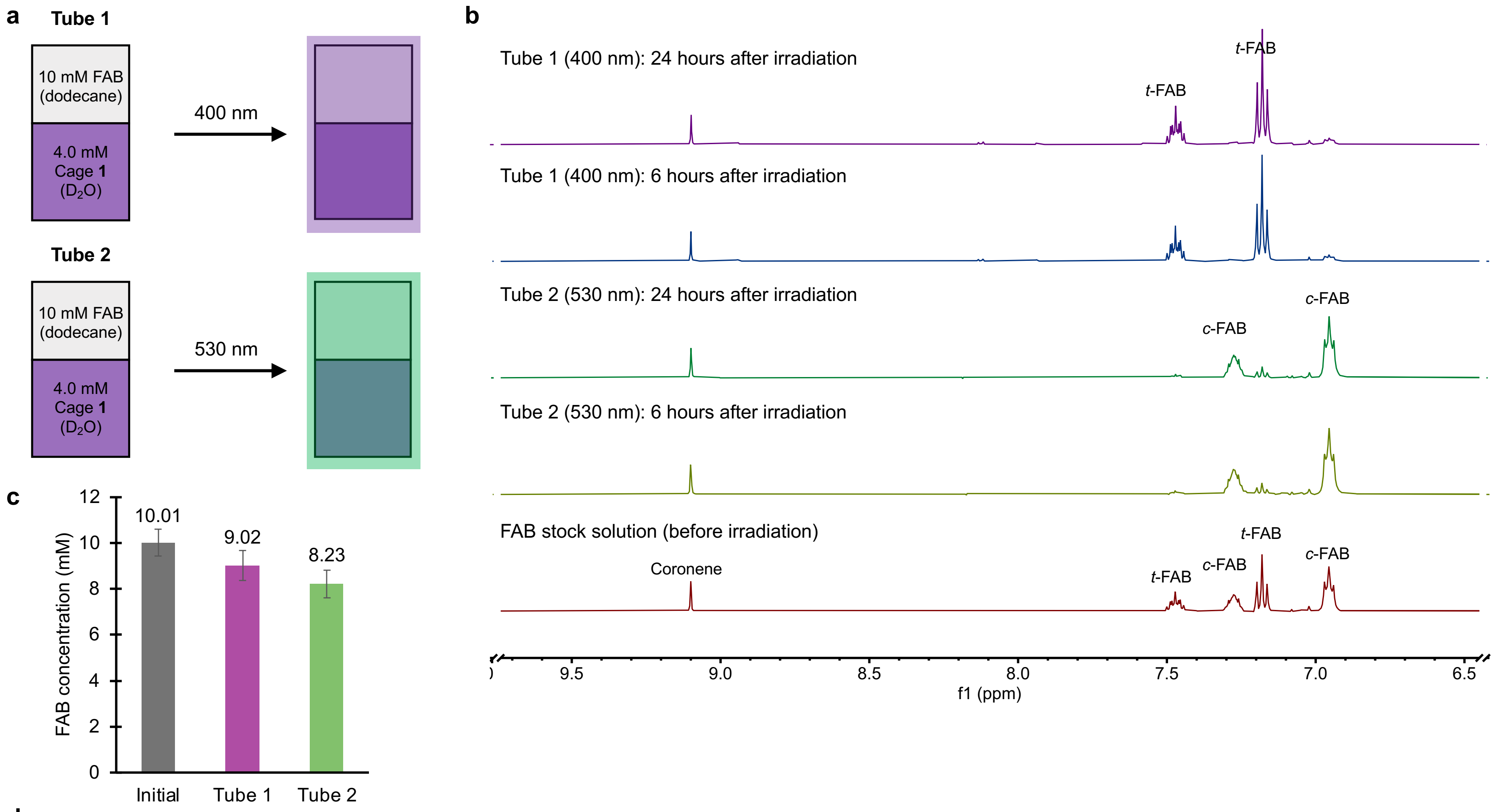

d

	FAB Partition Experiment																					
	Time (hour)	cis-FAB									trans-FAB									FAB (total)		
		∫Hj-cis	∂Hj-cis	∫Hk-cis	∂Hk-cis	Fcis	∂Fcis	**[cis-FAB]**	∂[cis-FAB]	%∂[cis-FAB]	∫Hj-trans	∂Hj-trans	∫Hk-trans	∂Hk-trans	Ftrans	∂Ftrans	**[trans-FAB]**	∂[trans-FAB]	%∂[trans-FAB]	**[FAB] (II)**	∂[FAB] (II)	%∂[FAB] (II)
**Tube 1 400 nm**	**0**	396.00	21.80	795.07	41.75	198.51	7.85	**5.96**	0.48	8.04	301.24	17.06	510.10	27.51	135.22	5.39	**4.06**	0.33	8.06	**10.01**	0.58	5.79
	**6**	73.30	5.66	140.82	9.04	35.69	1.78	**1.07**	0.09	8.59	534.74	28.74	1083.62	56.18	269.73	10.52	**8.09**	0.65	8.01	**9.16**	0.65	7.15
	**24**	70.72	5.54	132.38	8.62	33.85	1.71	**1.02**	0.09	8.63	533.29	28.66	1067.64	55.38	266.82	10.39	**8.00**	0.64	8.01	**9.02**	0.65	7.17
**Tube 2 530 nm**	**0**	396.00	21.80	795.07	41.75	198.51	7.85	**5.96**	0.48	8.04	301.24	17.06	510.10	27.51	135.22	5.39	**4.06**	0.33	8.06	**10.01**	0.58	5.79
	**6**	510.00	27.50	986.61	51.33	249.43	9.71	**7.48**	0.60	8.01	52.66	4.63	113.22	7.66	27.65	1.49	**0.83**	0.07	8.84	**8.31**	0.60	7.26
	**24**	506.78	27.34	981.63	51.08	248.07	9.66	**7.44**	0.60	8.01	49.61	4.48	107.46	7.37	26.18	1.44	**0.79**	0.07	8.90	**8.23**	0.60	7.29

Supplementary Fig. 36. a, Cartoon illustrating the setup for Tube 1 (400 nm irradiation) and Tube 2 (530 nm irradiation) in the FAB partitioning experiment. **b**, ^1H NMR spectra (500 MHz, D_2O, 298 K) of the measurements taken during the FAB partitioning experiment, showing ^1H NMR spectra of the FAB stock solution (10 mM in dodecane), the dodecane layer of Tubes 1 and 2, 6 and 24 h after irradiation. Signals corresponding to the internal standard coronene (0.25 mM), *cis*-FAB (*c*-FAB) and *trans*-FAB (*t*-FAB) are labelled. **c**, Sum of *trans*- and *cis*-FAB concentrations in the FAB stock solution (grey) and the dodecane layer of Tube 1 (purple) and Tube 2 (green) after 24 hours of irradiation. Data are presented as mean values +/- measurement errors, derived from error propagation of the SD and the signal-to-noise ratio (n=22) of coronene (Supplementary Section 5). **d**, Raw data from NMR integration, with uncertainties and *trans*- and *cis*-FAB concentration values for the FAB stock solution (10 mM at time 0), the dodecane layers of Tube 1 (purple background) and Tube 2 (green background) after 6 and 24 h of irradiation.

15. Photos of the experimental setup

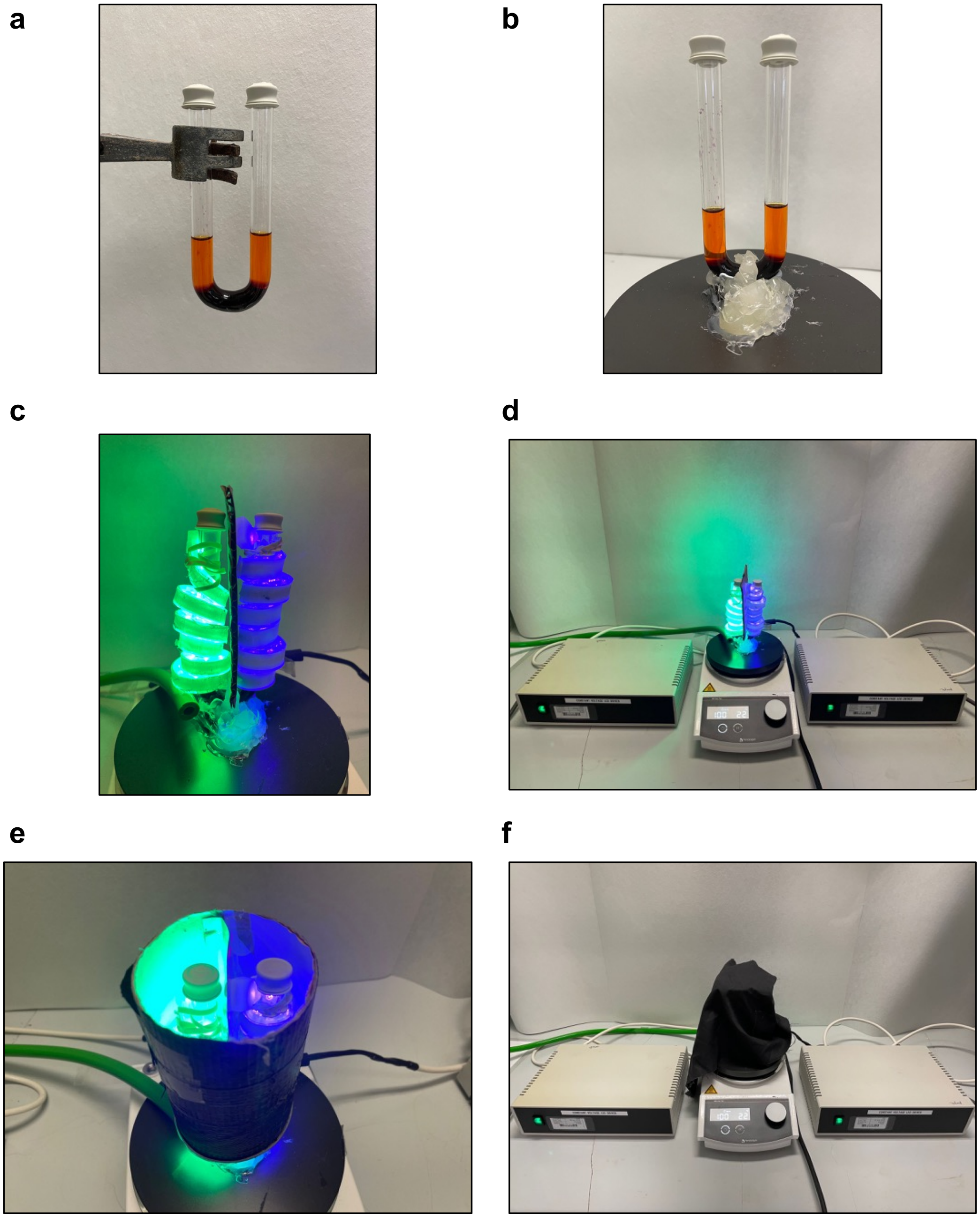

Supplementary Fig. 37. Photos showing the experimental setup. **a**, U-tube containing FAB solutions in arms I and II, separated by the aqueous layer at the bottom containing a solution of Cage **1**. A magnetic stir bar was placed in the U-tube and the tube was sealed with septa to avoid evaporation. **b**, The U-tube was glued onto the stir bar during the experiment, ensuring its vertical alignment. **c**, LED strips, 530 nm (green) and 400 nm (blue), were rolled around each arm of the U-tube. A separator was put between the arms to avoid light leakage from one side to the other. **d**, Zoom-out image from **c**, showing the LED strip drivers and the stirrer. **e**, The setup is put into a black cylindrical cover to prevent the ingress of external light, and to keep the tube stable. Nitrogen

flow (green tube) was provided inside this cover to provide cooling. **f**, The setup was then covered with a black cloth to prevent the ingress of external light.

References

1. Bolliger, J. L., Belenguer, A. M. & Nitschke, J. R. Enantiopure water-soluble [Fe_4L_6] cages: Host-guest chemistry and catalytic activity. *Angew. Chem. Int. Ed.* **52**, 7958–7962 (2013).
2. Nguyen, B. N. T. *et al.* Coordination cages selectively transport molecular cargoes across liquid membranes. *J Am Chem Soc* **143**, 12175–12180 (2021).
3. McLeod, K.; Comisarow, M. B. Systematic Errors in the Discrete Integration of FT NMR Spectra. *J. Magn. Reson*. **84**, 490–500 (1989).
4. Schönberger, T. *et al. Guide to NMR Method Development and Validation – Part I: Identification and Quantification***.** (Eurolab Technical Report 1/2023, 2023).
5. Billo, E. Joseph. *Excel for Chemists : a Comprehensive Guide*. (John Wiley & Sons, 2011).
6. Wolfram Research, Inc., *Mathematica, Version 14.0*, Champaign, IL (2024).
